# Gold(iii) tetraarylporphyrin amino acid derivatives: ligand or metal centred redox chemistry?†
†Electronic supplementary information (ESI) available: NMR and IR spectra, spectral changes upon reduction of **[Au(TPP)][PF_6_]**, **[4a][PF_6_]**, **[4b][PF_6_]**, **[4c][PF_6_]**, EPR spectra of **1a** in the absence and presence of chloride, DFT calculations of **Au(TPP)**, **Cu(TPP)**, **4a**, **4b** and **4c**, Cartesian coordinates of all optimised structures. See DOI: 10.1039/c5sc03429a


**DOI:** 10.1039/c5sc03429a

**Published:** 2015-10-26

**Authors:** Sebastian Preiß, Jascha Melomedov, Anica Wünsche von Leupoldt, Katja Heinze

**Affiliations:** a Institute of Inorganic Chemistry and Analytical Chemistry , Johannes Gutenberg-University of Mainz , Duesbergweg 10-14 , 55128 Mainz , Germany . Email: katja.heinze@uni-Mainz.de ; Fax: +49-6131-39-27277 ; Tel: +49-6131-39-25886

## Abstract

*Meso* tetraarylporphyrinato gold(iii) cations bearing different substituents at the aryl substituents (COOMe, COOH, NO_2_, NH_2_, NHAc, H, O^*n*^Bu, CF_3_) were prepared and characterised. Their reversible one-electron reductions were studied by (spectro)electrochemical means as well as by selective chemical one-electron reduction using cobaltocene. The preferred location of the spin density, namely gold centred or porphyrin centred, was probed by electron paramagnetic resonance spectroscopy (*g* values, ^197^Au hyperfine coupling) as well as by density functional theory calculations (spin densities). In all cases studied experimentally and theoretically, the gold(ii) valence isomer (5d^9^ electron configuration) is preferred over the porphyrin π radical anion. In the hexafluorophosphate salt of the nitro derivative a further nitro π radical anion valence isomeric species is significantly populated. In the presence of chloride ions this nitro π radical anion/Au^II^ valence isomeric equilibrium evolves towards the porphyrin π radical anion. The electronic structures of the nitro π radical and the Au^II^ σ radical valence isomers (5d_*x*^2^–*y*^2^_ orbital) could be calculated by DFT methods. The electron transfer pathway between the nitro π radical anion and the Au^II^ valence isomer is well described by the location of the hexfluorophosphate counterion, the Au–N distances (corresponding to the totally symmetric stretching vibration), the symmetric stretching mode of the NO_2_ substituent and a *meso*-nitrophenyl rotation. The specific geometric and electronic properties of the favoured gold(ii) σ radical valence isomer, namely counterion dislocation and σ symmetry of the redox orbital, might stabilise charge-shifted states [(gold(ii) porphyrin)-donor˙^+^] by retarding the back electron transfer to give the ground state (gold(iii) porphyrin)-donor. This will guide the design of (photo-induced) electron transfer pathways with tetraarylporphyrinato gold(iii) complexes as electron acceptors.

## Abstract 2

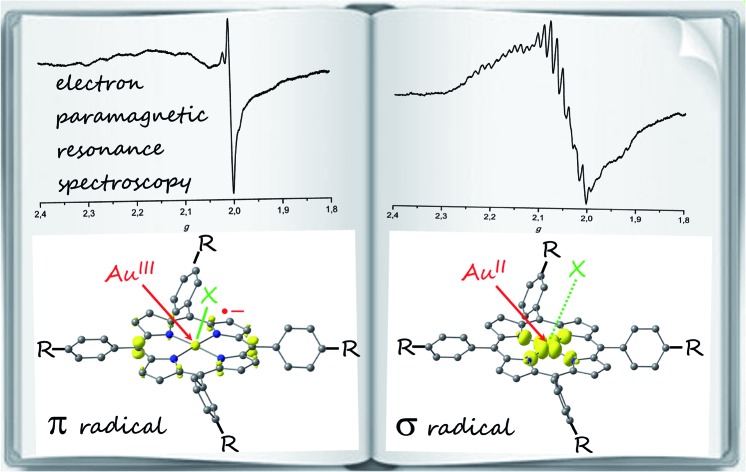
EPR spectroscopy and DFT calculations show that the site of reduction of porphyrinato gold(iii) complexes depends on the counterions X, the *meso* substituents R and the solvent.

## Introduction

Synthetic metallo porphyrins are of increasing interest due to their suitability as chromophores, as well as electron and hole acceptors in artificial photosynthetic systems,^1^ due to their catalytic and sensing properties,^2^ due to their medical applications^3^ as well as due to their propensity to stabilise unusual metal oxidation states. Specifically, porphyrinato gold(iii) complexes have evolved as efficient anticancer drugs.^4^ Furthermore, they are catalysts for the cycloisomerization of allenones.^5^ Recently, gold clusters with face-on coordinated free-base porphyrins have been reported.^6^ Finally, gold(iii) porphyrins are suitable ingredients in photoinduced electron transfer chains with the gold(iii) porphyrin acting as electron acceptor.^7^

**Scheme 1:**
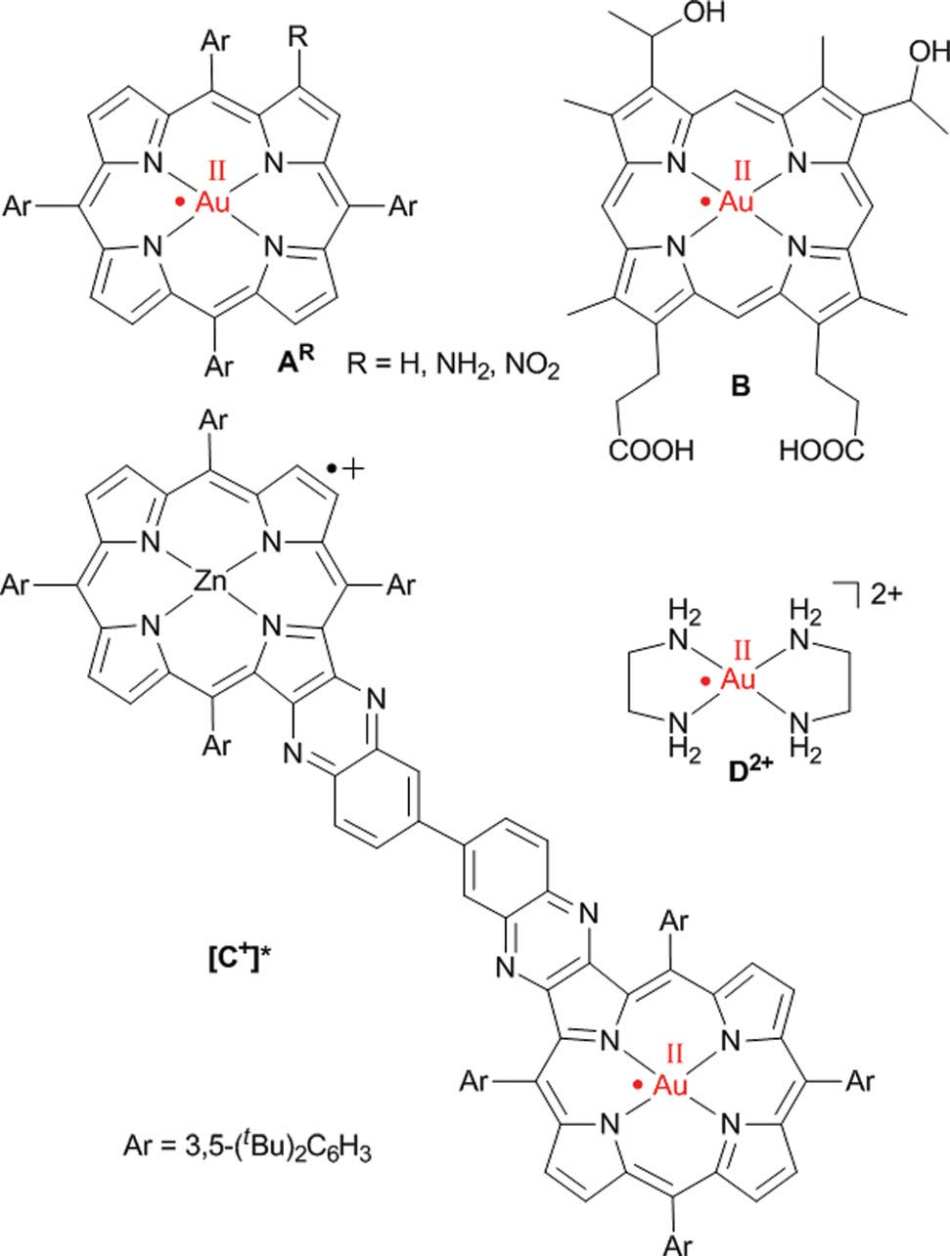
Genuine mononuclear gold(ii) complexes with planar N_4_ coordination according to EPR spectroscopic results.^9^,^13^,^15^

The site of gold(iii) porphyrin reduction, namely ligand or metal centred, has been discussed controversially. Based on early UV/Vis spectroscopic and theoretical studies the products of the reduction of gold(iii) porphyrins had been described as porphyrin-centred π radical anions.^8^ In a seminal paper, Kadish, Fukuzumi and Crossley provided compelling EPR spectroscopic evidence that the one-electron reduction of **[A^H^]^+^** to **A^H^** is metal centred giving gold(ii) porphyrins (Scheme 1).^9^ Only a few ligand types, such as thiolates or thioethers, are capable to stabilise mononuclear gold in the oxidation state +II.^10^ Further outstanding examples are the fluorosulfate^11^ and xenon complexes^12^ of Au^II^. Nitrogen donor ligands such as porphyrinato ligands have been reported to stabilise Au^II^ with respect to disproportionation and dimerization^9^,^13^ to [AuII2] species^14^ as well (Scheme 1, **A**, **B**, [C^+^]*). Disproportionation and dimerisation of [Au^II^(en)_2_]^2+^**D^2+^** has been suppressed by encapsulation in the pores of a zeolite (en = ethylenediamine).^15^

The gold(ii) porphyrin **A^H^** (Scheme 1) prepared by reduction of the corresponding gold(iii) porphyrin cation **[AH]^+^** with the strongly reducing naphthalene radical anion yielded a broad EPR resonance centred at *g*_av_ = 2.06.^9^ Hyperfine coupling to ^197^Au has been reported for the central *g* line [*A*(^197^Au) = 27 G at 113 K; *I*(^197^Au) = <svg xmlns="http://www.w3.org/2000/svg" version="1.0" width="16.000000pt" height="16.000000pt" viewBox="0 0 16.000000 16.000000" preserveAspectRatio="xMidYMid meet"><metadata> Created by potrace 1.16, written by Peter Selinger 2001-2019 </metadata><g transform="translate(1.000000,15.000000) scale(0.005147,-0.005147)" fill="currentColor" stroke="none"><path d="M400 2680 l0 -40 -40 0 -40 0 0 -40 0 -40 -80 0 -80 0 0 -80 0 -80 80 0 80 0 0 80 0 80 80 0 80 0 0 40 0 40 280 0 280 0 0 -80 0 -80 40 0 40 0 0 -80 0 -80 -40 0 -40 0 0 -40 0 -40 -120 0 -120 0 0 -40 0 -40 -80 0 -80 0 0 -40 0 -40 80 0 80 0 0 -40 0 -40 120 0 120 0 0 -40 0 -40 40 0 40 0 0 -80 0 -80 -80 0 -80 0 0 -80 0 -80 -240 0 -240 0 0 40 0 40 -80 0 -80 0 0 80 0 80 -80 0 -80 0 0 -80 0 -80 80 0 80 0 0 -40 0 -40 40 0 40 0 0 -40 0 -40 320 0 320 0 0 40 0 40 40 0 40 0 0 80 0 80 80 0 80 0 0 80 0 80 -40 0 -40 0 0 40 0 40 -40 0 -40 0 0 40 0 40 -120 0 -120 0 0 40 0 40 120 0 120 0 0 40 0 40 40 0 40 0 0 40 0 40 40 0 40 0 0 80 0 80 -40 0 -40 0 0 80 0 80 -40 0 -40 0 0 40 0 40 -360 0 -360 0 0 -40z M2640 2680 l0 -40 -40 0 -40 0 0 -40 0 -40 -40 0 -40 0 0 -40 0 -40 -40 0 -40 0 0 -40 0 -40 -40 0 -40 0 0 -40 0 -40 -40 0 -40 0 0 -40 0 -40 -40 0 -40 0 0 -40 0 -40 -40 0 -40 0 0 -40 0 -40 -40 0 -40 0 0 -40 0 -40 -40 0 -40 0 0 -40 0 -40 -40 0 -40 0 0 -40 0 -40 -40 0 -40 0 0 -40 0 -40 -40 0 -40 0 0 -40 0 -40 -40 0 -40 0 0 -40 0 -40 -40 0 -40 0 0 -40 0 -40 -40 0 -40 0 0 -40 0 -40 -40 0 -40 0 0 -40 0 -40 -40 0 -40 0 0 -40 0 -40 -40 0 -40 0 0 -40 0 -40 -40 0 -40 0 0 -40 0 -40 -40 0 -40 0 0 -40 0 -40 -40 0 -40 0 0 -40 0 -40 -40 0 -40 0 0 -40 0 -40 -40 0 -40 0 0 -40 0 -40 -40 0 -40 0 0 -40 0 -40 -40 0 -40 0 0 -40 0 -40 -40 0 -40 0 0 -40 0 -40 -40 0 -40 0 0 -40 0 -40 -40 0 -40 0 0 -40 0 -40 -40 0 -40 0 0 -40 0 -40 -40 0 -40 0 0 -40 0 -40 -40 0 -40 0 0 -40 0 -40 -40 0 -40 0 0 -40 0 -40 -40 0 -40 0 0 -40 0 -40 80 0 80 0 0 40 0 40 40 0 40 0 0 40 0 40 40 0 40 0 0 40 0 40 40 0 40 0 0 40 0 40 40 0 40 0 0 40 0 40 40 0 40 0 0 40 0 40 40 0 40 0 0 40 0 40 40 0 40 0 0 40 0 40 40 0 40 0 0 40 0 40 40 0 40 0 0 40 0 40 40 0 40 0 0 40 0 40 40 0 40 0 0 40 0 40 40 0 40 0 0 40 0 40 40 0 40 0 0 40 0 40 40 0 40 0 0 40 0 40 40 0 40 0 0 40 0 40 40 0 40 0 0 40 0 40 40 0 40 0 0 40 0 40 40 0 40 0 0 40 0 40 40 0 40 0 0 40 0 40 40 0 40 0 0 40 0 40 40 0 40 0 0 40 0 40 40 0 40 0 0 40 0 40 40 0 40 0 0 40 0 40 40 0 40 0 0 40 0 40 40 0 40 0 0 40 0 40 40 0 40 0 0 40 0 40 40 0 40 0 0 40 0 40 40 0 40 0 0 40 0 40 40 0 40 0 0 40 0 40 40 0 40 0 0 40 0 40 40 0 40 0 0 40 0 40 40 0 40 0 0 80 0 80 -40 0 -40 0 0 -40z M2080 1240 l0 -40 -80 0 -80 0 0 -40 0 -40 -80 0 -80 0 0 -80 0 -80 80 0 80 0 0 80 0 80 80 0 80 0 0 40 0 40 160 0 160 0 0 -40 0 -40 80 0 80 0 0 -200 0 -200 -80 0 -80 0 0 -40 0 -40 -80 0 -80 0 0 -40 0 -40 -80 0 -80 0 0 -40 0 -40 -80 0 -80 0 0 -40 0 -40 -40 0 -40 0 0 -40 0 -40 -40 0 -40 0 0 -160 0 -160 480 0 480 0 0 40 0 40 -400 0 -400 0 0 120 0 120 40 0 40 0 0 40 0 40 40 0 40 0 0 40 0 40 80 0 80 0 0 40 0 40 80 0 80 0 0 40 0 40 80 0 80 0 0 40 0 40 80 0 80 0 0 200 0 200 -80 0 -80 0 0 40 0 40 -80 0 -80 0 0 40 0 40 -160 0 -160 0 0 -40z"/></g></svg> , natural abundance 100%].^9^ An EPR resonance with a significantly smaller peak-to-peak distance was observed for the gold(ii) complex **B** of hematoporphyrin IX with *g*_⊥_ = 2.035, *g*_∥_ = 1.970 and *A*_⊥_(^197^Au) = *A*_∥_(^197^Au) = 15 G at 130 K suggesting a less pronounced metal character (Scheme 1).13b,e The charge-shifted state [C^+^]* of a Zn^II^–Au^III^ bis(tetraarylporphyrin) **C^+^** yielded an EPR resonance with *g* = 2.182, 2.043, 1.979 and *A*(^197^Au) = 180, 14, n.r. G in frozen toluene solution at 143 K for the gold(ii) centre (Scheme 1).13*d* The gold complex [Au(en)_2_]^2+^**D^2+^** with the pure σ-donor ligand ethylenediamine trapped in a zeolite shows *g*_∥_ = 2.239, *g*_⊥_ = 2.051, *A*_∥_(^197^Au) = 188 G and *A*_⊥_(^197^Au) = 22 G at room temperature (Scheme 1).^15^ Gold(iii)-centred reductions have been associated with a significantly higher reorganization energy (*ca.* 1.25 eV) than porphyrin-based reductions (*ca.* 0.6 eV).13*c* The large reorganization energy renders gold(iii) porphyrins suitable electron acceptors in photoelectron transfer schemes.^7^ Moreover, gold(iii)-associated counterions should dissociate upon Au^III^ to Au^II^ reduction further retarding the back-electron transfer. For instance, chloride is associated to the Au^III^ centre in solid **AuCl(TPP)** by electrostatic forces with a gold chloride distance of 3.01(1) Å.^16^ Unfortunately, no solid structures of porphyrinato gold(ii) complexes have been reported so far and further experimental or theoretical studies are lacking.

We had previously reported synthetically versatile *meso*-substituted tetraaryl porphyrins with *trans*-AB_2_C substitution pattern including A = nitrophenyl, aminophenyl or amidophenyl, C = phenyl carboxylic acid or ester and B = EWG or EDG substituted aryl groups.^17^ These porphyrins can be metallated^17^ and assembled to multiporphyrin amides,17b,17c electron donor substituted amide-linked dyads17*a* as well as electron donor (ferrocene) and electron acceptor (quinone) substituted amide-linked triads and tetrads17*b* with well defined sequences from the N-terminus to the C-terminus. The different *meso* substituents of the porphyrin amino acids at the B position can be used to modulate the solubilities and to fine-tune the redox potentials which allows to design redox gradients.^17^

With this family of porphyrins in hand, we disclose in this contribution the factors that control the relative stabilities of a gold(ii) porphyrin and its valence isomeric gold(iii) porphyrin radical anion. We report novel *meso*-substituted Au^III^ porphyrin amino acid derivatives with *trans*-AB_2_C substitution pattern for potential incorporation into electron transfer chains *via* amide bonds. These gold(iii) porphyrins were inspected by cyclic voltammetry, UV/Vis spectroelectrochemistry and by EPR spectroscopy upon selective one-electron reduction with cobaltocene. We provide strong EPR and UV/Vis spectroscopic evidence that all singly reduced gold(iii) porphyrins are well described as gold(ii) porphyrins essentially irrespective of the *meso*-substituents A, B and C and that the porphyrin radical anions are higher energy valence tautomers of the ground state Au^II^ valence isomers. Detailed EPR parameters of the gold(ii) porphyrinato complexes were obtained by spectral simulations of the experimental spectra (*g* tensors, (super)hyperfine couplings, valence isomer ratios). The experimental data are corroborated and interpreted with the aid of density functional theory (DFT) calculations in the framework of electron transfer theory.

## Results and discussion

### Synthesis of free-base porphyrins and (porphyrinato)gold(iii) complexes (series **[1a]^+^–[3a]^+^** and series **[4a]^+^–[4c]^+^**)

**Scheme 2:**
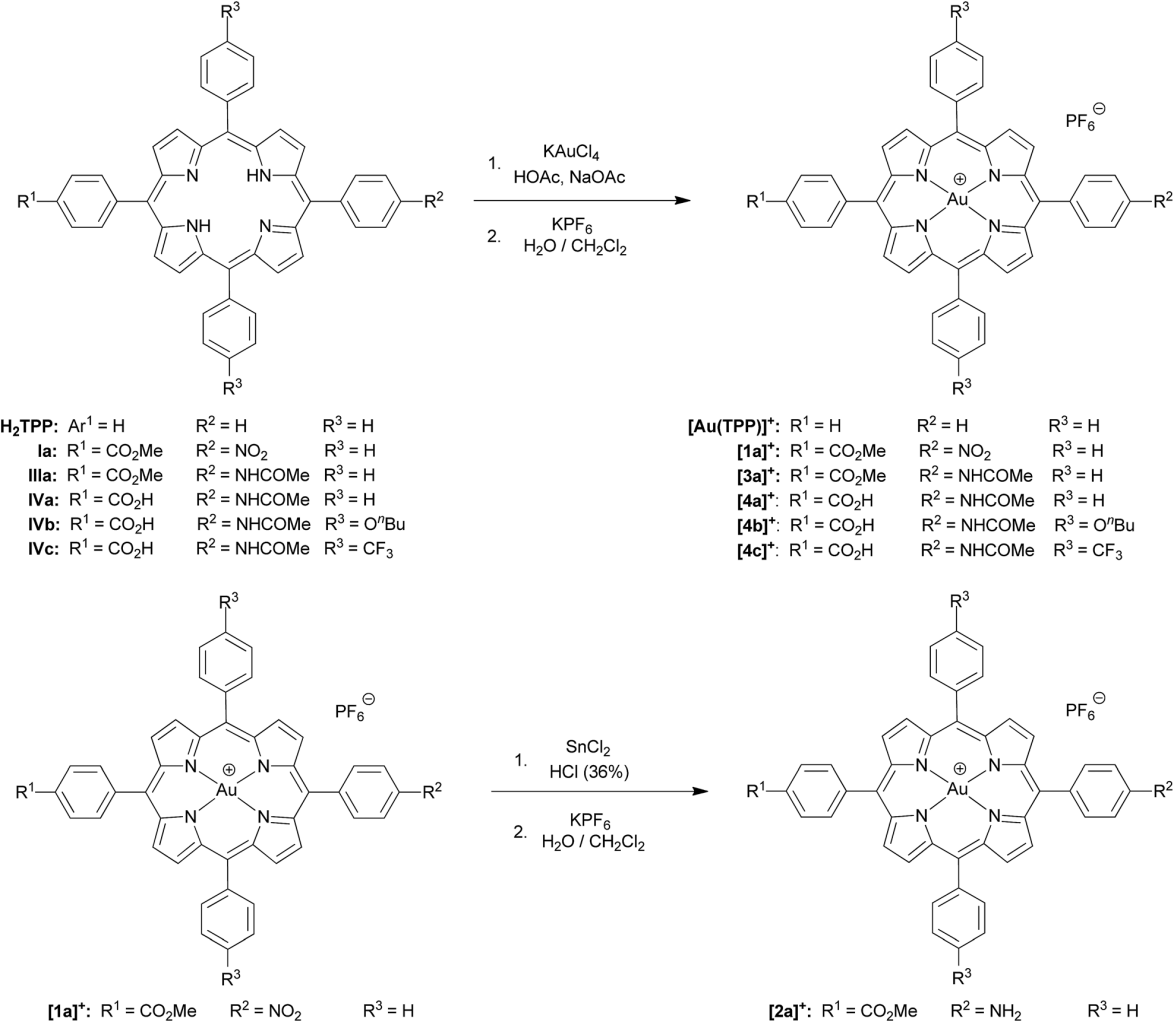
Synthesis of (porphyrinato)gold(iii) complexes (**[Au(TPP)]^+^**, series **[1a]^+^**, **[2a]^+^**, **[3a]^+^** and series **[4a]^+^**, **[4b]^+^**, **[4c]^+^**).

The free-base porphyrins **Ia–IVc** were prepared according to literature procedures.^17^ Metallation of the free-base porphyrins was successful with potassium tetrachlorido aurate(iii) in the presence of HOAc/NaOAc (Fleischer's route^18^), except for amino-substituted porphyrin **IIa** (Scheme 2). Best yields were obtained using four equivalents of KAuCl_4_ giving the cationic aurated porphyrins as poorly soluble tetrachlorido aurate salts.

Auration of the amino-substituted porphyrin **IIa** with KAuCl_4_/HOAc/NaOAc according to Fleischer's method resulted in acylation of the amino group. Auration of **IIa** according to Sauvage's protocol using [Au^I^(tht)_2_][BF_4_] followed by disproportionation to Au^III^ and Au^0^ was unsuccessful as well (tht = tetrahydrothiophene).^19^ Thus, **[2a]^+^** was prepared *via* metallation and ion exchange of nitroporphyrin ester **Ia** to give **[1a][PF_6_]**, followed by reduction of the nitro group of **[1a]^+^** with SnCl_2_/HCl to give the aurated amino-substituted porphyrin **[2a]Cl** (Scheme 2). During this procedure, the gold(iii) ion was neither reduced nor removed. Hence, the Au^III^ porphyrins are stable under protic conditions. Counterion exchange of [Au(porph)][AuCl_4_] or **[2a]Cl** with KPF_6_ yielded the corresponding soluble hexafluorophosphate salts which are conveniently purified by column chromatography.

### Characterization of (porphyrinato)gold(iii) complexes (series **[1a]^+^–[3a]^+^** and series **[4a]^+^–[4c]^+^**)

Ester-substituted complexes **[1a][PF6]–[3a][PF6]** are sufficiently soluble in dichloromethane. However, THF is required for acids **[4b][PF_6_]** and **[4c][PF_6_]** and even methanol is necessary for **[4a][PF_6_]** in order to acquire NMR spectra with a satisfactory signal-to-noise ratio. This shows that the counterion and the *meso* substituents determine the solubility. All gold(iii) complexes were characterised by ^1^H NMR, ^13^C NMR, ^31^P NMR and 2D NMR spectroscopy, IR spectroscopy and high-resolution mass spectrometry. The proton NMR spectra display the expected number and intensities of resonances. The chemical shifts vary according to the substitution patterns paralleling the shifts of the corresponding free-base porphyrins **Ia–IIIa** and **IVa–IVc**. The CH_3_-ester, NH_2_-amine and CH_3_-amide substituents display characteristic resonances at *δ* = 4.1, 4.7, 2.2 ppm, respectively. The [PF_6_]^–^ counterions show the characteristic septet at *δ* = –144 ppm in the ^31^P NMR spectra. Upon auration the characteristic high-field pyrrol NH resonances of the free-base porphyrin disappear. Furthermore, auration of the free-base porphyrins consistently shifts the pyrrole CH proton resonances to lower field by 0.5 ppm, in accordance with the positive charge of the metal centre. In the IR spectra, characteristic absorptions for group vibrations are found for the ester, amine, amide, nitro, trifluoromethyl and acid substituents at around 1719, 1618, 1690, 1520/1346, 1324 and 1716 cm^–1^, respectively. The [PF_6_]^–^ counterions display absorptions for the PF stretching and deformation modes at 835–843 and 556–558 cm^–1^, respectively. ESI mass spectra fully confirm the integrity and stability of the complex cations displaying peaks at *m*/*z* values corresponding to the intact complex cation (see Exp. section).

With the exception of the electron-rich R^3^ = O^*n*^Bu-substituted complex **[4b]^+^** all gold(iii) porphyrinato complexes **[1a]^+^**, **[2a]^+^**, **[3a]^+^**, **[4a]^+^** and **[4c]^+^** show hypsochromically shifted Soret bands as compared to their corresponding free-base porphyrins **Ia**, **IIa**, **IIIa**, **IVa** and **IVc** (*hypso* porphyrins8*b*). In all cases, the number of Q bands is reduced from four to two (or even to one) as expected for metalloporphyrins with local *D*_4h_ symmetry of the porphyrin core. Expectedly, gold(iii) porphyrinato complexes are non-emissive at room temperature in fluid solution as exemplarily checked for **[1a][PF_6_]**, **[3a][PF_6_]** and **[4c][PF_6_]**.8b,^20^

### Redox chemistry of (porphyrinato)gold(iii) complexes (series **[1a]^+^–[3a]^+^** and series **[4a]^+^–[4c]^+^**)

**Fig. 1:**
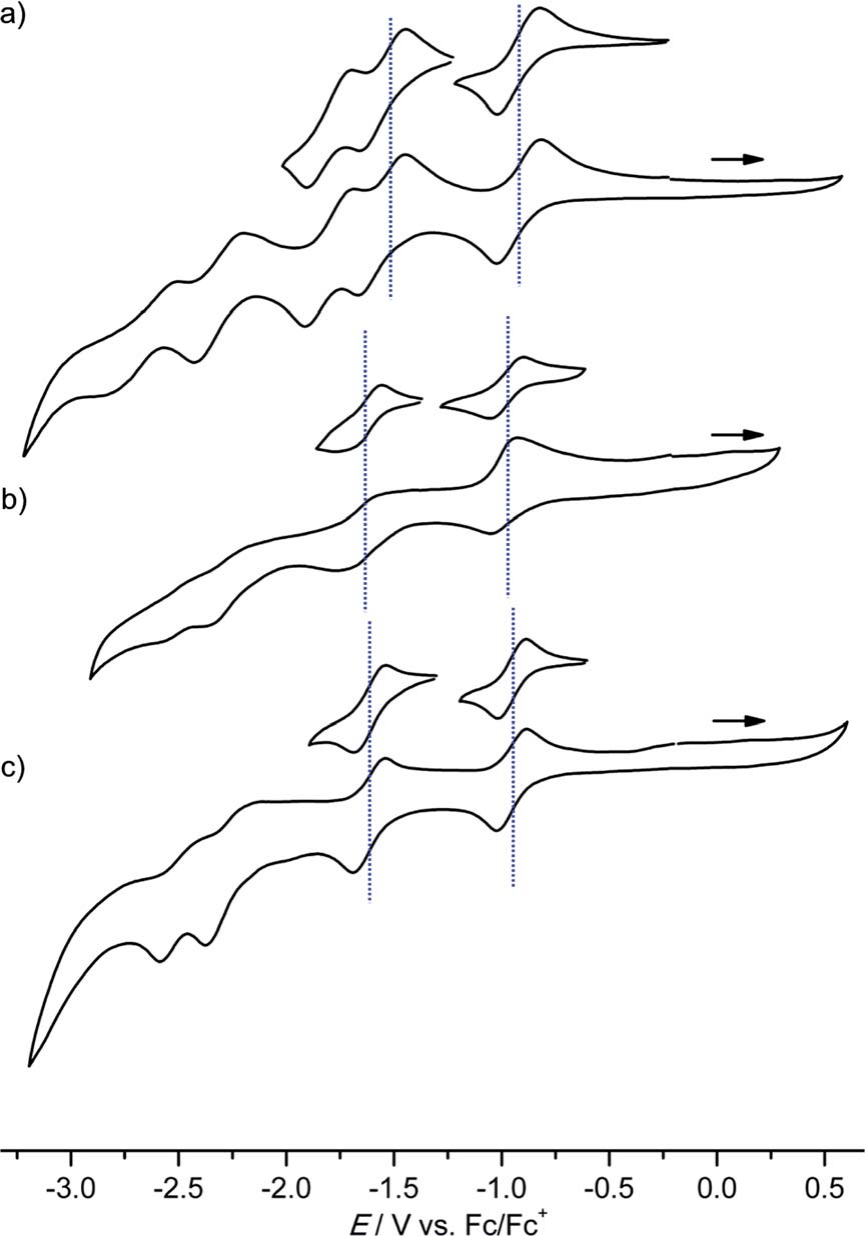
Cyclic voltammograms of (a) **[1a]^+^**, (b) **[2a]^+^** and (c) **[3a]^+^** 10^–3^ M in 0.1 M [^*n*^Bu_4_N][PF_6_]/THF solution; potentials referenced against the ferrocene/ferrocenium couple.

**Fig. 2:**
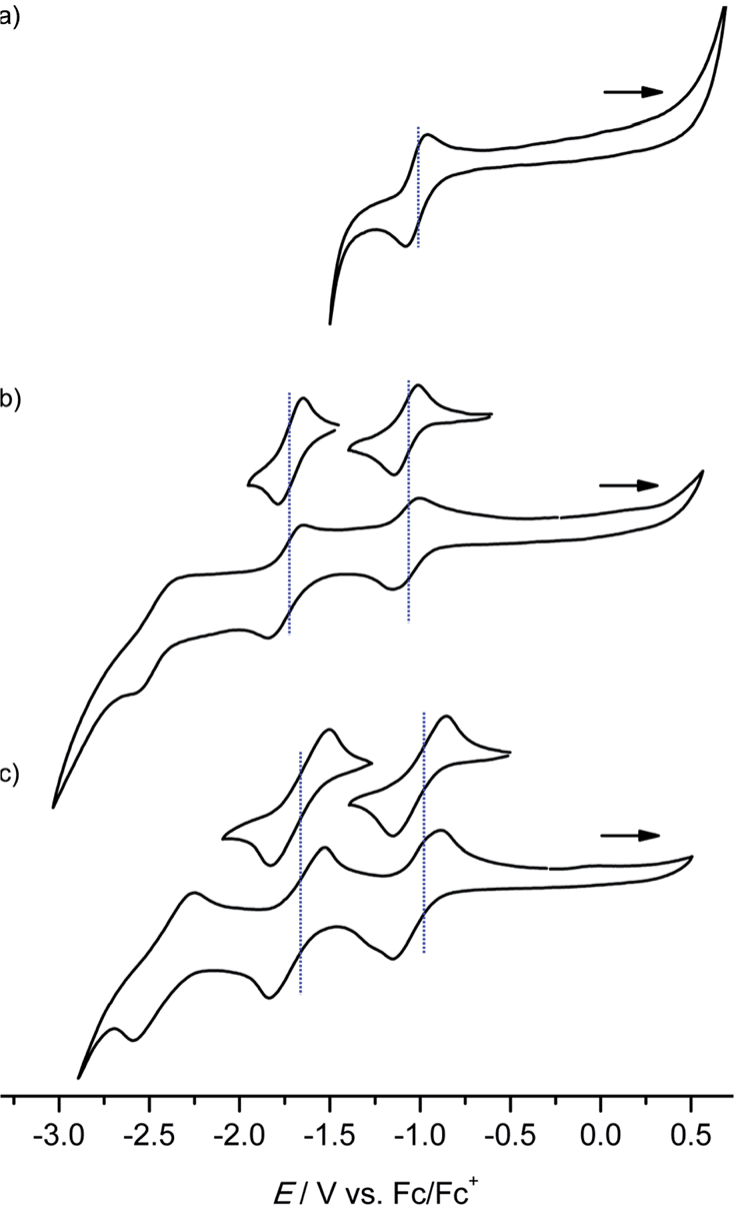
Cyclic voltammograms of (a) **[4a]^+^** (MeOH), (b) **[4b]^+^** and (c) **[4c]^+^** 10^–3^ M in 0.1 M [^*n*^Bu_4_N][PF_6_]/THF solution; potentials referenced against the ferrocene/ferrocenium couple.

Several reversible reductions are observed for cations **[1a]^+^**, **[2a]^+^**, **[3a]^+^**, **[4b]^+^** and **[4c]^+^** 10^–3^ M in 0.1 M [^*n*^Bu_4_N][PF_6_]/THF solution (Fig. 1 and 2, Table 1). For solubility reasons complex **[4a]^+^** was measured in MeOH and the solvent window of MeOH allows for only a single reversible reduction wave to be observed (Fig. 2, Table 1). All potentials are given relative to the ferrocene/ferrocenium couple. As expected from substituent effects, **[1a]^+^** is more easily reduced to **1a** (–0.92 V) than **[Au(TPP)]^+^** to **Au(TPP)** (–0.97 V), while **[2a]^+^** (–0.99 V) is more difficult to reduce. Similarly, the potentials shift to more negative values in the series **[4c]^+^** (–1.00 V), **[4a]^+^** (–1.02 V) and **[4b]^+^** (–1.08 V), which is again explicable by the increasing electron donating nature of the substituents (CF_3_, H, O^*n*^Bu). Similar to the corresponding free-base porphyrins the shifts are only small.^17^ The second reduction is especially facile with the electron withdrawing NO_2_ substituent (**1a**/**[1a]^–^**; –1.55 V). The nitro derivative **[1a]^+^** shows even further reversible reductions. Hence, one of the **[1a]^+^** reductions might be associated to the nitro substituent itself (*vide infra*).

**Table 1:** Redox potentials (peak potentials in parentheses) of porphyrinato gold(iii) complexes measured 10^–3^ M in 0.1 M [^*n*^Bu_4_N][PF_6_]/THF solution, potentials given relative to ferrocene/ferrocenium

	*E*_½_ ([Au(P)]^+^/Au(P))	*E*_½_ (Au(P)/[Au(P)]^–^)	*E*_½_ ([Au(P)]^–^/[Au(P)]^2–^)	*E*_½_ ([Au(P)]^2–^/[Au(P)]^3–^)
**[Au(TPP)][PF_6_]**	–0.97 (–1.06/–0.88)	–1.65 (–1.73/–1.57)	–2.34 (–2.43/–2.25)	—
**1a[PF_6_]**	–0.92 (–1.02/–0.82)	–1.55 (–1.66/–1.44)	–1.80 (–1.90/–1.70)	–2.31 (–2.42/–2.20)
**2a[PF_6_]**	–0.99 (–1.06/–0.91)	–1.67 (–1.78/–1.56)	–2.27 (–2.38/–2.16)	–2.51 (–2.56/–2.45)
**3a[PF_6_]**	–0.96 (–1.02/–0.89)	–1.63 (–1.70/–1.55)	–2.28 (–2.37/–2.19)	–2.50 (–2.58/–2.41)
**4a[PF_6_]** ^*a*^	–1.02 (–1.08/–0.96)	^*b*^	^*b*^	^*b*^
**4b[PF_6_]**	–1.08 (–1.15/–1.01)	–1.72 (–1.79/–1.65)	–2.46 (–2.57/–2.34)	
**4c[PF_6_]**	–1.00 (–1.15/–0.87)	–1.67 (–1.83/–1.50)	–2.42 (–2.58/–2.25)	

^*a*^In MeOH.

^*b*^Outside solvent window.

The differences between the first and second reduction potentials amount to 0.60–0.68 V which corresponds to very high comproportionation constants of *K*_C_ > 10^10^ for the neutral complexes.^21^ Hence, disproportionation of the neutral complexes into the corresponding cations and anions can be safely neglected and spectral signatures after one-electron reduction will essentially be associated with the neutral complexes.

**Fig. 3:**
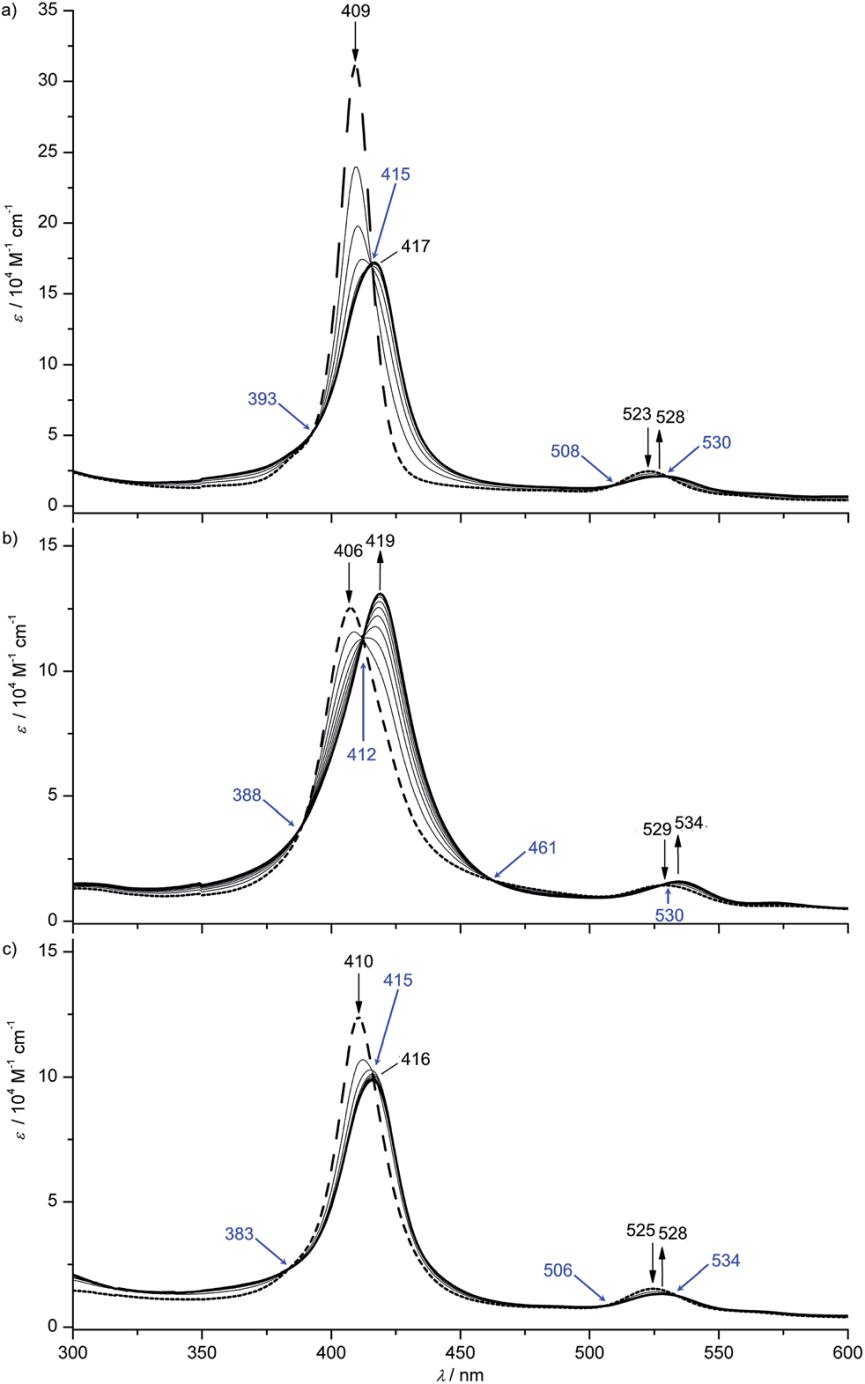
UV/Vis spectral changes upon the first electroreduction of (a) **[1a]^+^**, (b) **[2a]^+^** and (c) **[3a]^+^** in 0.1 M [^*n*^Bu_4_N][PF_6_]/THF solution (isosbestic points indicated in blue).

All gold(iii) complexes were reduced electrochemically to the neutral species in an optically transparent thin layer electrochemical (OTTLE) cell using THF as solvent (MeOH for **[4a]^+^**). In all cases, isosbestic points were observed corroborating the reversible nature of the first reduction process (Fig. 3 and ESI†). The shifts of the Soret and Q bands as well as the observed isosbestic points closely resemble those found for the **[Au(TPP)]^+^**/**Au(TPP)** process in THF (ESI†) and in pyridine13*c* or in PhCN.^9^ In all cases, except for the **[2a]^+^**/**2a** and **[4b]^+^**/**4b** redox couples with the strongly electron-donating NH_2_ and O^*n*^Bu substituents, the intensity of the Soret band decreases while for **[2a]^+^**/**2a** and **[4b]^+^**/**4b** the intensity increases (Fig. 3b and ESI†). A similar hyperchromic effect has been observed for the **[A^NH2^]^+^**/**A^NH2^** couple with the amino group attached to a porphyrin *beta* position.13*c*

For chemical reductions, the gold(iii) porphyrin complexes were dissolved in CH_2_Cl_2_ (**[1a][PF_6_]–[1c][PF_6_]**), THF (**[4b][PF_6_]**/**[4c][PF_6_]**) or MeOH (**[4a][PF_6_]**) (*ca.* 5 mM). In order to definitely prevent overreduction, these solutions were treated with slightly substoichiometric amounts of CoCp_2_ (*E*_½_ = –1.33 V in CH_2_Cl_2_*vs.* Fc/Fc^+^ (ref. 22)) in an EPR tube. The redox potential of CoCp_2_ is perfectly in-between the first and second reduction of the gold porphyrins (Fig. 1 and 2) further avoiding over-reduction. The reaction mixture in the tube was immediately frozen by immersing into liquid nitrogen and subjected to X-band EPR spectroscopy. Hence, we obtained significantly better resolved EPR spectra than previously reported for neutral porphyrinato gold complexes prepared by reduction of **[A^H^]^+^** with the strongly reducing naphthalene radical anion in DMF (*ca.* –3 V *vs.* Fc/Fc^+^ (ref. 22)).^9^ In this case some over-reduction might have been occurred blurring the hyperfine couplings to gold and nitrogen nuclei.

**Fig. 4:**
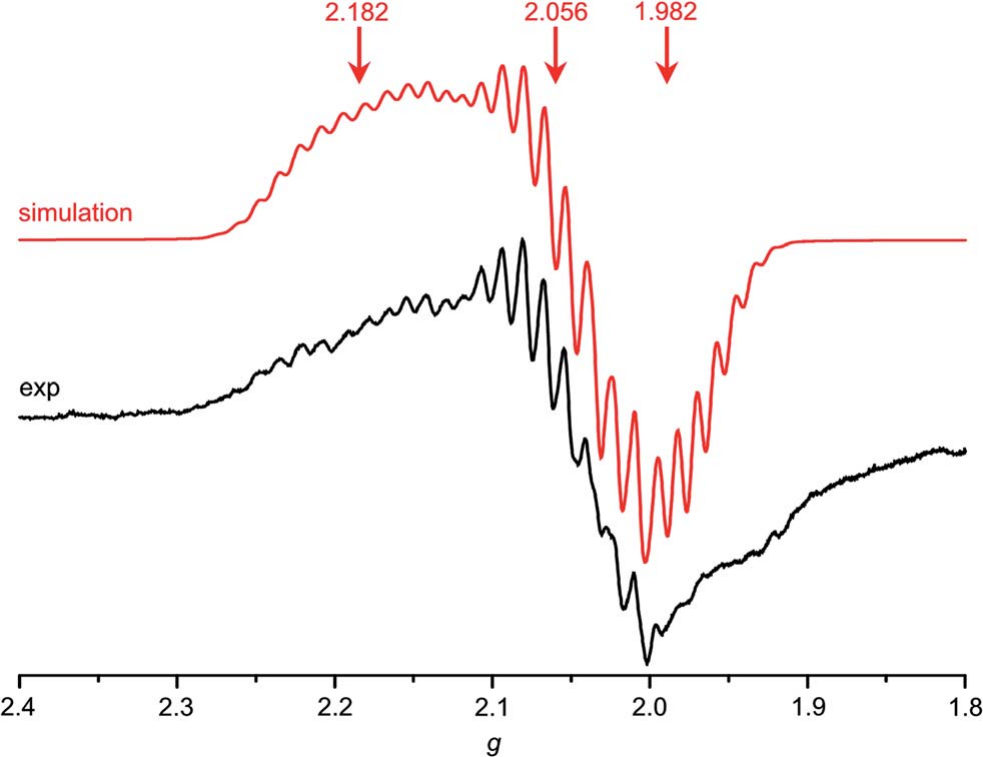
X-band EPR spectrum of **Au(TPP)** in frozen CH_2_Cl_2_ solution (77 K, 9.4 GHz) and corresponding simulation.

Indeed, **Au(TPP)** as prepared by reduction of **[Au(TPP)]^+^** by CoCp_2_ in CH_2_Cl_2_ shows a well-resolved EPR pattern which could be reasonably simulated by a rhombic *g* tensor with hyperfine interaction to a single ^197^Au nucleus (*I* = <svg xmlns="http://www.w3.org/2000/svg" version="1.0" width="16.000000pt" height="16.000000pt" viewBox="0 0 16.000000 16.000000" preserveAspectRatio="xMidYMid meet"><metadata> Created by potrace 1.16, written by Peter Selinger 2001-2019 </metadata><g transform="translate(1.000000,15.000000) scale(0.005147,-0.005147)" fill="currentColor" stroke="none"><path d="M400 2680 l0 -40 -40 0 -40 0 0 -40 0 -40 -80 0 -80 0 0 -80 0 -80 80 0 80 0 0 80 0 80 80 0 80 0 0 40 0 40 280 0 280 0 0 -80 0 -80 40 0 40 0 0 -80 0 -80 -40 0 -40 0 0 -40 0 -40 -120 0 -120 0 0 -40 0 -40 -80 0 -80 0 0 -40 0 -40 80 0 80 0 0 -40 0 -40 120 0 120 0 0 -40 0 -40 40 0 40 0 0 -80 0 -80 -80 0 -80 0 0 -80 0 -80 -240 0 -240 0 0 40 0 40 -80 0 -80 0 0 80 0 80 -80 0 -80 0 0 -80 0 -80 80 0 80 0 0 -40 0 -40 40 0 40 0 0 -40 0 -40 320 0 320 0 0 40 0 40 40 0 40 0 0 80 0 80 80 0 80 0 0 80 0 80 -40 0 -40 0 0 40 0 40 -40 0 -40 0 0 40 0 40 -120 0 -120 0 0 40 0 40 120 0 120 0 0 40 0 40 40 0 40 0 0 40 0 40 40 0 40 0 0 80 0 80 -40 0 -40 0 0 80 0 80 -40 0 -40 0 0 40 0 40 -360 0 -360 0 0 -40z M2640 2680 l0 -40 -40 0 -40 0 0 -40 0 -40 -40 0 -40 0 0 -40 0 -40 -40 0 -40 0 0 -40 0 -40 -40 0 -40 0 0 -40 0 -40 -40 0 -40 0 0 -40 0 -40 -40 0 -40 0 0 -40 0 -40 -40 0 -40 0 0 -40 0 -40 -40 0 -40 0 0 -40 0 -40 -40 0 -40 0 0 -40 0 -40 -40 0 -40 0 0 -40 0 -40 -40 0 -40 0 0 -40 0 -40 -40 0 -40 0 0 -40 0 -40 -40 0 -40 0 0 -40 0 -40 -40 0 -40 0 0 -40 0 -40 -40 0 -40 0 0 -40 0 -40 -40 0 -40 0 0 -40 0 -40 -40 0 -40 0 0 -40 0 -40 -40 0 -40 0 0 -40 0 -40 -40 0 -40 0 0 -40 0 -40 -40 0 -40 0 0 -40 0 -40 -40 0 -40 0 0 -40 0 -40 -40 0 -40 0 0 -40 0 -40 -40 0 -40 0 0 -40 0 -40 -40 0 -40 0 0 -40 0 -40 -40 0 -40 0 0 -40 0 -40 -40 0 -40 0 0 -40 0 -40 -40 0 -40 0 0 -40 0 -40 -40 0 -40 0 0 -40 0 -40 -40 0 -40 0 0 -40 0 -40 -40 0 -40 0 0 -40 0 -40 -40 0 -40 0 0 -40 0 -40 -40 0 -40 0 0 -40 0 -40 -40 0 -40 0 0 -40 0 -40 80 0 80 0 0 40 0 40 40 0 40 0 0 40 0 40 40 0 40 0 0 40 0 40 40 0 40 0 0 40 0 40 40 0 40 0 0 40 0 40 40 0 40 0 0 40 0 40 40 0 40 0 0 40 0 40 40 0 40 0 0 40 0 40 40 0 40 0 0 40 0 40 40 0 40 0 0 40 0 40 40 0 40 0 0 40 0 40 40 0 40 0 0 40 0 40 40 0 40 0 0 40 0 40 40 0 40 0 0 40 0 40 40 0 40 0 0 40 0 40 40 0 40 0 0 40 0 40 40 0 40 0 0 40 0 40 40 0 40 0 0 40 0 40 40 0 40 0 0 40 0 40 40 0 40 0 0 40 0 40 40 0 40 0 0 40 0 40 40 0 40 0 0 40 0 40 40 0 40 0 0 40 0 40 40 0 40 0 0 40 0 40 40 0 40 0 0 40 0 40 40 0 40 0 0 40 0 40 40 0 40 0 0 40 0 40 40 0 40 0 0 40 0 40 40 0 40 0 0 40 0 40 40 0 40 0 0 40 0 40 40 0 40 0 0 40 0 40 40 0 40 0 0 40 0 40 40 0 40 0 0 80 0 80 -40 0 -40 0 0 -40z M2080 1240 l0 -40 -80 0 -80 0 0 -40 0 -40 -80 0 -80 0 0 -80 0 -80 80 0 80 0 0 80 0 80 80 0 80 0 0 40 0 40 160 0 160 0 0 -40 0 -40 80 0 80 0 0 -200 0 -200 -80 0 -80 0 0 -40 0 -40 -80 0 -80 0 0 -40 0 -40 -80 0 -80 0 0 -40 0 -40 -80 0 -80 0 0 -40 0 -40 -40 0 -40 0 0 -40 0 -40 -40 0 -40 0 0 -160 0 -160 480 0 480 0 0 40 0 40 -400 0 -400 0 0 120 0 120 40 0 40 0 0 40 0 40 40 0 40 0 0 40 0 40 80 0 80 0 0 40 0 40 80 0 80 0 0 40 0 40 80 0 80 0 0 40 0 40 80 0 80 0 0 200 0 200 -80 0 -80 0 0 40 0 40 -80 0 -80 0 0 40 0 40 -160 0 -160 0 0 -40z"/></g></svg> ; natural abundance 100%) and superhyperfine coupling to four ^14^N nuclei (*I* = 1, natural abundance 99.6%). The high resolution allows a very good estimation of the high-field parameters while the low-field parameters are less well-resolved (Table 2, Fig. 4). Compared to the isoelectronic **Cu(TPP)** complex (^63/65^Cu; *I* = <svg xmlns="http://www.w3.org/2000/svg" version="1.0" width="16.000000pt" height="16.000000pt" viewBox="0 0 16.000000 16.000000" preserveAspectRatio="xMidYMid meet"><metadata> Created by potrace 1.16, written by Peter Selinger 2001-2019 </metadata><g transform="translate(1.000000,15.000000) scale(0.005147,-0.005147)" fill="currentColor" stroke="none"><path d="M400 2680 l0 -40 -40 0 -40 0 0 -40 0 -40 -80 0 -80 0 0 -80 0 -80 80 0 80 0 0 80 0 80 80 0 80 0 0 40 0 40 280 0 280 0 0 -80 0 -80 40 0 40 0 0 -80 0 -80 -40 0 -40 0 0 -40 0 -40 -120 0 -120 0 0 -40 0 -40 -80 0 -80 0 0 -40 0 -40 80 0 80 0 0 -40 0 -40 120 0 120 0 0 -40 0 -40 40 0 40 0 0 -80 0 -80 -80 0 -80 0 0 -80 0 -80 -240 0 -240 0 0 40 0 40 -80 0 -80 0 0 80 0 80 -80 0 -80 0 0 -80 0 -80 80 0 80 0 0 -40 0 -40 40 0 40 0 0 -40 0 -40 320 0 320 0 0 40 0 40 40 0 40 0 0 80 0 80 80 0 80 0 0 80 0 80 -40 0 -40 0 0 40 0 40 -40 0 -40 0 0 40 0 40 -120 0 -120 0 0 40 0 40 120 0 120 0 0 40 0 40 40 0 40 0 0 40 0 40 40 0 40 0 0 80 0 80 -40 0 -40 0 0 80 0 80 -40 0 -40 0 0 40 0 40 -360 0 -360 0 0 -40z M2640 2680 l0 -40 -40 0 -40 0 0 -40 0 -40 -40 0 -40 0 0 -40 0 -40 -40 0 -40 0 0 -40 0 -40 -40 0 -40 0 0 -40 0 -40 -40 0 -40 0 0 -40 0 -40 -40 0 -40 0 0 -40 0 -40 -40 0 -40 0 0 -40 0 -40 -40 0 -40 0 0 -40 0 -40 -40 0 -40 0 0 -40 0 -40 -40 0 -40 0 0 -40 0 -40 -40 0 -40 0 0 -40 0 -40 -40 0 -40 0 0 -40 0 -40 -40 0 -40 0 0 -40 0 -40 -40 0 -40 0 0 -40 0 -40 -40 0 -40 0 0 -40 0 -40 -40 0 -40 0 0 -40 0 -40 -40 0 -40 0 0 -40 0 -40 -40 0 -40 0 0 -40 0 -40 -40 0 -40 0 0 -40 0 -40 -40 0 -40 0 0 -40 0 -40 -40 0 -40 0 0 -40 0 -40 -40 0 -40 0 0 -40 0 -40 -40 0 -40 0 0 -40 0 -40 -40 0 -40 0 0 -40 0 -40 -40 0 -40 0 0 -40 0 -40 -40 0 -40 0 0 -40 0 -40 -40 0 -40 0 0 -40 0 -40 -40 0 -40 0 0 -40 0 -40 -40 0 -40 0 0 -40 0 -40 -40 0 -40 0 0 -40 0 -40 -40 0 -40 0 0 -40 0 -40 -40 0 -40 0 0 -40 0 -40 -40 0 -40 0 0 -40 0 -40 80 0 80 0 0 40 0 40 40 0 40 0 0 40 0 40 40 0 40 0 0 40 0 40 40 0 40 0 0 40 0 40 40 0 40 0 0 40 0 40 40 0 40 0 0 40 0 40 40 0 40 0 0 40 0 40 40 0 40 0 0 40 0 40 40 0 40 0 0 40 0 40 40 0 40 0 0 40 0 40 40 0 40 0 0 40 0 40 40 0 40 0 0 40 0 40 40 0 40 0 0 40 0 40 40 0 40 0 0 40 0 40 40 0 40 0 0 40 0 40 40 0 40 0 0 40 0 40 40 0 40 0 0 40 0 40 40 0 40 0 0 40 0 40 40 0 40 0 0 40 0 40 40 0 40 0 0 40 0 40 40 0 40 0 0 40 0 40 40 0 40 0 0 40 0 40 40 0 40 0 0 40 0 40 40 0 40 0 0 40 0 40 40 0 40 0 0 40 0 40 40 0 40 0 0 40 0 40 40 0 40 0 0 40 0 40 40 0 40 0 0 40 0 40 40 0 40 0 0 40 0 40 40 0 40 0 0 40 0 40 40 0 40 0 0 40 0 40 40 0 40 0 0 40 0 40 40 0 40 0 0 80 0 80 -40 0 -40 0 0 -40z M2080 1240 l0 -40 -80 0 -80 0 0 -40 0 -40 -80 0 -80 0 0 -80 0 -80 80 0 80 0 0 80 0 80 80 0 80 0 0 40 0 40 160 0 160 0 0 -40 0 -40 80 0 80 0 0 -200 0 -200 -80 0 -80 0 0 -40 0 -40 -80 0 -80 0 0 -40 0 -40 -80 0 -80 0 0 -40 0 -40 -80 0 -80 0 0 -40 0 -40 -40 0 -40 0 0 -40 0 -40 -40 0 -40 0 0 -160 0 -160 480 0 480 0 0 40 0 40 -400 0 -400 0 0 120 0 120 40 0 40 0 0 40 0 40 40 0 40 0 0 40 0 40 80 0 80 0 0 40 0 40 80 0 80 0 0 40 0 40 80 0 80 0 0 40 0 40 80 0 80 0 0 200 0 200 -80 0 -80 0 0 40 0 40 -80 0 -80 0 0 40 0 40 -160 0 -160 0 0 -40z"/></g></svg> ; combined natural abundance 100%; *g*_1_ = 2.197, *g*_2_ = *g*_3_ = 2.054)17*d* the metal coupling constant *A*_1_ [*A*_1_(^197^Au) = 43 G; *A*_1_(^63/65^Cu) = 197 G (ref. 17*d*)] is significantly reduced in **Au(TPP)**. This suggests a more covalent character of the Au^II^–N bonds compared to the Cu^II^–N bonds in their respective TPP^2–^ complexes. For [C^+^]* with strongly electron donating *meso* substituents at the gold porphyrin a much larger hfc to ^197^Au has been reported [*A*_1_(^197^Au) = 180 G].13*d* In accordance with the stronger nephelauxetic effect of porphyrins, complex **D^2+^** with the pure σ donor ligand ethylenediamine features a significantly larger hyperfine coupling to ^197^Au than **Au(TPP)** as well.^15^

**Table 2:** X-band EPR data of one-electron reduced porphyrinato gold(iii) complexes obtained from simulations of the experimental spectra

	Major species					Minor species		
	*g*_1,2,3_	*A*(^197^Au)_1,2,3_/G	4 × *A*(^14^N)_1,2,3_/G	Line width (Gauss/Lorentz)	Fraction/%	*g*_1,2,3_	4 × *A*(^14^N)_1,2,3_/G	Line width (Gauss/Lorentz)
**Au(TPP)**	2.182, 2.056, 1.982	43, 20, 20	18, 22, 21	1.0/0.3	100	—	—	—
**1a**	2.190, 2.056, 1.974	44, 29, 29	18, 22, 21	1.7/0.3	78	2.016, 2.005, 1.995^*a*^	1, 12, 1	0.75/0.1
**1a/TBACl**	2.190, 2.056, 1.974	44, 29, 29	18, 22, 21	1.7/0.3	65	2.016, 2.005, 1.994	1, 12, 1	0.9/0.3
**2a**	2.192, 2.062, 1.963	46, 25, 25	18, 22, 22	1.7/0.3	94	2.018, 2.005, 1.994	1, 12, 1	0.9/0.2
**3a**	2.192, 2.062, 1.968	44, 29, 29	18, 22, 21	1.7/0.3	95	2.016, 2.005, 1.994	1, 13, 1	0.9/0.3
**4a**	2.175, 2.057, 1.973	43, 29, 29	18, 22, 21	1.7/0.3	100	—	—	—
**4b**	2.175, 2.056, 1.972	42, 25, 25	18, 22, 21	3.0/0.3	100	—	—	—
**4c**	2.175, 2.055, 1.974	44, 29, 29	18, 22, 21	1.7/0.3	100	—	—	—

^*a*^The nitro radical **1a′′** (23%) shows *g*_1,2,3_ = 2.031, 2.005, 1.948 and 4 × *A*(^14^N)_1,2,3_ = 2, 17, 2 G.

**Fig. 5:**
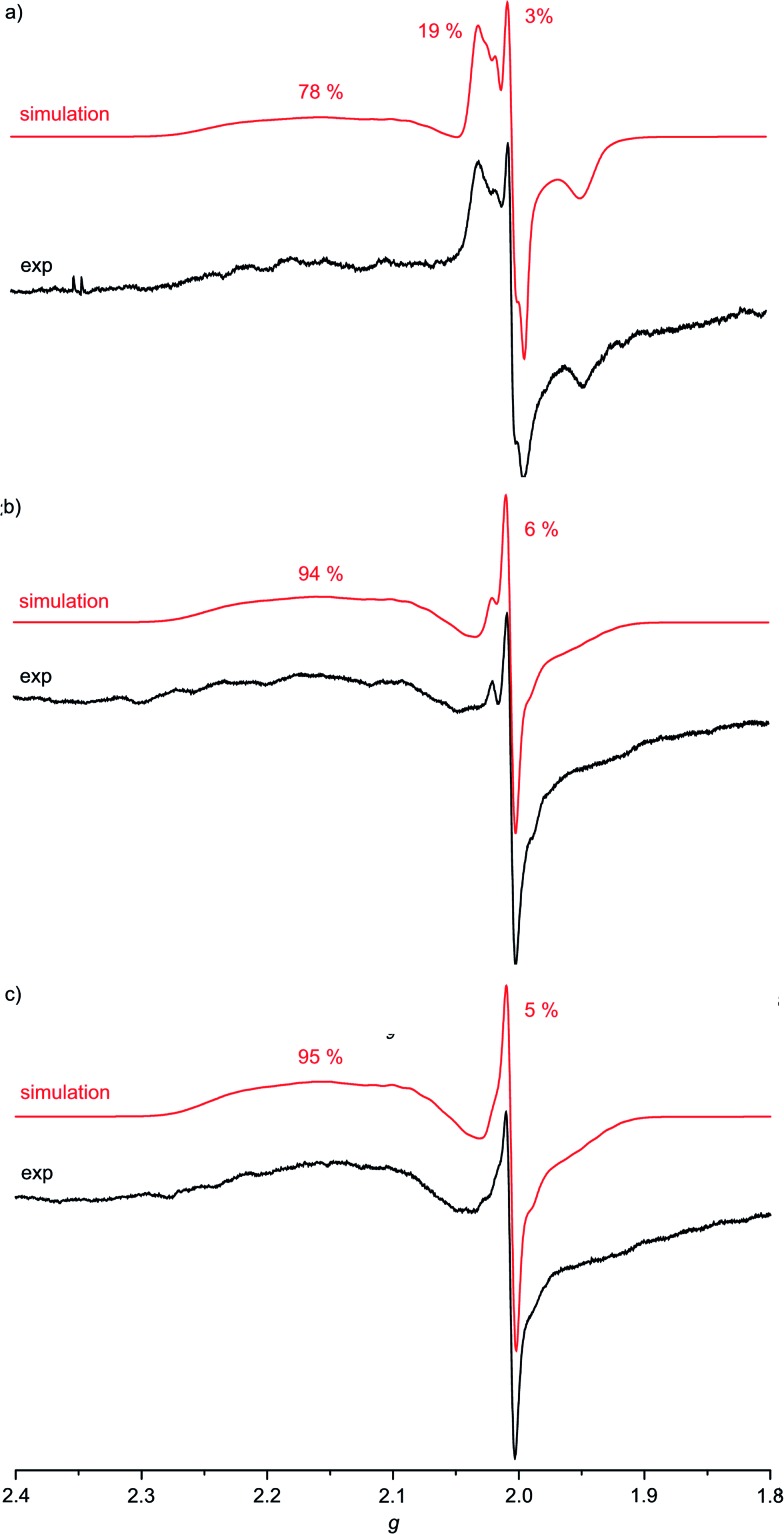
X-band EPR spectra of (a) **1a**/**1a′**/**1a′′** (78 : 3 : 19), (b) **2a**/**2a′** (94 : 6) and (c) **3a**/**3a′** (95 : 5) in frozen CH_2_Cl_2_ solution (77 K, 9.4 GHz) and corresponding simulations.

**Fig. 6:**
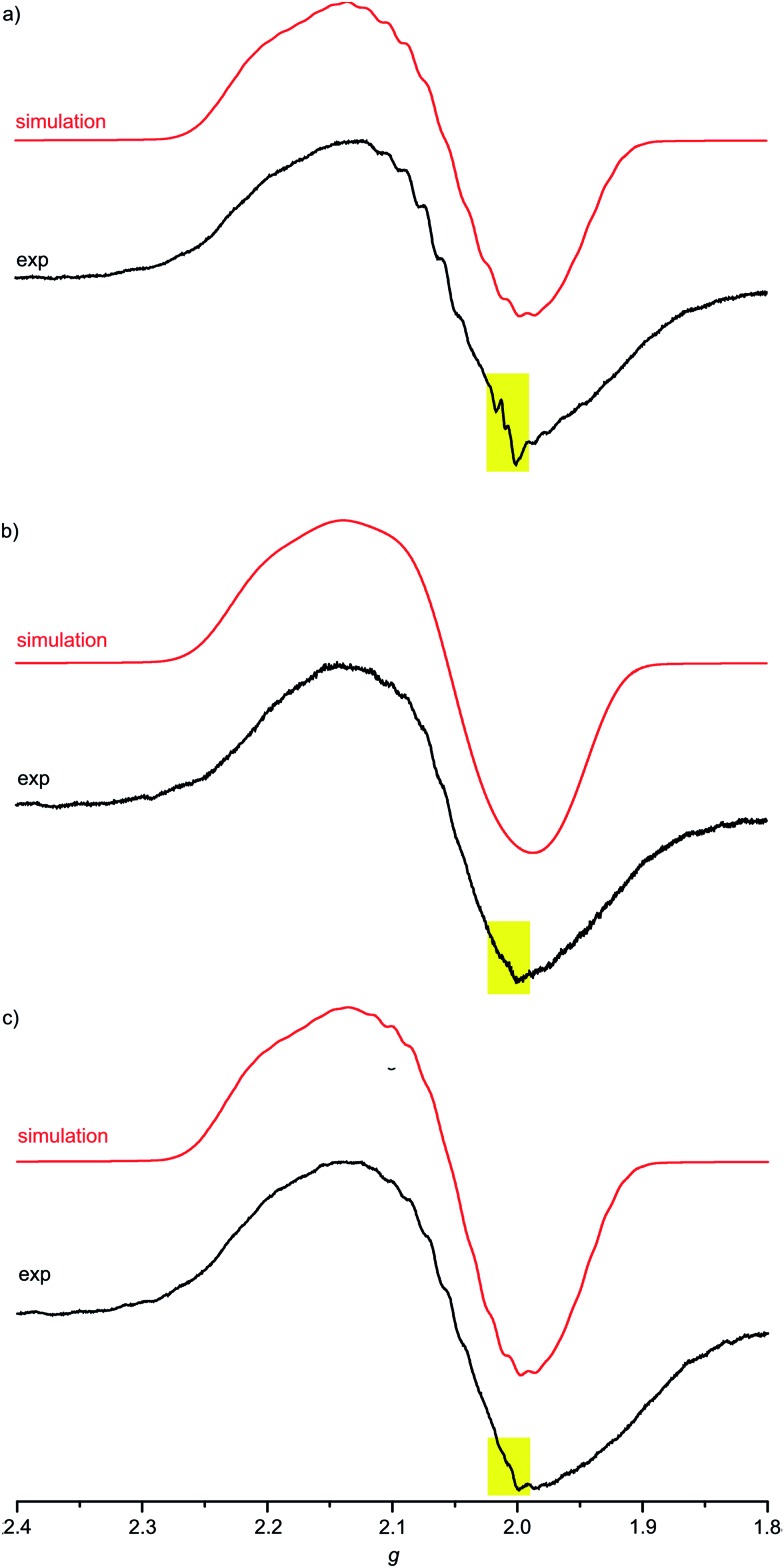
X-band EPR spectra of (a) **4a** (MeOH), (b) **4b** (THF) and (c) **4c** (THF) in frozen solution (77 K, 9.4 GHz) and corresponding simulations; yellow squares highlight the presence of traces of porphyrin radical anions **4a′**, **4b′** and **4c′**, respectively.

For complexes **1a–3a** the broad EPR resonance corresponding to the Au^II^ valence isomer is less well resolved due to the lower symmetry and hence different superhyperfine interactions (Fig. 5). Furthermore, the broad Au^II^ resonance is superimposed by a sharp slightly rhombic resonance around *g* = 2.0. For **2a** and **3a**, this sharp resonance accounts for approximately 5–6% of the total EPR intensity. The pattern can be satisfactorily simulated by *g*_1,2,3_ = 2.018, 2.005, 1.994 and hyperfine coupling to four nitrogen atoms (*A*_1,2,3_ = 1, 12, 1 G). These data fit to gold(iii) porphyrin radical anions **2a′** and **3a′**. For **4a–4c** prepared in THF or MeOH, the corresponding gold(iii) porphyrin radical anions **4a′**, **4b′** and **4c′** are only present in negligible amounts (Fig. 6). Hence, in all these cases the equilibrium between the gold(ii) valence isomers **2a–4c** and their corresponding porphyrin radical anions **2a′–4c′** is in favour of the gold(ii) isomers. The very strong preference of **4a–4c** over **4a′–4c′** independent of the *meso* substituents might be due to a solvent effect overwhelming the substituent effects. Indeed, in THF or in MeOH solvent-separated ion-pairs **[4a–4c]^+^//[PF_6_]^–^** should be present while in CH_2_Cl_2_ solution contact ion pairs of **[2a,3a][PF_6_]** are formed. Indeed, reduction of **[2a][PF_6_]** or **[3a][PF_6_]** in THF resulted in EPR spectra mainly displaying the gold(ii) valence isomers (ESI, Fig. S29 and S30†). The counterion location might affect the charge and spin distribution in the neutral species as well (*vide infra*).

The much more intense sharp EPR resonance present in the EPR spectrum of **1a** obtained from **[1a][PF_6_]** in CH_2_Cl_2_ (Fig. 5a) differs from the sharp resonances assigned to the porphyrin π radical anions **2a′** and **3a′**. Indeed, simulations of the resonance suggests the presence of a further radical species with *g*_1,2,3_ = 2.031, 2.005, 1.948 and hyperfine coupling to a single nitrogen atom (*A*_1,2,3_ = 2, 17, 2 G). This is in good accordance with a nitroarene radical anion.^23^ Hence, this distinct EPR resonance is assigned to a nitrophenyl radical anion valence isomer **1a′′**. The radical distribution **1a** : **1a′** : **1a′′** is estimated as 78 : 3 : 19. The decomposition into the component spectra is displayed in the ESI.† The effect of the type of counterions was probed by adding two equivalents of [^*n*^Bu_4_N]Cl to the solution prior to reduction of the gold(iii) porphyrin with CoCp_2_. No significant changes are observed for **Au(TPP)**, **3a** (CH_2_Cl_2_) or **4c** (THF) in the presence of chloride. However, the presence of chloride transforms the **1a** : **1a′** : **1a′′** radical mixture almost completely into a **1a** : **1a′** mixture (65 : 35) as only the gold(ii) resonance and the porphyrin radical anion resonance are observed under these conditions (ESI,† Table 2), similar to the **2a′** and **3a′** cases. Hence, for the nitro derivative **1a**, three possible valence isomers are possible: the gold(ii) radical (**1a**), the porphyrin based π radical (**1a′**) and a further nitro group based π radical (**1a′′**). Assuming, that rapid freezing does not strongly affect the equilibria of valence isomers, we can conclude that the environment, namely anions and the solvent, appears to influence these valence isomeric equilibria significantly. The substituents influence the equilibria as well, especially, when a strongly electron accepting nitro group is present. A conceivable intervalence transition between **1a** and **1a′**/**1a′′** is not detected in the UV/Vis spectrum by comparison with the spectra of **2a** and **3a** (Fig. 3). This might be associated with the different orbital symmetries of **1a** and **1a′**/**1a′′**.

The electronic structure of the gold(ii) radicals **1a–4c**, the valence isomeric equilibrium **1a**/**1a′′** and the effect of counterions will be addressed by theoretical methods in the next section.

### DFT studies of (porphyrinato)gold(iii) complexes (series **[1a]^+^–[3a]^+^** and series **[4a]^+^–[4c]^+^**) and the corresponding reduced species (series **1a–3a** and series **4a–4c**)

**Fig. 7:**
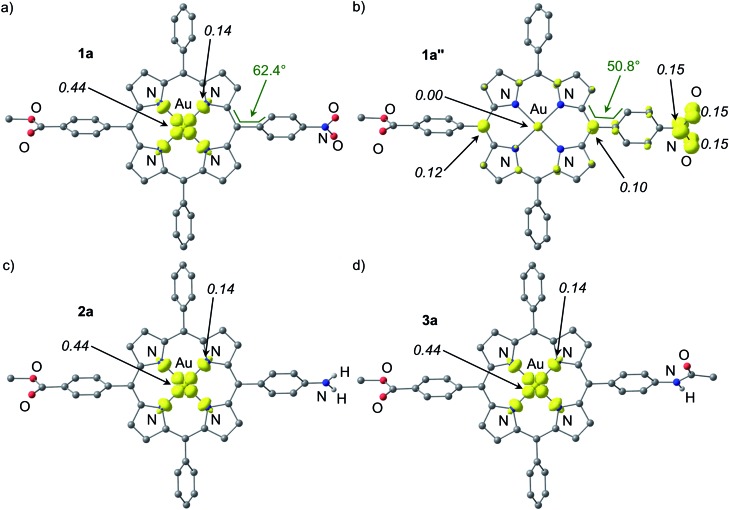
Spin densities of DFT optimised geometries of (a) **1a**, (b) **1a′′** (constrained), (c) **2a** and (d) **3a** (isosurface value at 0.01 a.u. in yellow; 0.006 a.u. for **1a′′**; UB3LYP, LANL2DZ, IEFPCM CH_2_Cl_2_; CH hydrogen atoms omitted; Mulliken spin densities in italics).

Both the geometries of the cationic gold(iii) porphyrins **[Au(TPP)]^+^**, **[1a]^+^–[4c]^+^** and the structures of all corresponding neutral species **Au(TPP)**, **1a–3a** and **4a–4c** were optimised by DFT methods (B3LYP, LANL2DZ, IEFPCM CH_2_Cl_2_; Table 3, Fig. 7 and ESI†). The most significant differences between the cationic gold(iii) complexes and their neutral congeners are found in the Au–N distances which increase by *ca.* 4% from 2.051 to 2.124 Å in all cases (Table 3). The large changes of the Au–N distances (Table 3) contribute to the reorganisation energy of the reduction process.13*c* The gold ions are located nearly perfectly in the centre of the four pyrrolic nitrogen atoms in all complexes. The macrocycle itself displays only minor distortions both in the cations as well as in the neutral complexes. A minor increase of the saddling (*B*_2u_) distortion is noted in the neutral complexes (Table 3). These metrical data of **1a–4c** strongly suggest that the reduction of the metal centre to Au^II^ is favoured in the electronic ground state. A reduction of the porphyrin to its radical anion **1a′–4c′** should result in pronounced macrocycle distortions as well as in small Au^III^–N (radical anion) bond distances which is not observed. The calculated Mulliken spin densities are in full accordance with these structural parameters. In all neutral complexes the majority of the spin density is located at the metal centre (Mulliken spin density at Au: 0.44), especially in the 5d_*x*^2^–*y*^2^_ orbital (Fig. 7 and ESI†). The remainder is distributed over the pyrrolic nitrogen atoms in the σ-orbitals pointing towards the metal centre (Mulliken spin density at N: 0.14). This clearly advocates a gold-centred radical localised in the σ-system of the almost planar molecule rather than a porphyrin radical anion with the spin delocalised in the π-system of the porphyrin. The DFT determined Au^II^ electronic ground states of **1a–4c** perfectly match the experimentally derived ground states. The spin densities are also in full agreement with experimentally determined EPR parameters (*g* values, ^197^Au hyperfine coupling and ^14^N superhyperfine coupling). Compared with the isoelectronic **Cu(TPP)** [Mulliken spin density at Cu: 0.58; Mulliken spin density at N: 0.105] the spin densities are more delocalised onto the nitrogen atoms which is in agreement with the EPR results as well (ESI†).17*d*

**Table 3:** Metrical data of DFT optimised porphyrinato gold(iii) complexes and their one-electron reduced counterparts

	Au–N/Å	Centre–N/Å^*a*^	C_α_–N–N′–C′_α_ (ruffling, *B*_1u_)/°	N–centre–N′ (saddling, *B*_2u_)^*a*^/°	N–O/Å	Torsion angle with respect to porphyrin plane C5–C12–C38–C43	Au···F/Å
**[Au(TPP)]^+^**	2.051/2.051/2.051/2.051	2.051/2.051/2.051/2.051	–0.49 to +0.50	179.51/179.51			
**Au(TPP)**	2.124/2.124/2.124/2.124	2.124/2.124/2.124/2.124	–0.05 to +0.04	178.46/178.46			
**[1a]^+^**	2.050/2.050/2.050/2.050	2.050/2.050/2.050/2.050	–2.23 to +1.95	179.57/179.57	1.281/1.281	66.1	
**1a**	2.124/2.124/2.124/2.124	2.124/2.124/2.124/2.124	–0.27 to –0.05	178.38/178.38	1.283/1.283	62.4	
**1a′′**	2.057/2.058/2.058/2.057	2.057/2.058/2.058/2.057	–1.39 to +1.09	179.25/179.25	1.349/1.349^*b*^	50.8	
**[1a···PF_6_]**	2.047/2.050/2.052/2.051	2.048/2.050/2.050/2.050	–2.71 to +1.47	179.82/179.83	1.282/1.282	68.3	3.146
**[1a··· PF_6_]^–^**	2.127/2.119/2.127/2.124	2.126/2.121/2.126/2.122	–1.54 to +1.78	178.75/178.75	1.284/1.285	61.4	4.076
**[1a′′···PF_6_]^–^**	2.057/2.057/2.056/2.055	2.056/2.056/2.057/2.056	–6.18 to +6.03	179.35/179.35	1.315/1.316	50.2	3.249
**[2a]^+^**	2.051/2.052/2.051/2.052	2.052/2.052/2.052/2.051	–3.54 to +3.44	179.60/179.60			
**2a**	2.124/2.125/2.125/2.125	2.125/2.125/2.125/2.125	–2.26 to +2.35	178.28/178.28			
**[3a]^+^**	2.051/2.051/2.051/2.051	2.051/2.051/2.051/2.051	–0.39 to +0.63	179.55/179.55			
**3a**	2.124/2.124/2.124/2.124	2.124/2.124/2.124/2.124	–0.85 to +0.69	178.43/178.43			
**[4a]^+^**	2.051/2.051/2.051/2.051	2.051/2.051/2.051/2.051	–1.05 to +1.19	179.58/179.58			
**4a**	2.124/2.124/2.124/2.124	2.124/2.124/2.124/2.124	–0.76 to +0.74	178.42/178.42			
**[4b]^+^**	2.052/2.052/2.052/2.052	2.052/2.052/2.052/2.052	–1.03 to +0.90	179.56/179.56			
**4b**	2.125/2.125/2.125/2.125	2.125/2.125/2.125/2.125	–0.77 to +0.69	178.35/178.35			
**[4c]^+^**	2.051/2.051/2.051/2.051	2.051/2.051/2.051/2.051	–0.49 to +0.89	179.56/179.56			
**4c**	2.124/2.124/2.124/2.124	2.124/2.124/2.124/2.124	–0.92 to +1.10	178.53/178.53			

^*a*^Centre denotes the geometric centre of the four pyrrole nitrogen atoms.

^*b*^Constrained distances.

The special case of the nitro derivative **1a** which displays significant amounts of the nitrobenzene π radical anion valence isomer **1a′′** in the EPR spectrum (Fig. 5a) was treated by DFT methods as well. However, all geometry optimisation attempts (with the employed functional, basis set and tight convergence criteria) converged to the stable Au^II^ valence isomer **1a**. In order to get an impression on the spin density distribution in valence isomer **1a′′**, the nitrobenzene radical anion [C_6_H_5_NO_2_]˙^–^ was separately optimised by DFT methods giving NO distances of 1.349 Å. These NO distances were then constrained to 1.349 Å in geometry optimizations of **1a′′** giving the (constrained) optimised structure of **1a′′** as shown in Fig. 7. The Au–N bond lengths of **1a′′** are fully consistent with a gold(iii) oxidation state (Table 3). Compared to **[1a]^+^** and **1a** the C_6_H_4_NO_2_ torsion angle with respect to the porphyrin plane C5–C12–C38–C43 is significantly reduced from 66.1° and 62.4° to 50.8° suggesting a conjugative electron withdrawing effect of the gold(iii) porphyrin as expected for a π-centred radical. The spin density is mainly located at the NO_2_ substituent and partially delocalised over the π-system of the porphyrin. The Mulliken spin density at the gold atom in **1a′′** is essentially zero (Fig. 7).

**Fig. 8:**
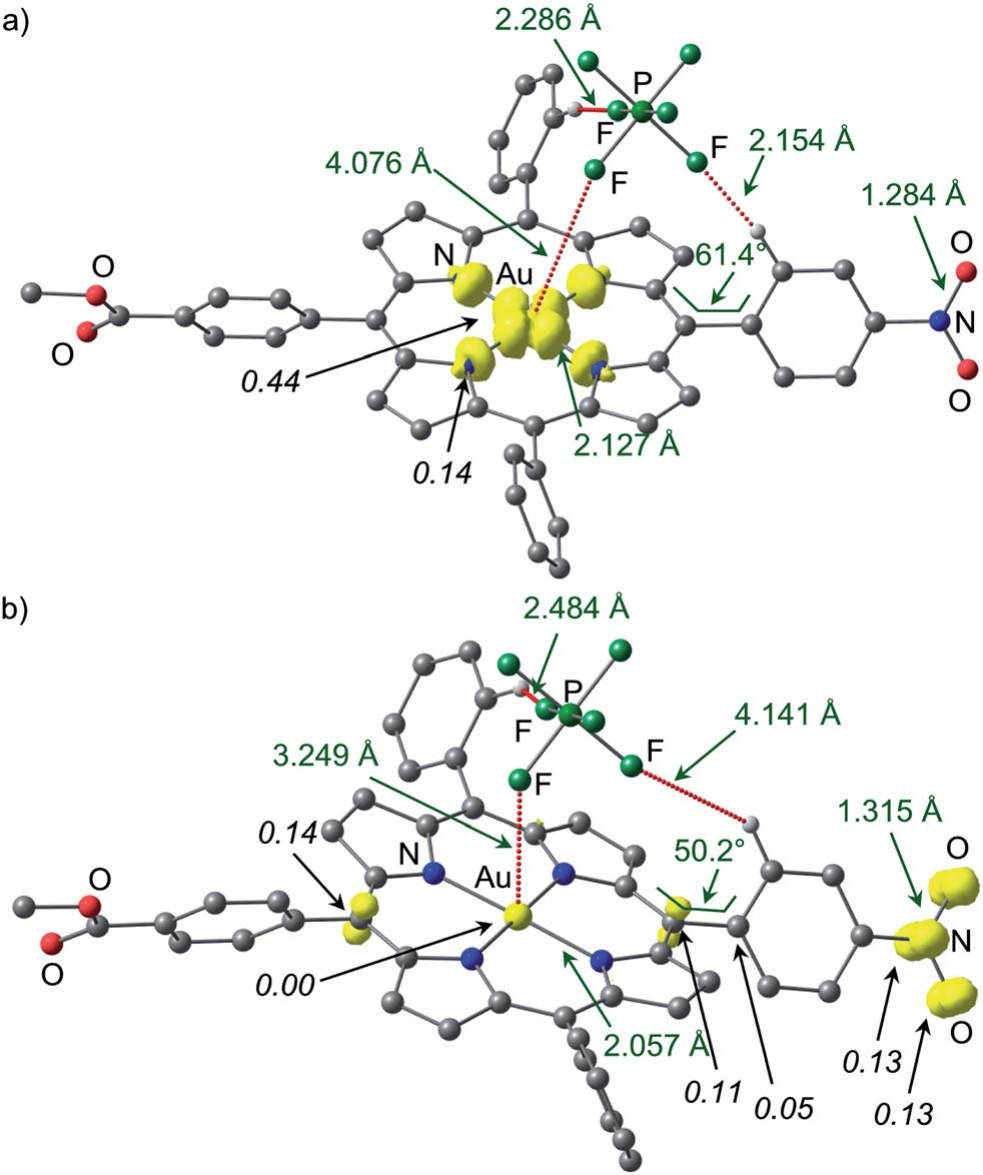
Spin densities of DFT optimised geometries of (a) **[1a···PF_6_]^–^** and (b) **[1a′′···PF_6_]^–^** (isosurface value at 0.01 a.u. in yellow; UB3LYP, LANL2DZ, IEFPCM CH_2_Cl_2_; hydrogen atoms which are not involved in hydrogen bonds are omitted; Mulliken spin densities in italics).

As an unconstrained optimization of **1a′′** was unsuccessful, we investigated the effect of the counterion [PF_6_]^–^ on the charge and spin distribution in **[1a···PF_6_]^–^** and **[1a′′···PF_6_]^–^**, respectively. Indeed, we succeeded in optimising both valence isomers **[1a···PF_6_]^–^** and **[1a′′···PF_6_]^–^** without any constraints (Fig. 8). The Au^II^ valence isomer **[1a···PF_6_]^–^** is preferred by 12 kJ mol^–1^. In this Au^II^ isomer **[1a···PF_6_]^–^** the [PF_6_]^–^ ion is not coordinated to the metal (Au···F 4.076 Å) but only hydrogen-bonded to two CH groups of the aryl substituents (Fig. 8a). The spin density is again localised at the metal centre (Mulliken spin density at Au: 0.44) and the pyrrolic nitrogen atoms (Mulliken spin density at N: 0.14). In the nitro-based π radical **[1a′′···PF_6_]^–^** the [PF_6_]^–^ ion is much closer to the gold centre (Au···F 3.249 Å, Fig. 8b). The presence of the negative charge close to the metal centre stabilises the Au^III^ oxidation state and indeed the gold ion carries no spin density. Au–N distances (2.057 Å; **[1a′′···PF_6_]**^–^) fully agree with a gold(iii) porphyrin but not with a gold(ii) porphyrin (2.127 Å; **[1a···PF_6_]**^–^). The N–O distances have increased from 1.284 Å in **[1a···PF_6_]**^–^ to 1.315 Å in **[1a′′···PF_6_]^–^** as expected for population of N–O antibonding orbitals. The spin density is largely confined to the NO_2_ substituent and partially delocalized to the π-system of the porphyrin. The C5–C12–C38–C43 torsion angle of the nitrophenyl substituent decreases from 61.4° (**[1a···PF_6_]^–^**) to 50.2° (**[1a′′···PF_6_]^–^**) similar to the **1a**/**1a′′** (constrained) pair. In essence, the intramolecular electron transfer pathway between **[1a···PF_6_]^–^** and **[1a′′···PF_6_]^–^** encompasses the Au–N and Au···F distances (totally symmetric stretching vibration of the gold coordination sphere), the symmetric NO_2_ stretching mode and a phenyl torsional motion (Fig. 8).

**Scheme 3:**
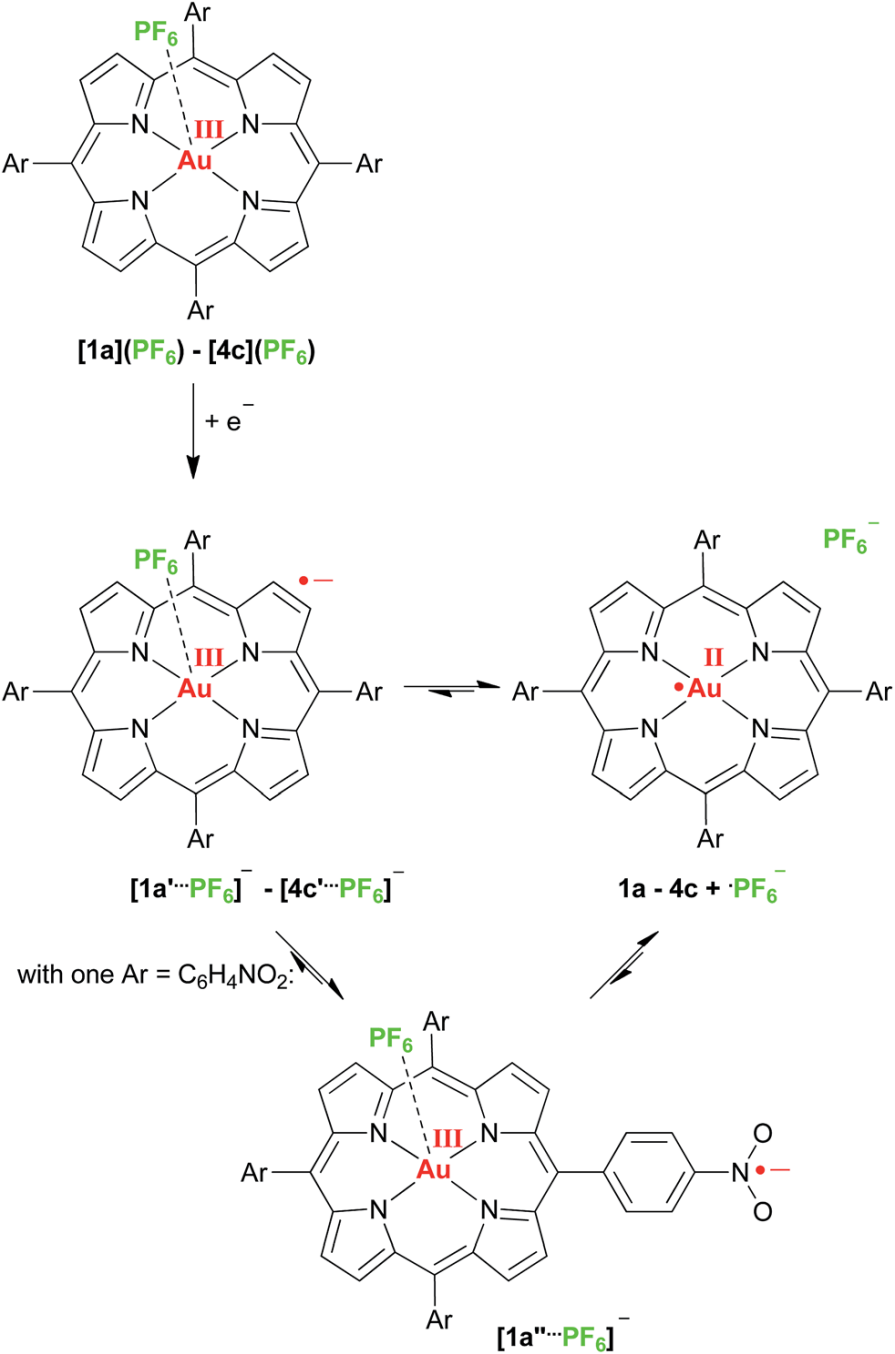
Valence tautomeric equilibria of gold(ii) porphyrins and gold(iii) porphyrin radical anions.

With respect to photoinduced electron transfer reactions using porphyrinato gold(iii) complexes as electron acceptors we suggest that the initial kinetic reduction product of a porphyrinato gold(iii) complex should be a gold(iii) porphyrin π radical anion (such as **1a′–4c′**) due to the smaller activation barrier and the better electronic coupling to electron donors (Scheme 3). In a following intramolecular valence isomerisation the electron shifts to the central gold ion (σ-system) with concomitant dissociation of the counterion giving the thermodynamic Au^II^ product (such as **1a–4c**) (Scheme 3). The latter chemical reaction will render the whole photoinduced ET process irreversible, which is advantageous for further reactivity of the redox sites. In the case of nitro substituted porphyrins a further valence isomer **[1a′′···PF_6_]^–^** with a nitrophenyl π radical anion is existent as well. Both the solvent, the present ions and the substituents determine the final charge and spin distribution.

## Experimental

Porphyrins H_2_TPP, **Ia**, **IIa**, **IIIa**, **IVa**, **IVb** and **IVc** were prepared according to published procedures.^17^ Solvents were dried by standard methods. Other reagents were used as received from commercial suppliers (Acros, Sigma-Aldrich). NMR spectra were recorded on a Bruker Avance DRX 400 spectrometer at 400.31 MHz (^1^H), 100.05 MHz (^13^C{^1^H}), 162.05 MHz (^31^P{^1^H}). Resonances are reported in ppm *versus* the solvent signal as an internal standard. CD_2_Cl_2_ (^1^H: *δ* = 5.32 ppm; ^13^C: *δ* = 53.8 ppm), d_8_-THF (^1^H: *δ* = 1.73, 3.58 ppm; ^13^C: *δ* = 25.5, 67.7 ppm) MeOD (^1^H: *δ* = 3.31, 4.87 ppm; ^13^C: *δ* = 49.0 ppm) and *versus* external H_3_PO_4_ (85%) (^31^P: *δ* = 0 ppm); (s) = singlet, (pt) = pseudo triplet (unresolved doublet of doublets), (br, s) = broad singlet, septet (sept). IR spectra were recorded with a BioRad Excalibur FTS 3100 spectrometer as KBr disks; (vs) = very strong, (s) = strong, (m) = medium, (w) = weak. ESI mass spectra were recorded on a Micromass Q-TOF-Ultima spectrometer. Electrochemical experiments were carried out on a BioLogic SP-50 voltammetric analyzer using a glassy carbon working electrode, a platinum wire as the counter electrode and a 0.01 M Ag/AgNO_3_ electrode as the reference electrode. The measurements were carried out at a scan rate of 100 mV s^–1^ for cyclic voltammetry experiments and for square wave voltammetry experiments using a concentration of 10^–3^ M in 0.1 M [^*n*^Bu_4_N][PF_6_] as the supporting electrolyte in THF (MeOH). Potentials are given relative to the ferrocene/ferrocenium couple. Spectroelectrochemical experiments were performed using a thin layer quartz glass (path length 1 mm) cell kit (GAMEC Analysentechnik, Illingen, Germany) equipped with a Pt gauze working electrode, a Pt counter electrode and a Ag/AgNO_3_ reference electrode (10^–5^ M solutions in THF or in MeOH, containing 0.1 M [^*n*^Bu_4_N][PF_6_]). X-band CW EPR spectra were measured on a Miniscope MS 300 (Magnettech GmbH, Germany). *g*-Values are referenced to external Mn^2+^ in ZnS (*g* = 2.118, 2.066, 2.027, 1.986, 1.946, 1.906). Simulations were performed with the program package EasySpin.^24^ UV/Vis/NIR spectra were recorded on a Varian Cary 5000 spectrometer using 1.0 cm cells (Hellma, suprasil).

### DFT calculations

Density functional calculations were carried out with the Gaussian09/DFT series^25^ of programs. The B3LYP formulation of density functional theory was used employing the LANL2DZ basis set. To include solvent effects the integral equation formalism polarisable continuum model (IEFPCM CH_2_Cl_2_) was employed. No (symmetry) constraints were imposed on the molecules, except for the NO distance constraint for **1a′**. The presence of energy minima of the ground states was checked by analytical frequency calculations.

### EPR measurements of radical anions

Under an inert atmosphere a solution of the respective gold(iii) porphyrin complex (*c* = 5 × 10^–3^ M) in CH_2_Cl_2_ (**[Au(TPP)][PF_6_]**, **[1a][PF_6_]**, **[2a][PF_6_]**, **[3a][PF_6_]**), MeOH (**[4a][PF_6_]**) or THF (**[4b][PF_6_]**, **[4c][PF_6_]**) was treated with 0.95 equivalents of cobaltocene CoCp_2_. The X-band EPR spectrum of the sample was measured immediately after freezing the solution to 77 K. The effect of chloride was measured by addition of 2.0 equivalents of [^*n*^Bu_4_N]Cl prior to the reduction.

### [5,10,15,20-Tetraphenylporphyrinato]gold(iii) hexafluorophosphate **[Au(TPP)][PF_6_]**

5,10,15,20-Tetraphenylporphyrin (50 mg, 0.08 mmol), potassium tetrachloridoaurate(iii) (121 mg, 0.32 mmol) and sodium acetate (302 mg, 3.68 mmol) were dissolved in glacial acetic acid (20 mL). The reaction mixture was heated to reflux for 20 h, allowed to cool to room temperature, and diluted with dichloromethane (100 mL). The mixture was washed with water (2 × 50 mL), saturated sodium carbonate solution (2 × 50 mL) and water (1 × 50 mL), dried over anhydrous magnesium sulfate and filtered. The filtrate was evaporated to dryness and the residue dissolved in dichloromethane (50 mL). The organic phase was stirred with a saturated aqueous solution of potassium hexafluorophosphate (10 mL) for 72 h. The mixture was diluted with dichloromethane (100 mL) and washed with water (2 × 50 mL), dried over anhydrous magnesium sulfate, and filtered. The filtrate was removed under reduced pressure and the residue purified by chromatography over silica [dichloromethane : methanol (20 : 1); *R*_f_ = 0.40] to yield **[Au(TPP)][PF_6_]** (66 mg, 0.09 mmol, 88%) as a purple crystalline solid. C_44_H_28_AuF_6_N_4_P (954.7). UV/Vis: *λ*_max_(THF)/nm 409 (*ε*/M^–1^ cm^–1^ 258 000), 523 (12 400). IR: *ν*_max_/cm^–1^ 1638 (m), 1617 (s), 839 (vs, PF), 556 (m, PF_6,def_). NMR: *δ*_H_ (400 MHz, CD_2_Cl_2_) 7.93 (12H, m, H^5/5/10/10/15/15/20/20^_3/4/3/4/3/4/3/4_), 8.24 (8H, m, H^5/10/15/20^_2/2/2/2_), 9.36 (8H, s, H^2,3,7,8,12,13,17,18^); *δ*_C_ (100 MHz, CD_2_Cl_2_) 124.1 (s, C^5/10/15/20^), 128.1 (s, C^5/10/15/20^_3/3/3/3_), 129.8 (s, C^5/10/15/20^_4/4/4/4_), 132.7 (s, C^2/3/7/8/12/13/17/18^), 134.5 (s, C^5/10/15/20^_2/2/2/2_), 137.3 (s, C^1/4/6/9/11/14/16/19^), 138.7 (s, C^5/10/15/20^_1/1/1/1_). *δ*_P_ (162 MHz, CD_2_Cl_2_) –143.8 (sept, ^1^*J*_PF_ = 710 Hz). MS (ESI): *m*/*z* 809.99 (100%) [M]^+^. HR-MS (ESI): *m*/*z* 809.1993 (calcd for C_44_H_28_AuN_4_: 809.1980). CV (Fc/Fc^+^, THF): *E*_½_/V –2.350, –1.650, –0.975.

### [10,20-Di(phenyl)-15-(4-(methoxycarbonylphenyl))-5-(4-nitrophenyl)porphyrinato]gold(iii) hexafluorophosphate **[1a][PF_6_]**

10,20-Di(phenyl)-15-(4-(methoxycarbonylphenyl))-5-(4-nitrophenyl)porphyrin **Ia** (100 mg, 0.14 mmol), potassium tetrachloridoaurate(iii) (212 mg, 0.56 mmol) and sodium acetate (529 mg, 6.44 mmol) were dissolved in glacial acetic acid (40 mL). The reaction mixture was heated to reflux for 22 h, allowed to cool to room temperature, and diluted with dichloromethane (200 mL). The mixture was washed with water (2 × 100 mL), saturated aqueous sodium carbonate solution (2 × 100 mL) and water (1 × 100 mL), dried over anhydrous magnesium sulfate and filtered. The filtrate was evaporated to dryness and the residue dissolved in dichloromethane (100 mL). The organic phase was stirred with a saturated aqueous solution of potassium hexafluorophosphate (20 mL) for 72 h. The mixture was diluted with dichloromethane (100 mL) and washed with water (2 × 50 mL), dried over anhydrous magnesium sulfate, and filtered. The filtrate was removed under reduced pressure and the residue purified by chromatography over silica [dichloromethane : methanol (10 : 1); *R*_f_ = 0.35] to yield **[1a][PF_6_]** (64 mg, 0.06 mmol, 43%) as a purple crystalline solid. C_46_H_29_AuF_6_N_5_O_4_P (1057.7). UV/Vis: *λ*_max_(THF)/nm 410 (*ε*/M^–1^ cm^–1^ 329 000), 523 (17 600). IR: *ν*_max_/cm^–1^ 1717 (s, CO_ester_), 1597 (m), 1520 (s, NO_asym_), 1439 (m), 1346 (s, NO_sym_), 1277 (s, OCO_def_), 1106 (m), 1034 (s), 1018 (s), 837 (vs, PF), 556 (s, PF_6,def_). NMR: *δ*_H_ (400 MHz, CD_2_Cl_2_) 4.12 (3H, s, H^15^_6_), 7.95 (6H, m, H^10/10/20/20^_3/3/4/4_), 8.29 (4H, d, H_2/2_^10/20^), 8.39 (2H, d, ^3^*J*_HH_ = 8.2 Hz, H^15^_2_), 8.50 (2H, d, ^3^*J*_HH_ = 8.5 Hz, H^5^_2_), 8.57 (2H, d, ^3^*J*_HH_ = 8.1 Hz, H^15^_3_), 8.75 (2H, d, ^3^*J*_HH_ = 8.5 Hz, H^5^_3_), 9.35 (8H, m, H^2,3,7,8,12,13,17,18^); *δ*_C_ (100 MHz, CD_2_Cl_2_) 52.5 (s, C^15^_6_), 121.4 (s, C^15^), 123.4 (s, C^5^_3_), 124.8 (s, C^10/20^), 128.4 (s, C^10/20^_3/3_), 129.4 (s, C^15^_3_), 130.0 (s, C^10/20^_4/4_), 132.2 (s, C^15^_4_), 132.8–133.7 (multiple s, C^2/3/7/8/12/13/17/18^), 134.8 (s, C^10/20/15^_2/2/2_), 136.5 (s, C^5^_2_), 136.9–138.0 (multiple s, C^1/4/6/9/11/14/16/19^), 138.9 (s, C^10/20^_1/1_), 143.3 (s, C^15^_1_), 145.4 (s, C^5^_1_), 149.4 (s, C^5^_4_), 167.1 (s, C^15^_5_). *δ*_P_ (162 MHz, CD_2_Cl_2_) –144.1 (sept, ^1^*J*_PF_ = 711 Hz). MS (ESI): *m*/*z* 912.11 (100%) [M]^+^. HR-MS (ESI): *m*/*z* 912.1905 (calcd for C_46_H_29_AuN_5_O_4_: 912.1885). CV (Fc/Fc^+^, THF): *E*_½_/V –2.300, –1.795, –1.560, –0.920.

### [5-(4-Aminophenyl)-10,20-di(phenyl)-15-(4-(methoxycarbonylphenyl))porphyrinato]gold(iii) hexafluorophosphate **[2a][PF_6_]**

[10,20-Di(phenyl)-15-(4-(methoxycarbonylphenyl))-5-(4-nitrophenyl)porphyrinato]gold(iii) hexafluorophosphate **[1a][PF_6_]** (100 mg, 0.09 mmol) and tin(ii) chloride dihydrate were suspended in concentrated hydrochloric acid (36%, 50 mL). The reaction mixture was stirred in the dark under argon for 20 h. The mixture was diluted with dichloromethane (200 mL), washed with water (2 × 100 mL), saturated sodium carbonate solution (2 × 100 mL), and water (1 × 100 mL), dried over anhydrous magnesium sulfate and filtered. The filtrate was evaporated to dryness and the residue dissolved in dichloromethane (100 mL). The organic phase was stirred with a saturated aqueous solution of potassium hexafluorophosphate (20 mL) for 72 h. The mixture was diluted with dichloromethane (100 mL) and washed with water (2 × 50 mL), dried over anhydrous magnesium sulfate and filtered. The filtrate was removed under reduced pressure and the residue purified by chromatography over silica [dichloromethane : methanol (25 : 1); *R*_f_ = 0.22] to yield **[2a][PF_6_]** (31 mg, 0.03 mmol, 34%) as a reddish-brown solid. C_46_H_31_AuF_6_N_5_O_2_P (1027.7). UV/Vis: *λ*_max_(THF)/nm 406 (*ε*/M^–1^ cm^–1^ 126 000), 529 (10 800), 589 (4800). IR: *ν*_max_/cm^–1^ 1723 (s, CO_ester_), 1638 (s), 1618 (vs, NH_2,def_), 1277 (s, OCO_def_), 966 (s), 835 (vs, PF), 567 (vs), 557 (s, PF_6,def_). NMR: *δ*_H_ (400 MHz, CD_2_Cl_2_) 4.14 (3H, s, H^15^_6_), 4.7 (2H, br s, NH_2_), 7.25 (2H, d,^3^*J*_HH_ = 8.3 Hz, H^5^_3_), 7.95 (6H, m, H^10/10/20/20^_3/4/3/4_), 8.03 (2H, d, ^3^*J*_HH_ = 8.4 Hz, H^5^_2_), 8.27 (4H, m, H^10/20^_2/2_), 8.37 (2H, d, ^3^*J*_HH_ = 8.3 Hz, H^15^_2_), 8.57 (2H, d, ^3^*J*_HH_ = 8.3 Hz, H^15^_3_), 9.40 (8H, m, H^2,3,7,8,12,13,17,18^); *δ*_C_ (100 MHz, CD_2_Cl_2_) 52.2 (s, C^15^_6_), 114.6 (s, C^5^_3_), 122.6 (s, C^15^), 124.4 (s, C^10/20^), 127.5 (s, C^5^), 128.4 (s, C^10/20^_3/3_), 129.4 (s, C^15^_3_), 130.2 (s, C^10/20^_4/4_), 132.3 (s, C^15^_4_), 132.8–133.6 (multiple s, C^2/3/7/8/12/13/17/18^), 134.8 (s, C^10/20/15^_2/2/2_), 135.6 (s, C^5^_1_), 136.5 (s, C^5^_2_), 137.1–137.7 (multiple s, C^1/4/6/9/11/14/16/19^), 139.0 (s, C^10/20^_1/1_), 143.5 (s, C^15^_1_), 149.5 (s, C^5^_4_), 167.2 (s, C^15^_5_). *δ*_P_ (162 MHz, CD_2_Cl_2_) –143.5 (sept, ^1^*J*_PF_ = 710 Hz). MS (ESI): *m*/*z* 882.09 (100%) [M]^+^. HR-MS (ESI): *m*/*z* 882.2163 (calcd for C_46_H_31_AuN_5_O_2_: 882.2143). CV (Fc/Fc^+^, THF): *E*_½_/V –2.500 (irrev.), –2.280, –1.645, –0.990.

### [5-(4-(*N*-Acetylaminophenyl))-10,20-di(phenyl)-15-(4-(methoxycarbonylphenyl))porphyrinato]gold(iii) hexafluorophosphate **[3a][PF_6_]**

5-(4-(*N*-Acetylaminophenyl))-10,20-di(phenyl)-15-(4-(methoxycarbonylphenyl))porphyrin **IIIa** (50 mg, 0.07 mmol), potassium tetrachloridoaurate(iii) (104 mg, 0.28 mmol) and sodium acetate (258 mg, 3.15 mmol) were dissolved in glacial acetic acid (20 mL). The reaction mixture was heated to reflux for 24 h, allowed to cool to room temperature, and diluted with dichloromethane (100 mL). The mixture was washed with water (2 × 50 mL), saturated aqueous sodium carbonate solution (2 × 50 mL) and water (1 × 50 mL), dried over anhydrous magnesium sulfate and filtered. The filtrate was evaporated to dryness and the residue dissolved in dichloromethane (50 mL). The organic phase was stirred with a saturated aqueous solution of potassium hexafluorophosphate (10 mL) for 72 h. The mixture was diluted with dichloromethane (50 mL) and washed with water (2 × 50 mL), dried over anhydrous magnesium sulfate, and filtered. The filtrate was removed under reduced pressure and the residue purified by chromatography over silica [dichloromethane : methanol (10 : 1); *R*_f_ = 0.43] to yield **[3a][PF_6_]** (32 mg, 0.03 mmol, 49%) as a purple crystalline solid. C_48_H_33_AuF_6_N_5_O_3_P (1069.7). UV/Vis: *λ*_max_(THF)/nm 410 (*ε*/M^–1^ cm^–1^ 124 000), 525 (10 500). IR: *ν*_max_/cm^–1^ 2964 (m, NH), 1717 (s, CO_ester_), 1677 (s, CO_amide_), 1616 (m), 1262 (s, OCO_def_), 1096 (s), 1020 (s), 839 (s), 803 (vs, PF), 708 (m), 557 (m, PF_6,def_). NMR: *δ*_H_ (400 MHz, CD_2_Cl_2_) 2.33 (3H, s, H^5^_6_), 4.11 (3H, s, H^15^_6_), 7.93 (6H, m, H^10/10/20/20^_3/4/3/4_), 8.04 (1H, s, NH), 8.12 (2H, d,^3^*J*_HH_ = 8.3 Hz, H^5^_3_), 8.19 (2H, d, ^3^*J*_HH_ = 8.4 Hz, H^5^_2_), 8.24 (4H, m, H^10/20^_2/2_), 8.34 (2H, d, ^3^*J*_HH_ = 8.3 Hz, H^15^_2_), 8.55 (2H, d, ^3^*J*_HH_ = 8.3 Hz, H^15^_3_), 9.37 (8H, m, H^2,3,7,8,12,13,17,18^); *δ*_C_ (100 MHz, CD_2_Cl_2_) 24.9 (s, C^5^_6_), 53.2 (s, C^15^_6_), 119.2 (s, C^5^_3_), 122.6 (s, C^15^), 124.5 (s, C^10/20^), 124.8 (s, C^5^), 128.4 (s, C^10/20^_3/3_), 129.3 (s, C^15^_3_), 130.2 (s, C^10/20^_4/4_), 132.0 (s, C^15^_4_), 132.5–133.3 (multiple s, C^2/3/7/8/12/13/17/18^), 134.8 (s, C^10/20/15^_2/2/2_), 135.4 (s, C^5^_2_), 137.0–137.9 (multiple s, C^1/4/6/9/11/14/16/19^), 139.0 (s, C^10/20^_1/1_), 140.4 (s, C^5^_1_), 141.5 (s, C^5^_4_), 143.4 (s, C^15^_1_), 169.9 (s, C^15^_5_). *δ*_P_ (162 MHz, CD_2_Cl_2_) –143.5 (sept, ^1^*J*_PF_ = 711 Hz). MS (ESI): *m*/*z* 924.01 (100%) [M]^+^. HR-MS (ESI): *m*/*z* 924.2229 (calcd for C_48_H_33_AuN_5_O_3_: 924.2249). CV (Fc/Fc^+^, THF): *E*_½_/V –2.490 (irrev.), –2.300, –1.630, –0.990.

### [5-(4-(*N*-Acetylaminophenyl))-10,20-di(phenyl)-15-(4-(carboxyphenyl))porphyrinato]gold(iii) hexafluorophosphate **[4a][PF_6_]**

5-(4-(*N*-Acetylaminophenyl))-10,20-di(phenyl)-15-(4-(carboxyphenyl))porphyrin **IVa** (50 mg, 0.07 mmol), potassium tetrachloridoaurate(iii) (104 mg, 0.28 mmol) and sodium acetate (258 mg, 3.15 mmol) were dissolved in glacial acetic acid (20 mL). The reaction mixture was heated to reflux for 24 h, allowed to cool to room temperature and diluted with dichloromethane (100 mL). The mixture was washed with water (2 × 50 mL), saturated aqueous sodium carbonate solution (2 × 50 mL) and water (1 × 50 mL), dried over anhydrous magnesium sulfate and filtered. The filtrate was evaporated to dryness and the residue dissolved in dichloromethane (50 mL). The organic phase was stirred with a saturated aqueous solution of potassium hexafluorophosphate (10 mL) for 72 h. The mixture was diluted with dichloromethane (50 mL) and washed with water (2 × 50 mL), dried over anhydrous magnesium sulfate, and filtered. The filtrate was removed under reduced pressure and the residue purified by chromatography over silica [dichloromethane : methanol (10 : 1); *R*_f_ = 0.41] to yield **[4a][PF_6_]** (24 mg, 0.02 mmol, 28%) as a reddish-brown solid. C_47_H_31_AuF_6_N_5_O_3_P (1055.7). UV/Vis: *λ*_max_(MeOH)/nm 408 (*ε*/M^–1^ cm^–1^ 204 000), 522 (12 300). IR: *ν*_max_/cm^–1^ 2955, 2914, 2872 (m, OH), 1712 (m, CO_acid_), 1695 (m, CO_amide_), 1638 (s), 1618 (s), 1432 (s), 1385 (s), 1363 (s), 1232 (s, COC_def_), 1155 (s), 1121 (m), 839 (vs, PF), 775 (s), 770 (s), 558 (s, PF_6,def_). NMR: *δ*_H_ (400 MHz, CD_3_OD) 2.33 (3H, s, H^5^_6_), 7.95 (6H, m, H^10/10/20/20^_3/4/3/4_), 8.12 (2H, d,^3^*J*_HH_ = 8.3 Hz, H^5^_3_), 8.20 (2H, d, ^3^*J*_HH_ = 8.4 Hz, H^5^_2_), 8.24 (2H, d, ^3^*J*_HH_ = 8.3 Hz, H^15^_2_), 8.26 (4H, m, H^10/20^_2/2_), 8.45 (2H, d, ^3^*J*_HH_ = 8.3 Hz, H^15^_3_), 9.36 (8H, m, H^2,3,7,8,12,13,17,18^); *δ*_C_ (100 MHz, CD_3_OD) 24.0 (s, C^5^_6_), 120.1 (s, C^5^_3_), 124.8 (s, C^15^), 125.0 (s, C^10/20^), 127.1 (s, C^5^), 127.4 (s, C^10/20^_3/3_), 128.1 (s, C^15^_3_), 129.1 (s, C^10/20^_4/4_), 131.4 (s, C^15^_4_), 131.9 (br s, C^2/3/7/8/12/13/17/18^), 133.5 (s, C^5^_2_), 134.8 (s, C^10/20/15^_2/2/2_), 136.9 (br s, C^1/4/6/9/11/14/16/19^), 138.7 (s, C^10/20^_1/1_), 140.0 (s, C^5^_1_), 140.2 (s, C^15^_1_), 141.4 (s, C^5^_4_), 165.9 (s, C^15^_5_), 170.9 (s, C^5^_5_). *δ*_P_ (162 MHz, CD_3_OD) –143.5 (sept, ^1^*J*_PF_ = 710 Hz). MS (ESI): *m*/*z* 910.18 (100%) [M]^+^. HR-MS (ESI): *m*/*z* 910.2115 (calcd for C_47_H_31_AuN_5_O_3_: 910.2093). CV (Fc/Fc^+^, MeOH): *E*_½_/V –1.030.

### [5-(4-(*N*-Acetylaminophenyl))-10,20-di((4-butoxy)phenyl)-15-(4-(carboxyphenyl))porphyrinato]gold(iii) hexafluorophosphate **[4b][PF_6_]**

5-(4-(*N*-Acetylaminophenyl))-10,20-di((4-butoxy)phenyl)-15-(4-(carboxyphenyl))porphyrin **IVb** (75 mg, 0.09 mmol), potassium tetrachloridoaurate(iii) (133 mg, 0.36 mmol) and sodium acetate (328 mg, 4.00 mmol) were dissolved in glacial acetic acid (40 mL). The reaction mixture was heated to reflux for 24 h, allowed to cool to room temperature, and diluted with dichloromethane (200 mL). The mixture was washed with water (2 × 100 mL), saturated aqueous sodium carbonate solution (2 × 100 mL) and water (1 × 100 mL), dried over anhydrous magnesium sulfate and filtered. The filtrate was evaporated to dryness and the residue dissolved in dichloromethane (100 mL). The organic phase was stirred with a saturated aqueous solution of potassium hexafluorophosphate (20 mL) for 72 h. The mixture was diluted with dichloromethane (100 mL) and washed with water (2 × 50 mL), dried over anhydrous magnesium sulfate and filtered. The filtrate was removed under reduced pressure and the residue purified by chromatography over silica [dichloromethane : methanol (10 : 1); *R*_f_ = 0.40] to yield **[4b][PF_6_]** (72 mg, 0.06 mmol, 66%) as a purple solid. C_55_H_47_AuF_6_N_5_O_5_P (1199.9). UV/Vis: *λ*_max_(THF)/nm 422 (*ε*/M^–1^ cm^–1^ 86 000), 527 (9100), 571 (3200). IR: *ν*_max_/cm^–1^ 2957, 2924, 2870, 2855 (m, OH), 1716 (sh, CO_acid_), 1699 (s, CO_amide_), 1605 (s), 1505 (m), 1247 (s, COC_def_), 843 (vs, PF), 804 (s), 558 (s, PF_6,def_). NMR: *δ*_H_ (400 MHz, d_8_-THF) 1.10 (6H, t, ^3^*J*_HH_ = 7.3 Hz, H^10/20^_8/8_), 1.66 (4H, m, H^10/20^_7/7_), 1.96 (4H, m, H^10/20^_6/6_), 2.14 (3H, s, H^5^_6_), 4.30 (2H, t, ^3^*J*_HH_ = 6.4 Hz, H^10/20^_5/5_), 7.44 (2H, d, ^3^*J*_HH_ = 7.4 Hz, H^10/20^_3/3_), 8.12 (4H, m, H^5/5^_2/3_), 8.17 (2H, d, ^3^*J*_HH_ = 7.3 Hz, H^10/20^_2/2_), 8.37 (2H, d, ^3^*J*_HH_ = 7.1 Hz, H^15^_2_), 8.53 (2H, d, ^3^*J*_HH_ = 7.0 Hz, H^15^_3_), 9.36 (8H, m, H^2,3,7,8,12,13,17,18^), 9.48 (1H, s, NH); *δ*_C_ (100 MHz, d_8_-THF) 14.3 (s, C^10/20^_8/8_), 20.4 (s, C^10/20^_7/7_), 24.4 (s, C^5^_6_), 32.5 (s, C^10/20^_6/6_), 67.6 (s, C^10/20^_5/5_), 114.7 (s, C^10/20^_3/3_), 118.8 (s, C^5^_3_), 122.9 (s, C^15^), 124.7 (s, C^10/20^), 125.1 (s, C^5^), 129.8 (s, C^15^_3_), 131.7 (s, C^10/20^_1/1_), 132.5 (s, C^15^_4_), 133.1–133.8 (br s, C^2/3/7/8/12/13/17/18^), 135.2 (s, C^15^_2_), 135.7 (s, C^5^_2_), 136.6 (s, C^10/20^_2/2_), 137.6 (s, C^5^_1_), 138.5 (br s, C^1/4/6/9/11/14/16/19^), 141.3 (s, C^5^_4_), 143.9 (s, C^15^_1_), 161.5 (s, C^10/20^_4/4_), 167.5 (s, C^15^_5_), 169.3 (s, C^5^_5_). *δ*_P_ (162 MHz, d_8_-THF) –143.5 (sept, ^1^*J*_PF_ = 710 Hz). MS (ESI): *m*/*z* 1054.26 (100%) [M]^+^. HR-MS (ESI): *m*/*z* 1054.3218 (calcd for C_55_H_47_AuN_5_O_5_: 1054.3243). CV (Fc/Fc^+^, THF): *E*_½_/V –2.450, –1.745, –1.070.

### [5-(4-(*N*-Acetylaminophenyl))-10,20-bis(4-(trifluoromethylphenyl))-15-(4-(carboxyphenyl))porphyrinato]gold(iii) hexafluorophosphate **[4c][PF_6_]**

5-(4-(*N*-Acetylaminophenyl))-10,20-bis(4-(trifluoromethylphenyl))-15-(4-(carboxyphenyl))porphyrin **IVc** (63 mg, 0.07 mmol), potassium tetrachlorido aurate(iii) (104 mg, 0.28 mmol), and sodium acetate (258 mg, 3.15 mmol) were dissolved in glacial acetic acid (20 mL). The reaction mixture was heated to reflux for 24 h, allowed to cool to room temperature and diluted with dichloromethane (100 mL). The mixture was washed with water (2 × 50 mL), saturated aqueous sodium carbonate solution (2 × 50 mL) and water (1 × 50 mL), dried over anhydrous magnesium sulfate and filtered. The filtrate was evaporated to dryness and the residue dissolved in dichloromethane (50 mL). The organic phase was stirred with a saturated aqueous solution of potassium hexafluorophosphate (10 mL) for 72 h. The mixture was diluted with dichloromethane (50 mL) and washed with water (2 × 50 mL), dried over anhydrous magnesium sulfate and filtered. The filtrate was removed under reduced pressure and the residue purified by chromatography over silica [dichloromethane : methanol (10 : 1); *R*_f_ = 0.30] to yield **[4c][PF_6_]** (71 mg, 0.06 mmol, 85%) as a purple, crystalline solid. C_49_H_29_AuF_12_N_5_O_3_P (1191.7). UV/Vis: *λ*_max_(THF)/nm 409 (*ε*/M^–1^ cm^–1^ 170 000), 525 (15 100), 571 (3200). IR: *ν*_max_/cm^–1^ 2959, 2922, 2851 (w, OH), 1721 (s, CO_acid_), 1689 (s, CO_amide_), 1616 (m), 1591 (m), 1515 (m), 1406 (m), 1324 (vs, CF), 1168 (m), 1126 (m), 1109 (m), 1069 (s), 1034 (m), 1017 (s), 842 (vs, PF), 820 (s), 800 (s), 706 (m), 556 (s, PF_6,def_). NMR: *δ*_H_ (400 MHz, d_8_-THF) 2.21 (3H, s, H^5^_6_), 8.08 (2H, d, ^3^*J*_HH_ = 8.2 Hz, H^5^_3_), 8.14 (2H, d, ^3^*J*_HH_ = 8.2 Hz, H^5^_2_), 8.25 (2H, d, ^3^*J*_HH_ = 7.5 Hz, H^10/20^_2/2_), 8.39 (2H, d, ^3^*J*_HH_ = 7.1 Hz, H^15^_2_), 8.53 (2H, d, ^3^*J*_HH_ = 7.5 Hz, H^10/20^_3/3_), 8.53 (2H, d, ^3^*J*_HH_ = 7.5 Hz, H^15^_3_), 9.40 (8H, m, H^2,3,7,8,12,13,17,18^), 9.41 (1H, s, NH); *δ*_C_ (100 MHz, d_8_-THF) 24.3 (s, C^5^_6_), 118.8 (s, C^5^_3_), 122.9 (s, C^5^), 123.5 (s, C^15^), 124.3 (s, C^10/20^), 125.6 (s, C^10/20^_2/2_), 127.0 (s, C^10/20^_4/4_), 129.8 (s, C^15^_3_), 132.4 (s, C^15^_4_), 133.1–133.8 (multiple s, C^2/3/7/8/12/13/17/18^), 135.3 (s, C^15^_2_), 135.7 (s, C^5/5/10/10^_2/3/2/3_), 137.7 (s, C^5^_1_), 137.9–138.7 (multiple s, C^1/4/6/9/11/14/16/19^), 142.2 (s, C^5^_4_), 143.9 (s, C^10/15/20^_1/1/1_), 161.5 (s, C^10/20^_4/4_), 167.5 (s, C^15^_5_), 169.2 (s, C^5^_5_). *δ*_P_ (162 MHz, d_8_-THF) –143.3 (sept, ^1^*J*_PF_ = 711 Hz). MS (ESI): *m*/*z* 1045.97 (100%) [M]^+^. HR-MS (ESI): *m*/*z* 1046.1863 (calcd for C_49_H_29_AuF_6_N_5_O_3_: 1046.1840). CV (Fc/Fc^+^, THF): *E*_½_/V –2.300, –1.590, –0.990.

## Conclusions

Auration of *meso*-tetraaryl substituted AB_2_C porphyrins with KAuCl_4_ in the presence of HOAc/NaOAc cleanly gives the corresponding gold(iii) porphyrinato complex cations. Amino-substituted porphyrins are *N*-acetylated under these conditions and have to be prepared from the corresponding nitro-substituted gold(iii) porphyrins by reduction with SnCl_2_/HCl. The gold(iii) complexes can be reduced at least three times. The potentials slightly depend on the electron withdrawing and donating nature of the substituents. The first reduction is addressed by UV/Vis spectroelectrochemistry and by EPR spectroscopy. Upon one-electron reduction, the Soret band experiences a small bathochromic shift. The intensity of the Soret band of the electron rich complexes **[2a]^+^** (R^2^ = NH_2_) and **[4b]^+^** (R^3^ = O^*n*^Bu) slightly increases upon reduction while all other neutral complexes feature less intense Soret bands as compared to their parent Au^III^ complexes. These spectral data clearly suggest the presence of an unreduced porphyrinato ligand in all cases under these conditions. Chemical one-electron reduction of the porphyrinato gold(iii) hexafluorophosphate salts by cobaltocene yields the corresponding Au^II^ porphyrin complexes with a characteristic EPR pattern displaying hyperfine coupling to ^197^Au and ^14^N. The degree of ^197^Au hfc and *g* anisotropy places the gold contribution to the spin density in (tetraphenylporphyrinato)gold(ii) complexes in between that of [Au(en)_2_]^2+^ (ref. 15) and the neutral gold hematoporphyrin IX complex.13*e* DFT calculations fully support the metal centred reduction in all cases, essentially irrespective of the substituent at the *meso* aryl groups. Only, the nitro substituent reduction competes significantly with the Au^III^ reduction and a valence isomeric equilibrium between the Au^II^ valence isomer and the nitro π radical anion valence isomer is established. DFT calculations suggest that the position of the counterion triggers the position of the equilibria between the different valence isomers that interconvert by an intramolecular electron transfer process. These findings allows the usage of *meso*-substituted Au^III^ porphyrins as electron acceptors and electron storage materials in photo-induced redox processes, almost irrespective of the substitution pattern. Hence, the substituents fine-tune the redox potential or other properties such as solubility without compromising the thermodynamically preferred metal site of one-electron reduction. Combination of electron-accepting gold(iii) porphyrins bearing carboxylic acid, amine and amide substituents, as introduced in this report, with light-harvesting porphyrins and electron donating porphyrins *via* amide connectivity^17^ are currently explored in our laboratory and will be reported in due course.

## Supplementary Material

Supplementary information
