# Supplementary material for: Gold(iii) tetraarylporphyrin amino acid derivatives: ligand or metal centred redox chemistry?
Source: Chem Sci. 2015 Oct 26;7(1):596–610. doi: 10.1039/c5sc03429a (PMC5952892; doi:10.1039/c5sc03429a)
Supplement: Supplementary file 1 [file SC-007-C5SC03429A-s001.pdf]

Electronic Supplementary Material (ESI) for Chemical Science.
This journal is © The Royal Society of Chemistry 2015

# Gold(III) tetraarylporphyrin amino acid derivatives:

# ligand or metal centred redox chemistry?

Sebastian Preiß, Jascha Melomedov, Anica Wünsche von Leupoldt and Katja Heinze*

## Supporting Information

**Fig. S01** $^{1}H$ NMR spectrum of **[Au(TPP)](PF$_6$)** in $CD_2Cl_2$.

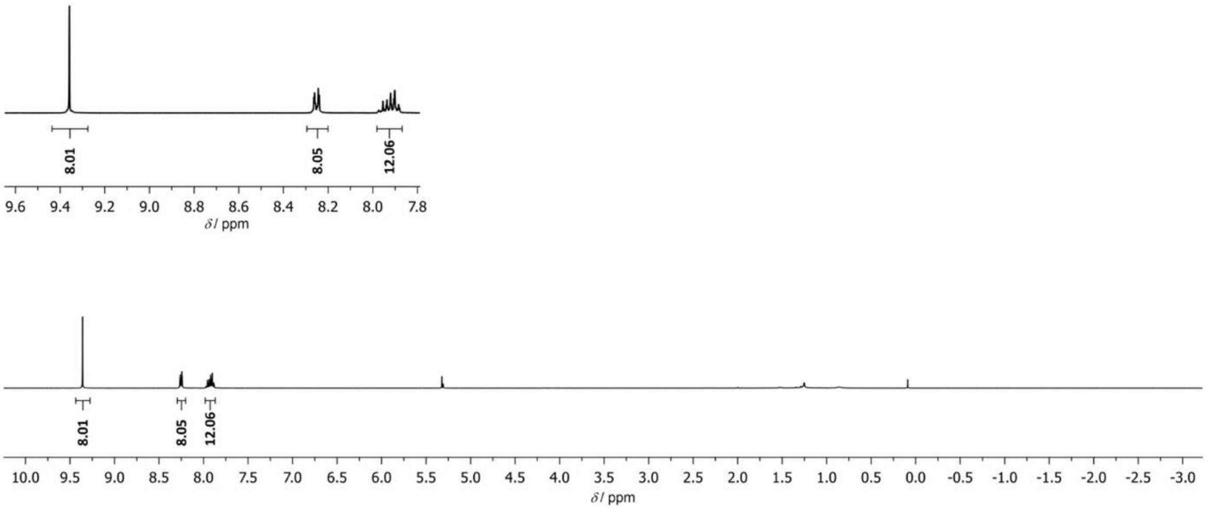


**Fig. S02** $^{13}C$ NMR spectrum of **[Au(TPP)](PF$_6$)** in $CD_2Cl_2$.

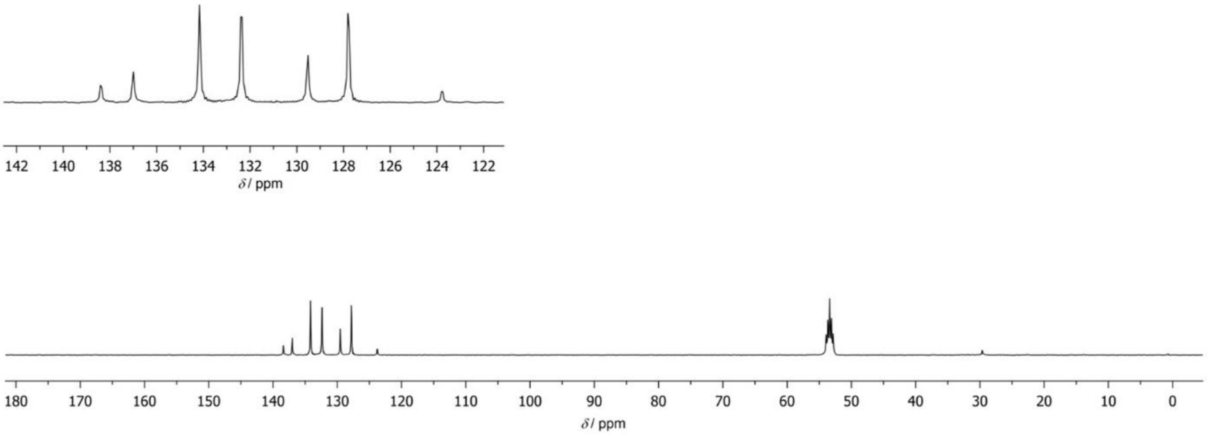

**Fig. S03** $^{1}$H NMR spectrum of **[1a](PF$_6$)** in $CD_2Cl_2$.

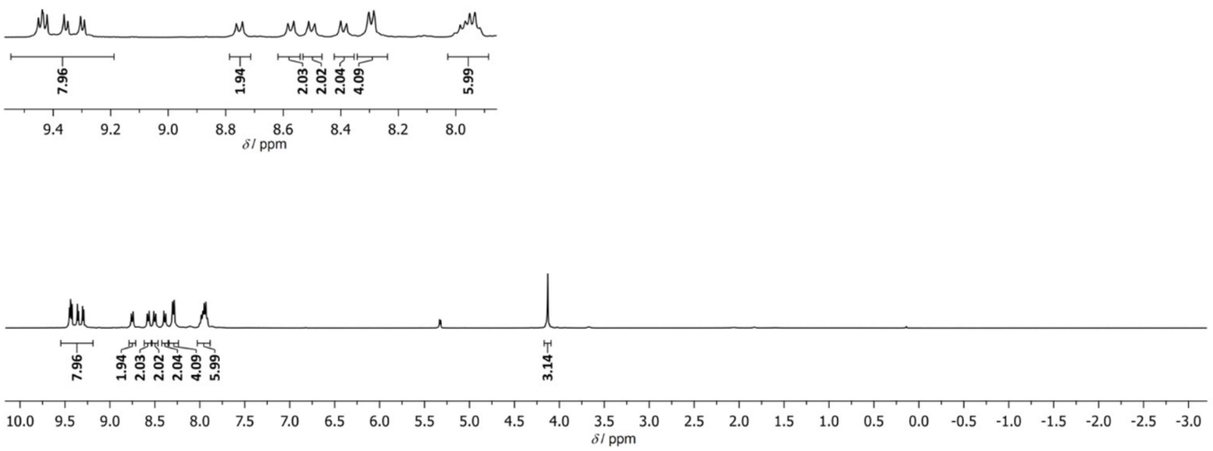


**Fig. S04** $^{13}$C NMR spectrum of **[1a](PF$_6$)** in $CD_2Cl_2$.

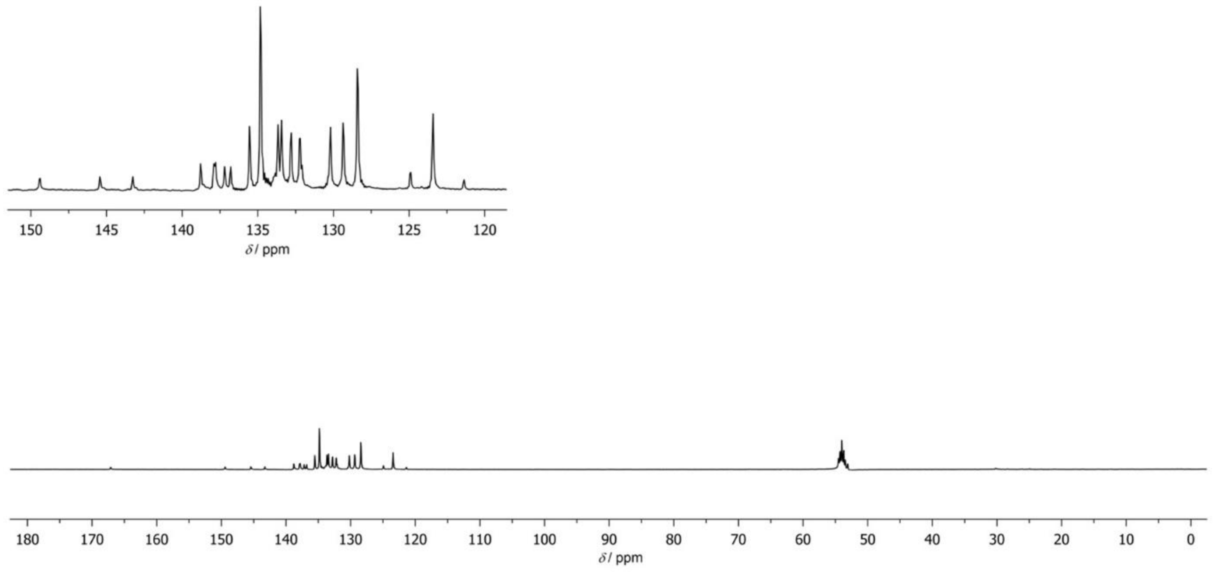

**Fig. S05** $^{1}H$ NMR spectrum of **[2a](PF$_6$)** in $CD_2Cl_2$.

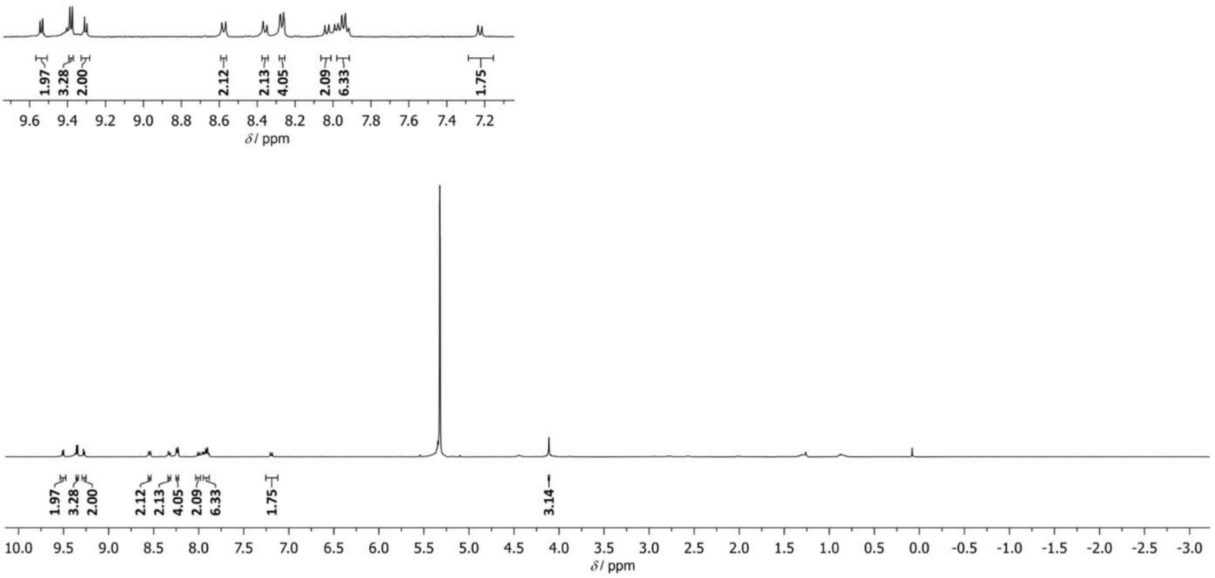


**Fig. S06** $^{13}C$ NMR spectrum of **[2a](PF$_6$)** in $CD_2Cl_2$.

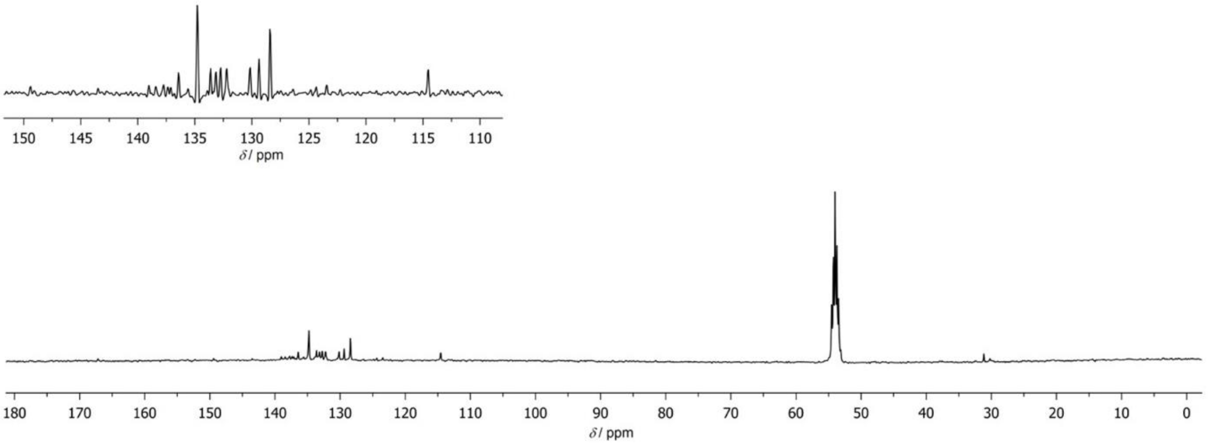

**Fig. S07** $^{1}H$ NMR spectrum of **[3a](PF$_6$)** in $CD_2Cl_2$.

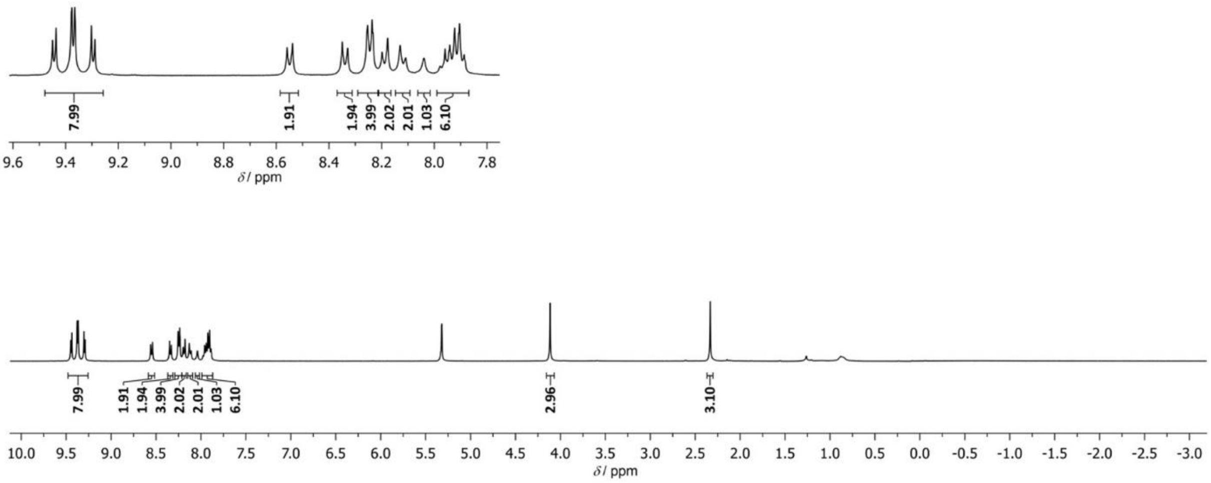


**Fig. S08** $^{13}C$ NMR spectrum of **[3a](PF$_6$)** in $CD_2Cl_2$.

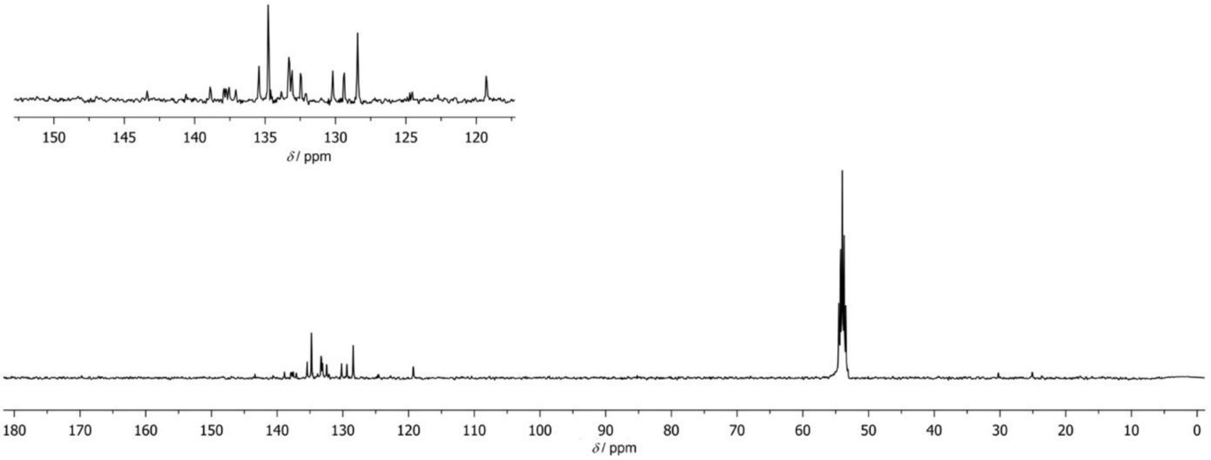

**Fig. S09** $^{1}H$ NMR spectrum of **[4a](PF$_6$)** in $CD_3OD$.

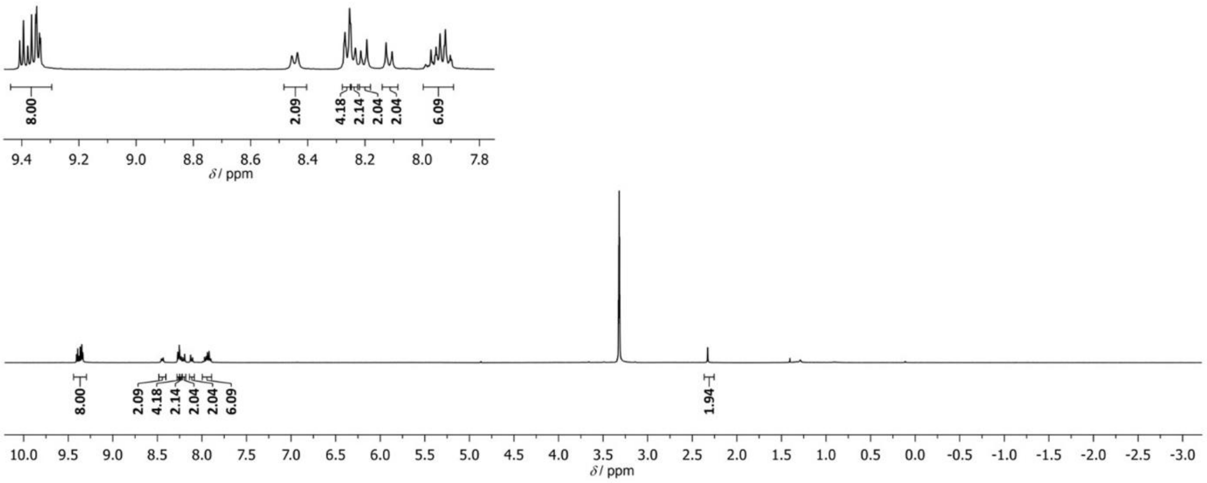


**Fig. S10** $^{13}C$ NMR spectrum of **[4a](PF$_6$)** in $CD_3OD$.

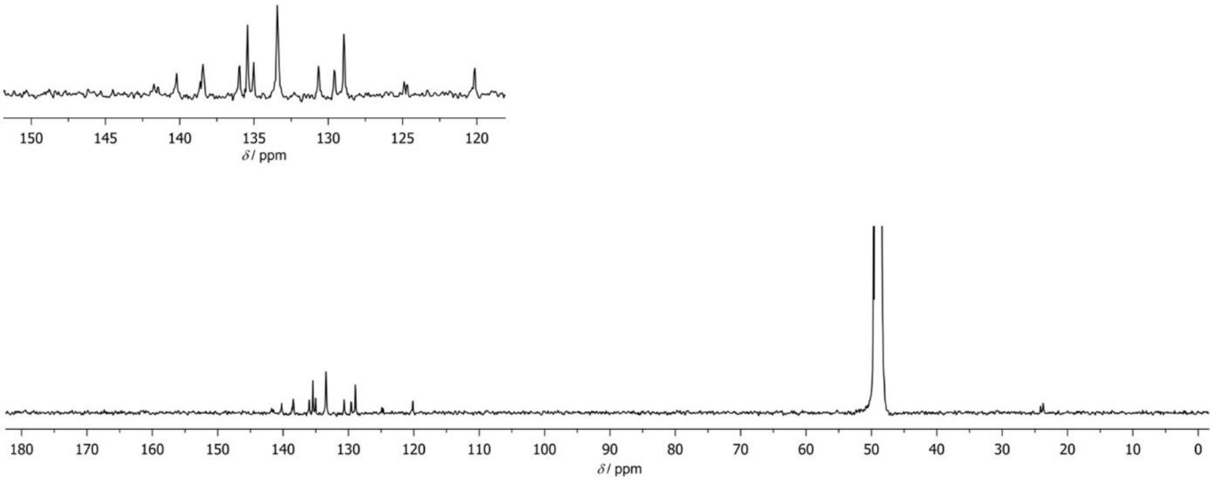

**Fig. S11** $^{1}$H NMR spectrum of **[4b](PF$_6$)** in d$_8$-THF.

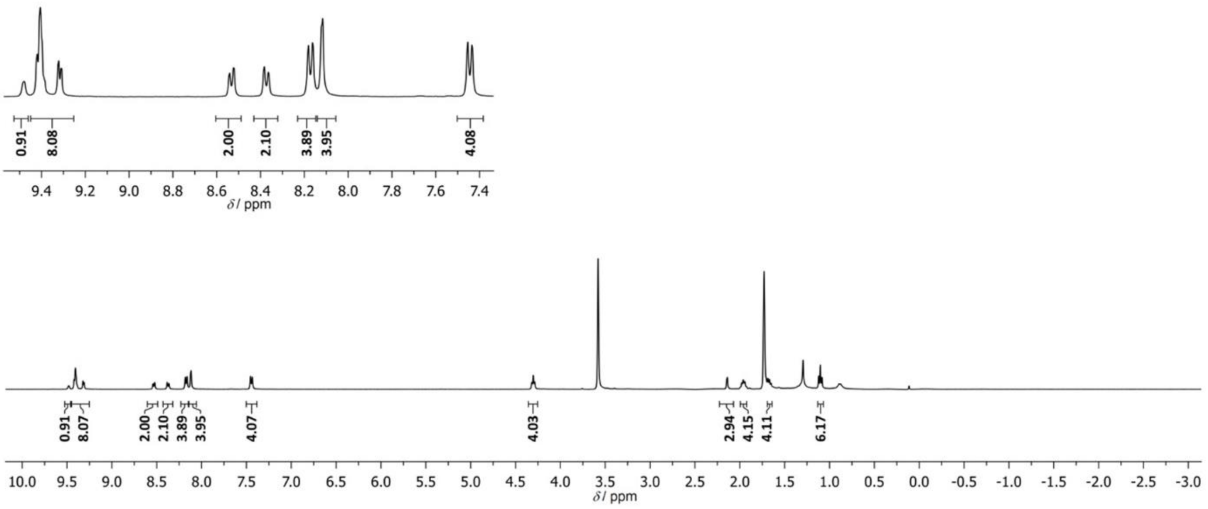


**Fig. S12** $^{13}$C NMR spectrum of **[4b](PF$_6$)** in d$_8$-THF.

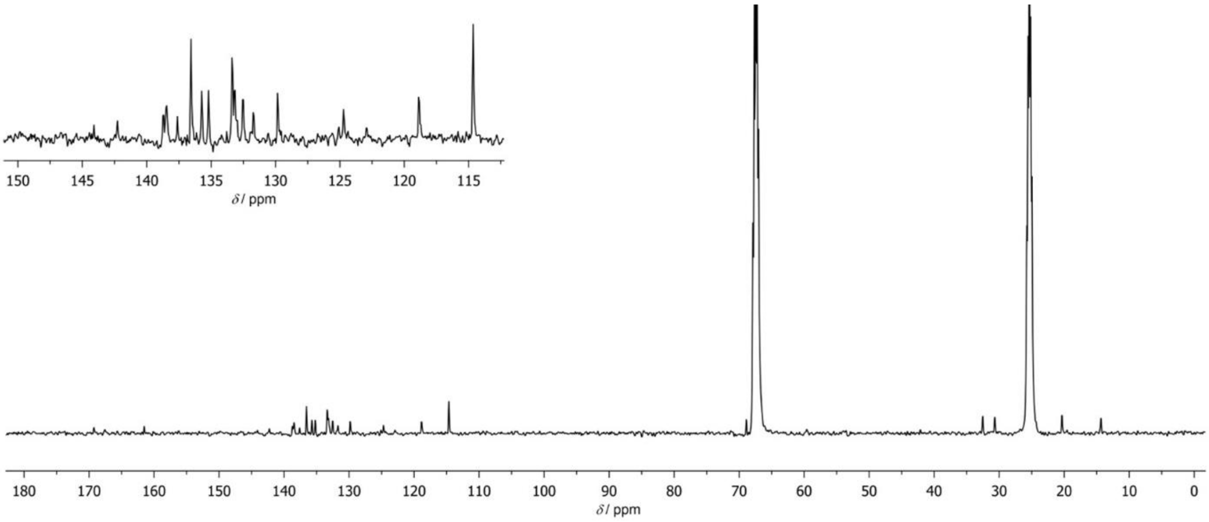

**Fig. S13** $^{1}H$ NMR spectrum of **[4c](PF$_6$)** in $d_8$-THF.

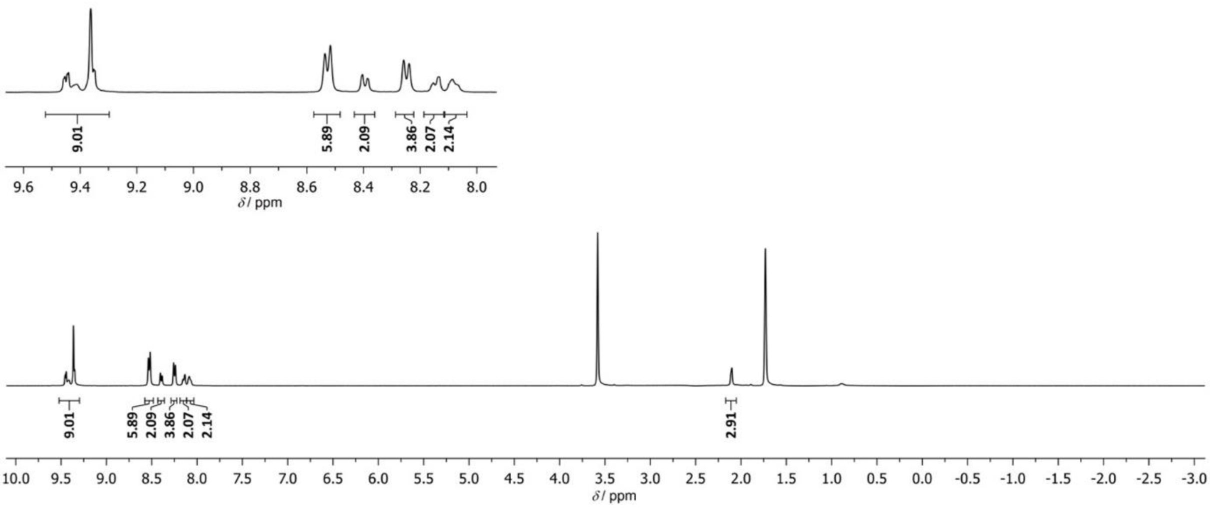


**Fig. S14** $^{13}C$ NMR spectrum of **[4c](PF$_6$)** in $d_8$-THF.

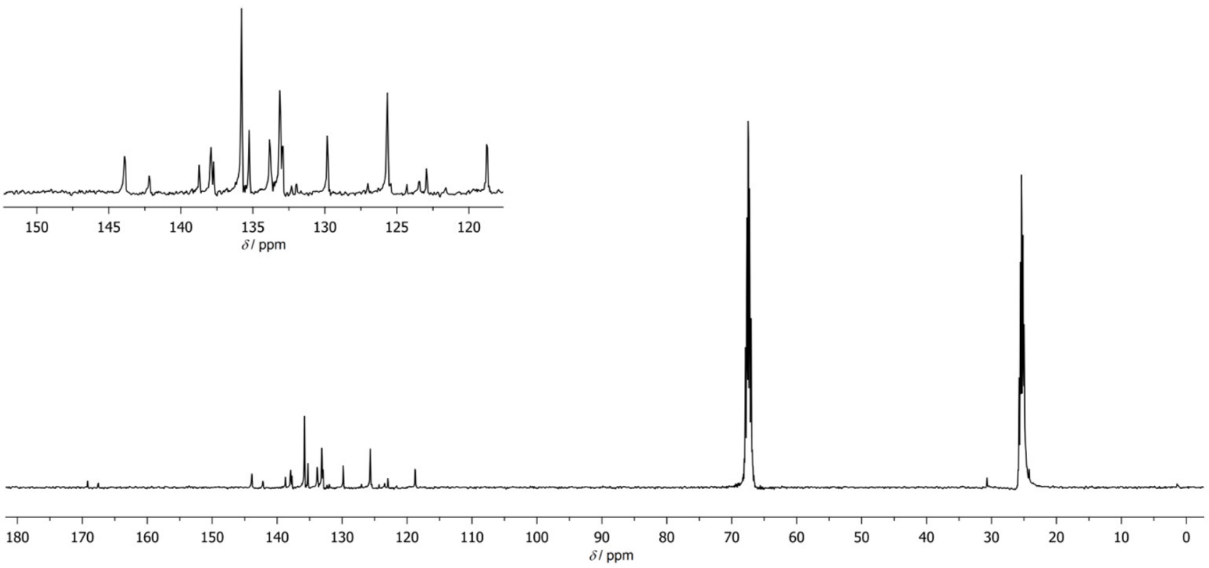

**Fig. S15** IR spectrum of [Au(TPP)]($PF_6$) as KBr disk.

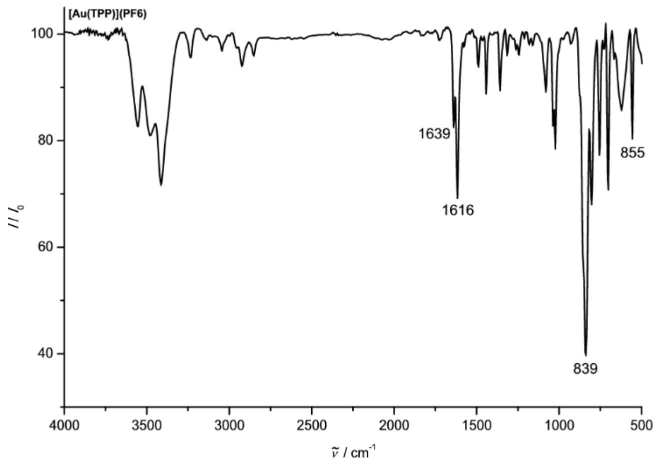


**Fig. S16** IR spectrum of **[1a]($PF_6$)** as KBr disk.

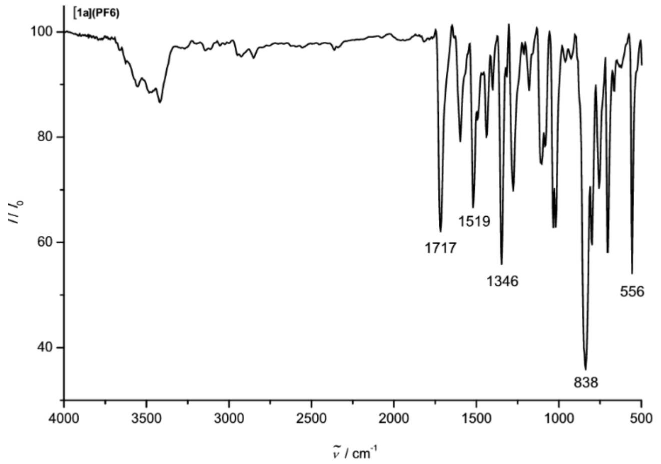


**Fig. S17** IR spectrum of **[2a]($PF_6$)** as KBr disk.

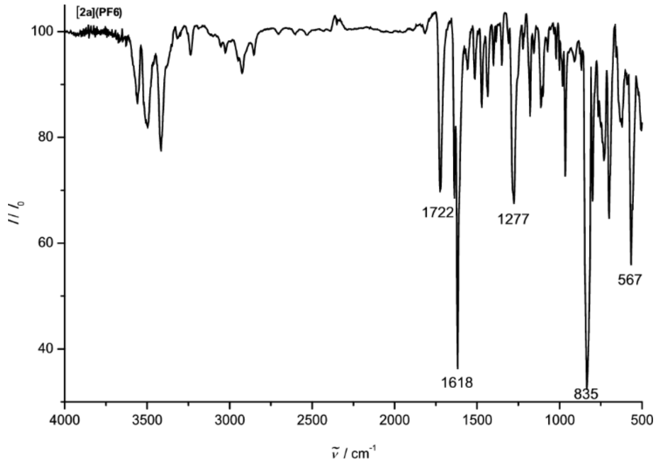

**Fig. S18** IR spectrum of **[3a](PF$_6$)** as KBr disk.

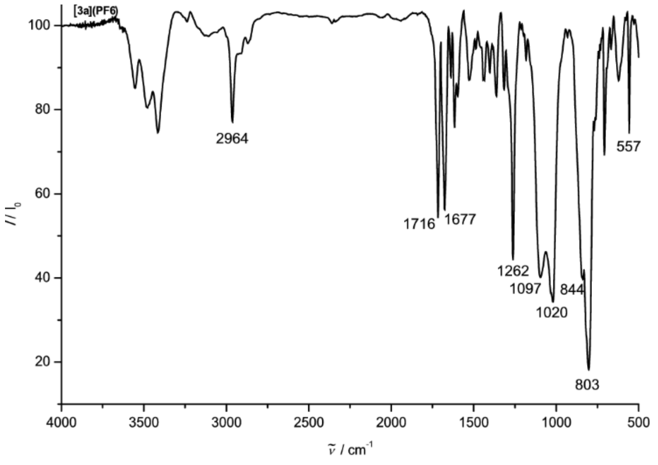


**Fig. S19** IR spectrum of **[4a](PF$_6$)** as KBr disk.

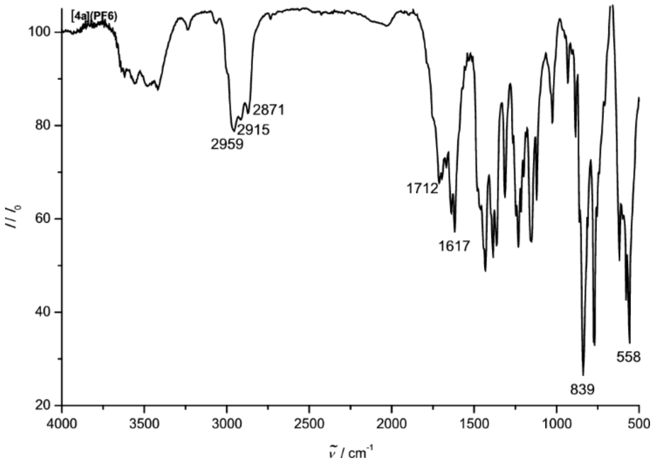


**Fig. S20** IR spectrum of **[4b](PF$_6$)** as KBr disk.

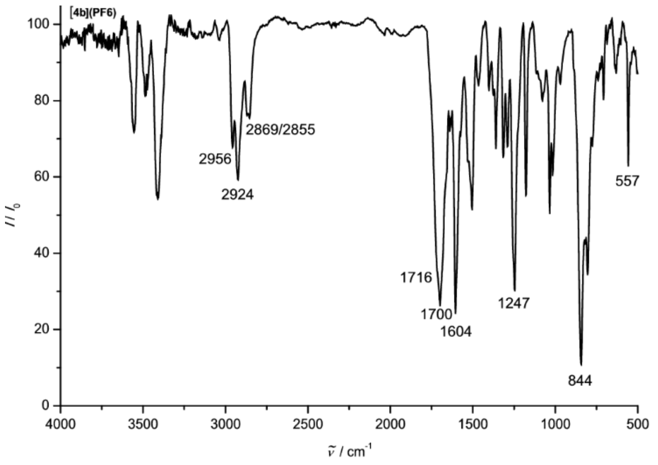

**Fig. S21** IR spectrum of **[4c](PF$_6$)** as KBr disk.

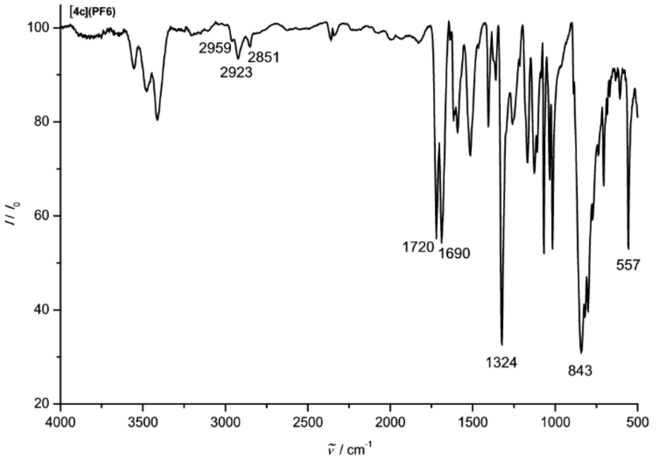

**Fig. S22** UV/Vis spectral changes upon the first electroreduction of [Au(TPP)]$^+$ $10^{-5}$ M in 0.1 M [$^n$Bu$_4$N](PF$_6$)/THF solution (isosbestic points indicated in blue).

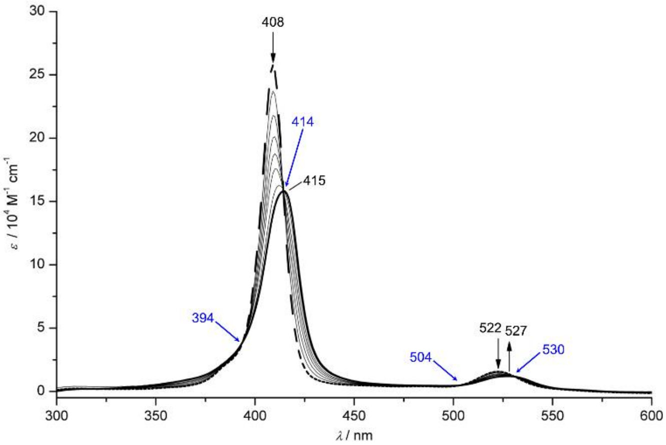


**Fig. S23** UV/Vis spectral changes upon the first electroreduction of a) **[4a]**$^+$ (in MeOH), b) **[4b]**$^+$ and c) **[4c]**$^+$ $10^{-5}$ M in 0.1 M [$^n$Bu$_4$N](PF$_6$) THF solution (isosbestic points indicated in blue).

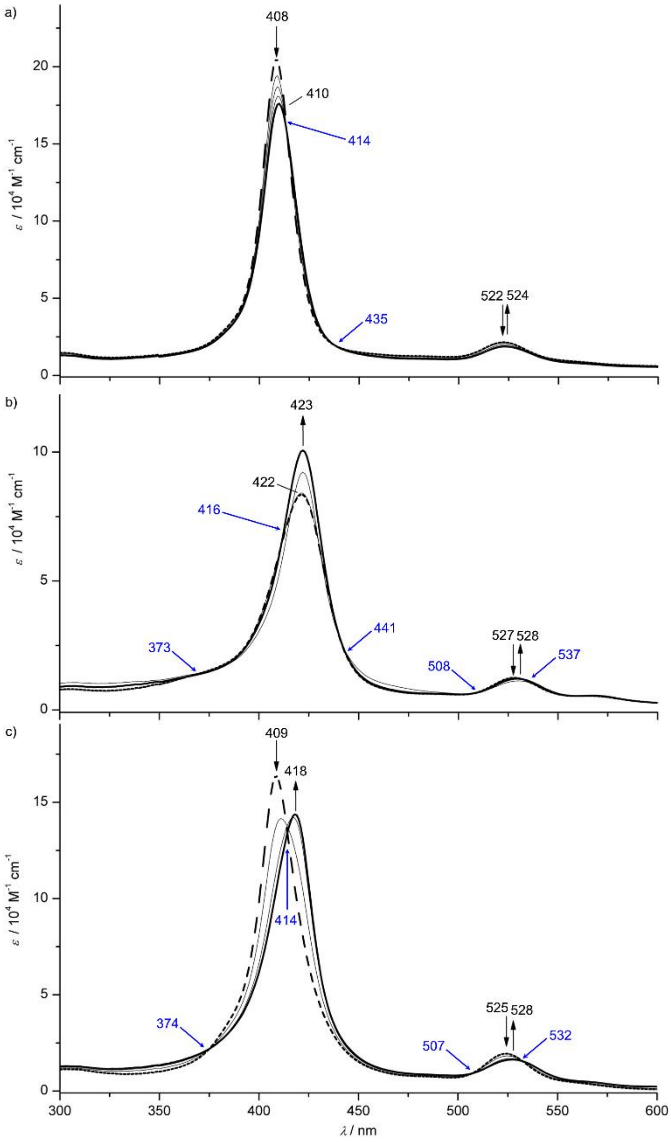

**Fig. S24** X-band EPR spectrum of **1a** /**1a'**/**1a''** in frozen $CH_2Cl_2$ solution (77 K, 9.4 GHz) and deconvolution in the respective component spectra.

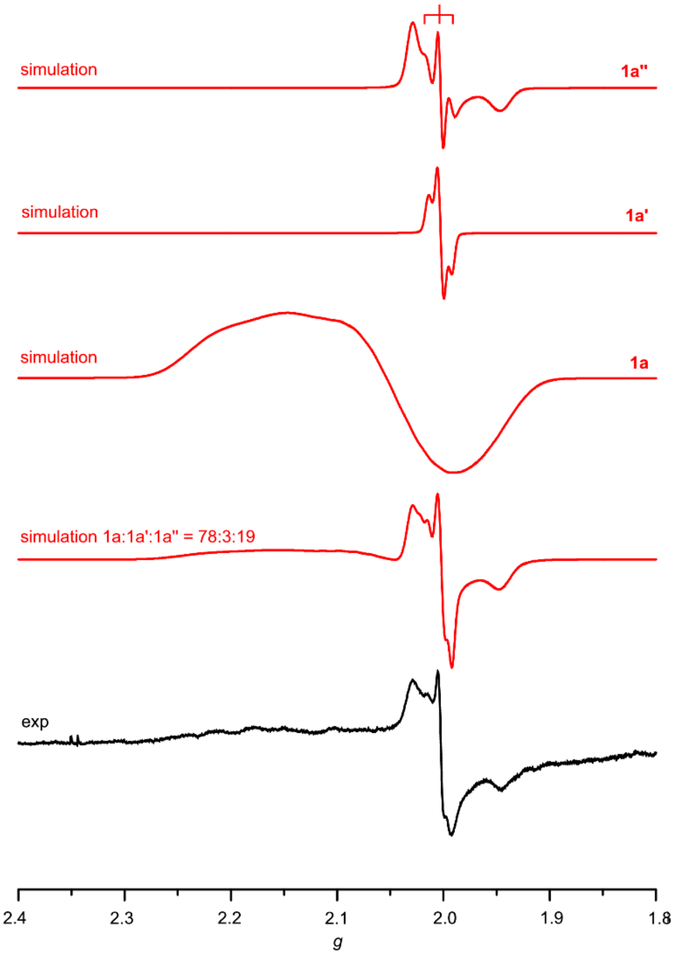

**Fig. S25** X-band EPR spectrum of **1a** in frozen $CH_2Cl_2$ solution (77 K, 9.4 GHz) in the absence and in the presence of two equivalents [$^{n}Bu_4N$]Cl.

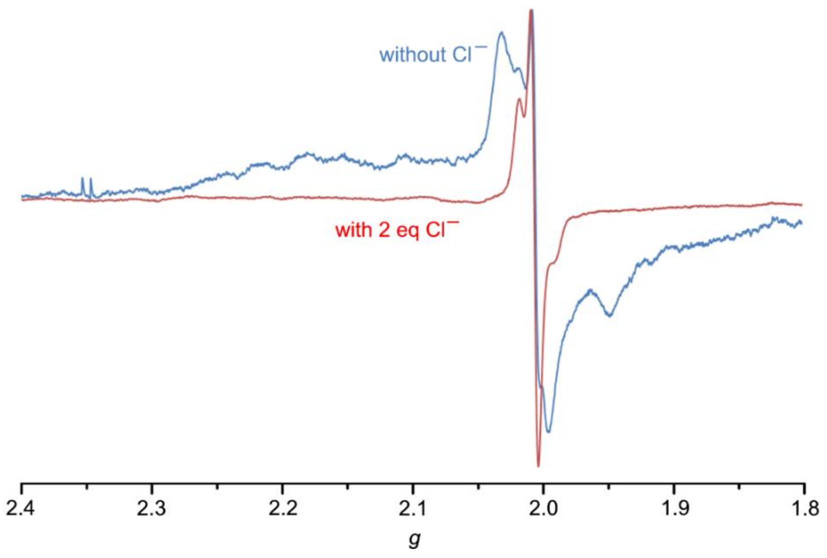


**Fig. S26** X-band EPR spectrum of **1a** in frozen $CH_2Cl_2$ solution (77 K, 9.4 GHz) in the presence of two equivalents [$^{n}Bu_4N$]Cl and corresponding simulation.

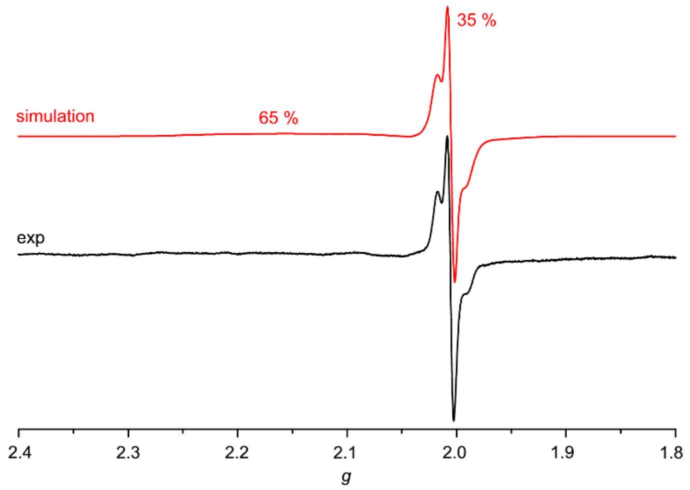

**Fig. S27** Spin densities of DFT optimized geometries of Au(TPP) and Cu(TPP) (isosurface value 0.01 a.u.; UB3LYP, LANL2DZ, IEFPCM $CH_2Cl_2$; Mulliken spin densities in italics).

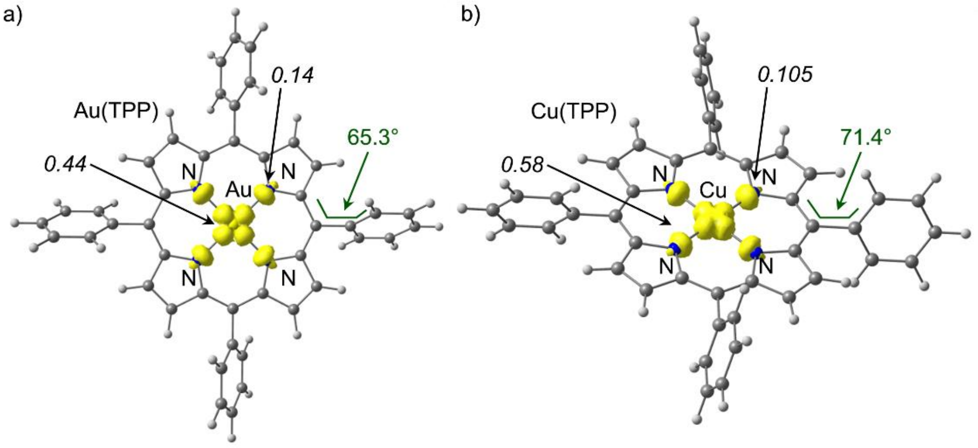


**Fig. S28** Spin densities of DFT optimized geometries of **4a**, **4b** and **4c** (isosurface value 0.01 a.u.; UB3LYP, LANL2DZ, IEFPCM $CH_2Cl_2$; Mulliken spin densities in italics).

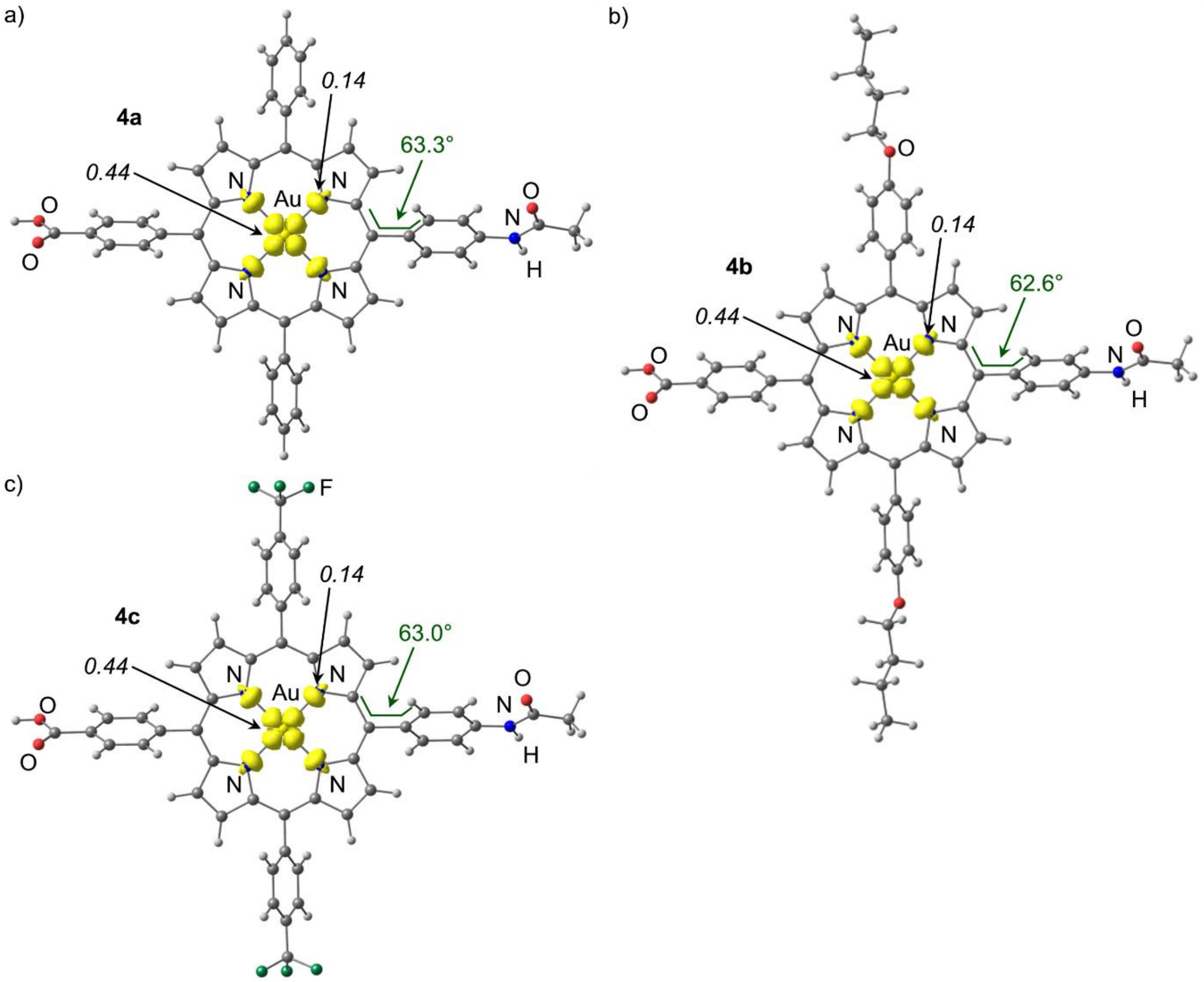

**Fig. S29** X-band EPR spectrum of **2a** / **2a'** in frozen $CH_2Cl_2$ (top) and THF (bottom) solution (77 K, 9.4 GHz).

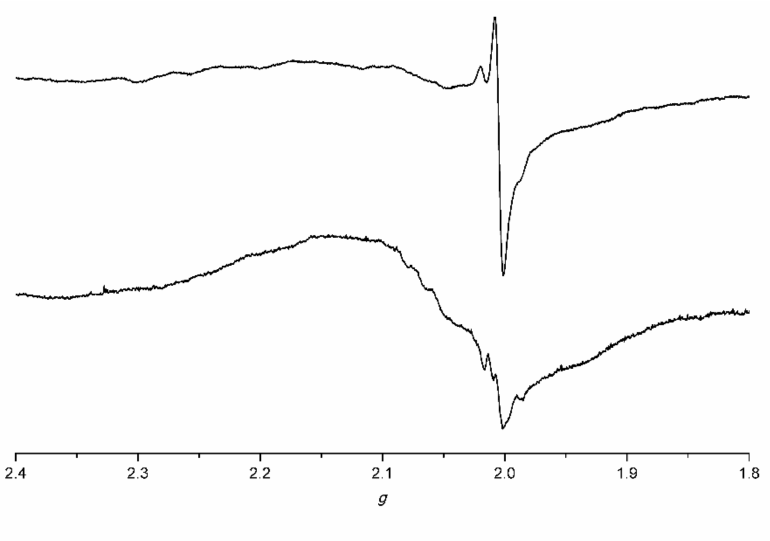


**Fig. S30** X-band EPR spectrum of **3a** / **3a'** in frozen $CH_2Cl_2$ (top) and THF (bottom) solution (77 K, 9.4 GHz).

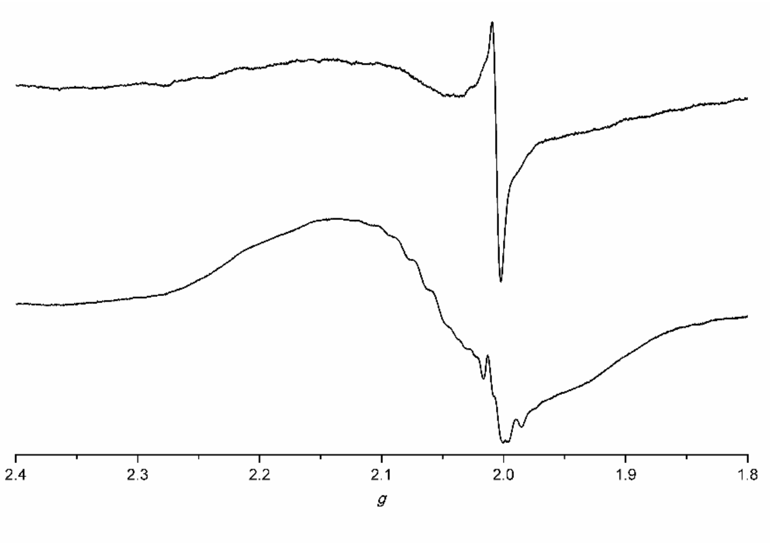

**Cartesian coordinates of DFT optimised geometries (B3LYP, LANL2DZ, IEFPCM $CH_2Cl_2$)**

$[Au(TPP)]^+$

| | | | |
|---|---|---|---|
| 7 | -1.498254000 | 1.401284000 | -0.009938000 |
| 7 | 1.401502000 | 1.497628000 | 0.007722000 |
| 7 | -1.401410000 | -1.497574000 | 0.007417000 |
| 7 | 1.498342000 | -1.401185000 | -0.009973000 |
| 6 | -2.871537000 | 1.139197000 | 0.068251000 |
| 6 | -3.567765000 | 2.397553000 | 0.219138000 |
| 6 | -2.629510000 | 3.400467000 | 0.211938000 |
| 6 | -1.327784000 | 2.788967000 | 0.058771000 |
| 1 | -4.636175000 | 2.494941000 | 0.332148000 |
| 1 | -2.797515000 | 4.460639000 | 0.318660000 |
| 6 | -0.116499000 | 3.497526000 | -0.008172000 |
| 6 | -3.498186000 | -0.116641000 | 0.007945000 |
| 6 | 2.397515000 | 3.567776000 | -0.218771000 |
| 6 | 3.400577000 | 2.629800000 | -0.211839000 |
| 6 | 2.789229000 | 1.327730000 | -0.058905000 |
| 6 | 1.139245000 | 2.870961000 | -0.068668000 |
| 1 | 2.494361000 | 4.636273000 | -0.331481000 |
| 1 | 4.460731000 | 2.797840000 | -0.318598000 |
| 6 | 3.498193000 | 0.116678000 | 0.008230000 |
| 6 | -1.139107000 | -2.870850000 | -0.069090000 |
| 6 | -2.397293000 | -3.567699000 | -0.219400000 |
| 6 | -3.400423000 | -2.629737000 | -0.212808000 |
| 6 | -2.789200000 | -1.327707000 | -0.059526000 |
| 1 | -2.494129000 | -4.636206000 | -0.332027000 |
| 1 | -4.460528000 | -2.797890000 | -0.319869000 |
| 6 | 3.568029000 | -2.397616000 | 0.217675000 |
| 6 | 2.629812000 | -3.400494000 | 0.210591000 |
| 6 | 1.327919000 | -2.788871000 | 0.058354000 |
| 6 | 2.871586000 | -1.139130000 | 0.067982000 |
| 1 | 4.636540000 | -2.494914000 | 0.329921000 |
| 1 | 2.797804000 | -4.460745000 | 0.316665000 |
| 6 | 0.116680000 | -3.497418000 | -0.008436000 |
| 6 | -0.167814000 | 4.997202000 | -0.015274000 |
| 6 | -0.763263000 | 5.688843000 | -1.095142000 |
| 6 | -0.805972000 | 7.095310000 | -1.104938000 |
| 6 | -0.265706000 | 7.827725000 | -0.029047000 |
| 6 | 0.323714000 | 7.144931000 | 1.053587000 |
| 6 | -4.997791000 | -0.167948000 | 0.016166000 |
| 6 | -5.689044000 | -0.764531000 | 1.095645000 |
| 6 | -7.095500000 | -0.807411000 | 1.105954000 |
| 6 | -7.828577000 | -0.266216000 | 0.030955000 |
| 6 | -7.146058000 | 0.324401000 | -1.051289000 |
| 6 | -5.740059000 | 0.378839000 | -1.056056000 |
| 1 | -5.129835000 | -1.179244000 | 1.930589000 |
| 1 | -7.613949000 | -1.260634000 | 1.946700000 |
| 1 | -5.219931000 | 0.829912000 | -1.897330000 |
| 6 | 0.167881000 | -4.997110000 | -0.015260000 |
| 6 | -0.377759000 | -5.738452000 | 1.058095000 |
| 6 | -0.323635000 | -7.144563000 | 1.054141000 |
| 6 | 0.265672000 | -7.827619000 | -0.028394000 |
| 6 | 0.805909000 | -7.095470000 | -1.104481000 |
| 6 | 0.763250000 | -5.688994000 | -1.094999000 |
| 6 | 4.997842000 | 0.167875000 | 0.016152000 |
| 6 | 5.689228000 | 0.763154000 | 1.096347000 |
| 6 | 7.095646000 | 0.805705000 | 1.106560000 |
| 6 | 7.828579000 | 0.265426000 | 0.030924000 |
| 6 | 7.145949000 | -0.323893000 | -1.051870000 |
| 6 | 5.739858000 | -0.377959000 | -1.056618000 |
| 1 | 5.129993000 | 1.177148000 | 1.931620000 |
| 1 | 7.614391000 | 1.257945000 | 1.947675000 |
| 1 | 7.703447000 | -0.739332000 | -1.887178000 |
| 1 | 5.219646000 | -0.828202000 | -1.898284000 |
| 6 | 0.377933000 | 5.738830000 | 1.057834000 |
| 1 | 8.914768000 | 0.302281000 | 0.037055000 |
| 1 | -7.703726000 | 0.740489000 | -1.886171000 |
| 1 | -8.914766000 | -0.303128000 | 0.036709000 |
| 1 | -0.828066000 | -5.217670000 | 1.899384000 |
| 1 | -0.738863000 | -7.701717000 | 1.889766000 |
| 1 | 1.258156000 | -7.614652000 | -1.945303000 |
| 1 | 1.177154000 | -5.130208000 | -1.930630000 |
| 1 | 0.828308000 | 5.218276000 | 1.899227000 |
| 1 | -1.177142000 | 5.129869000 | -1.930662000 |

| | | | |
|---|---|---|---|
| 1 | 0.738975000 | 7.702289000 | 1.889060000 |
| 1 | -1.258269000 | 7.614279000 | -1.945864000 |
| 1 | -0.302647000 | 8.913931000 | -0.034610000 |
| 1 | 0.302559000 | -8.913828000 | -0.033732000 |
| 79 | -0.000167000 | 0.000124000 | -0.000678000 |

Au(TPP)

| | | | |
|---|---|---|---|
| 7 | -1.998141000 | 0.719501000 | -0.028358000 |
| 7 | 0.719535000 | 1.998086000 | 0.028581000 |
| 7 | -0.719569000 | -1.998114000 | 0.028293000 |
| 7 | 1.998107000 | -0.719513000 | -0.028799000 |
| 6 | -3.136929000 | -0.074998000 | 0.022196000 |
| 6 | -4.291862000 | 0.813406000 | 0.126097000 |
| 6 | -3.825259000 | 2.109255000 | 0.126386000 |
| 6 | -2.369054000 | 2.057545000 | 0.022557000 |
| 1 | -5.319390000 | 0.491054000 | 0.206887000 |
| 1 | -4.411471000 | 3.012578000 | 0.207544000 |
| 6 | -1.496029000 | 3.179663000 | 0.000388000 |
| 6 | -3.179756000 | -1.496060000 | -0.000346000 |
| 6 | 0.813439000 | 4.291776000 | -0.126438000 |
| 6 | 2.109293000 | 3.825200000 | -0.126717000 |
| 6 | 2.057591000 | 2.369032000 | -0.022581000 |
| 6 | -0.074948000 | 3.136836000 | -0.022197000 |
| 1 | 0.491062000 | 5.319278000 | -0.207426000 |
| 1 | 3.012599000 | 4.411418000 | -0.208094000 |
| 6 | 3.179691000 | 1.496032000 | -0.000405000 |
| 6 | 0.074917000 | -3.136874000 | -0.022214000 |
| 6 | -0.813519000 | -4.291821000 | -0.126069000 |
| 6 | -2.109372000 | -3.825236000 | -0.126310000 |
| 6 | -2.057647000 | -2.369052000 | -0.022536000 |
| 1 | -0.491177000 | -5.319345000 | -0.206861000 |
| 1 | -3.012703000 | -4.411461000 | -0.207331000 |
| 6 | 4.291782000 | -0.813432000 | 0.126547000 |
| 6 | 3.825177000 | -2.109276000 | 0.126805000 |
| 6 | 2.369011000 | -2.057547000 | 0.022492000 |
| 6 | 3.136884000 | 0.074969000 | 0.022141000 |
| 1 | 5.319278000 | -0.491083000 | 0.207744000 |
| 1 | 4.411352000 | -3.012599000 | 0.208259000 |
| 6 | 1.496011000 | -3.179678000 | 0.000329000 |
| 6 | -2.136353000 | 4.540912000 | 0.000840000 |
| 6 | -2.921210000 | 4.969469000 | -1.095054000 |
| 6 | -3.516688000 | 6.245032000 | -1.097588000 |
| 6 | -3.344374000 | 7.109935000 | 0.001742000 |
| 6 | -2.568022000 | 6.690339000 | 1.100607000 |
| 6 | -4.540984000 | -2.136450000 | -0.000673000 |
| 6 | -4.969552000 | -2.921062000 | 1.095341000 |
| 6 | -6.245082000 | -3.516633000 | 1.097980000 |
| 6 | -7.109935000 | -3.344587000 | -0.001506000 |
| 6 | -6.690286000 | -2.568453000 | -1.100536000 |
| 6 | -5.418149000 | -1.965899000 | -1.097095000 |
| 1 | -4.310321000 | -3.052285000 | 1.949794000 |
| 1 | -6.560891000 | -4.109512000 | 1.952739000 |
| 1 | -5.098372000 | -1.374155000 | -1.951189000 |
| 6 | 2.136457000 | -4.540866000 | 0.000820000 |
| 6 | 1.965677000 | -5.418095000 | 1.097135000 |
| 6 | 2.568391000 | -6.690255000 | 1.100556000 |
| 6 | 3.344837000 | -7.109721000 | 0.001708000 |
| 6 | 3.517100000 | -6.244760000 | -1.097584000 |
| 6 | 2.921453000 | -4.969276000 | -1.095036000 |
| 6 | 4.540875000 | 2.136395000 | -0.000784000 |
| 6 | 4.969067000 | 2.921816000 | 1.094906000 |
| 6 | 6.244515000 | 3.517338000 | 1.097590000 |
| 6 | 7.109741000 | 3.344509000 | -0.001403000 |
| 6 | 6.690513000 | 2.567614000 | -1.100095000 |
| 6 | 5.418340000 | 1.965039000 | -1.096800000 |
| 1 | 4.309549000 | 3.053622000 | 1.949052000 |
| 1 | 6.560086000 | 4.110825000 | 1.952070000 |
| 1 | 7.348489000 | 2.431881000 | -1.954812000 |
| 1 | 5.098891000 | 1.372720000 | -1.950636000 |
| 6 | -1.965479000 | 5.418098000 | 1.097173000 |
| 1 | 8.093509000 | 3.807260000 | -0.001509000 |
| 1 | -7.347973000 | -2.433305000 | -1.955630000 |
| 1 | -8.093786000 | -3.807157000 | -0.002094000 |
| 1 | 1.373684000 | -5.098427000 | 1.951125000 |
| 1 | 2.433095000 | -7.348043000 | 1.955528000 |
| 1 | 4.110277000 | -6.560477000 | -1.952240000 |
| 1 | 3.052855000 | -4.309924000 | -1.949395000 |

| | | | |
|---|---|---|---|
| 1 | -1.373538000 | 5.098333000 | 1.951164000 |
| 1 | -3.052641000 | 4.310173000 | -1.949451000 |
| 1 | -2.432663000 | 7.348093000 | 1.955596000 |
| 1 | -4.109773000 | 6.560857000 | -1.952269000 |
| 1 | -3.807027000 | 8.093754000 | 0.002098000 |
| 1 | 3.807620000 | -8.093479000 | 0.002056000 |
| 79 | -0.000023000 | -0.000018000 | -0.000189000 |

Cu(TPP)

| | | | |
|---|---|---|---|
| 29 | -0.000030000 | -0.000037000 | 0.000697000 |
| 7 | -0.013602000 | -2.029563000 | 0.000231000 |
| 7 | -2.029528000 | 0.013068000 | -0.000038000 |
| 7 | 2.029437000 | -0.013103000 | 0.000276000 |
| 7 | 0.013525000 | 2.029512000 | 0.000516000 |
| 6 | 1.098736000 | -2.876899000 | -0.006284000 |
| 6 | 0.658060000 | -4.262677000 | -0.004861000 |
| 6 | -0.714372000 | -4.253739000 | -0.007269000 |
| 6 | -1.137029000 | -2.862250000 | 0.002087000 |
| 1 | 1.313454000 | -5.120686000 | -0.002936000 |
| 1 | -1.380783000 | -5.103052000 | -0.016666000 |
| 6 | -2.482139000 | -2.449562000 | -0.000995000 |
| 6 | 2.449075000 | -2.481671000 | -0.000146000 |
| 6 | -4.262823000 | -0.658102000 | 0.003663000 |
| 6 | -4.253570000 | 0.714328000 | 0.002450000 |
| 6 | -2.861968000 | 1.136654000 | 0.005724000 |
| 6 | -2.877109000 | -1.099071000 | -0.003569000 |
| 1 | -5.120995000 | -1.313276000 | 0.008725000 |
| 1 | -5.102687000 | 1.381022000 | -0.002632000 |
| 6 | -2.449120000 | 2.481627000 | -0.000169000 |
| 6 | 2.877009000 | 1.099039000 | -0.003310000 |
| 6 | 4.262721000 | 0.658093000 | 0.003737000 |
| 6 | 4.253486000 | -0.714335000 | 0.002186000 |
| 6 | 2.861893000 | -1.136686000 | 0.005822000 |
| 1 | 5.120891000 | 1.313271000 | 0.008746000 |
| 1 | 5.102627000 | -1.380995000 | -0.003341000 |
| 6 | -0.658161000 | 4.262620000 | -0.005186000 |
| 6 | 0.714276000 | 4.253709000 | -0.007330000 |
| 6 | 1.136946000 | 2.862218000 | 0.002236000 |
| 6 | -1.098803000 | 2.876836000 | -0.006277000 |
| 1 | -1.313613000 | 5.120592000 | -0.003634000 |
| 1 | 1.380680000 | 5.103026000 | -0.016868000 |
| 6 | 2.482047000 | 2.449529000 | -0.000755000 |
| 6 | -3.552802000 | -3.505480000 | -0.000896000 |
| 6 | -4.336373000 | -3.736451000 | -1.154554000 |
| 6 | -5.338724000 | -4.725525000 | -1.155949000 |
| 6 | -5.576055000 | -5.495298000 | 0.000403000 |
| 6 | -4.800673000 | -5.270954000 | 1.155834000 |
| 6 | -3.795175000 | -4.285182000 | 1.153147000 |
| 1 | -4.155236000 | -3.148049000 | -2.050758000 |
| 1 | -5.928746000 | -4.893551000 | -2.053608000 |
| 1 | -6.351757000 | -6.257061000 | 0.001075000 |
| 1 | -4.977698000 | -5.857718000 | 2.053816000 |
| 1 | -3.204318000 | -4.114064000 | 2.049827000 |
| 6 | 3.505394000 | -3.552190000 | 0.000488000 |
| 6 | 4.275397000 | -3.802934000 | 1.159182000 |
| 6 | 5.262144000 | -4.807531000 | 1.162267000 |
| 6 | 5.496881000 | -5.573586000 | 0.002705000 |
| 6 | 4.736563000 | -5.327940000 | -1.158186000 |
| 6 | 3.746647000 | -4.326398000 | -1.157293000 |
| 1 | 4.096212000 | -3.219053000 | 2.058861000 |
| 1 | 5.841546000 | -4.991114000 | 2.063711000 |
| 1 | 4.912720000 | -5.910830000 | -2.058949000 |
| 1 | 3.165776000 | -4.138547000 | -2.057032000 |
| 6 | 3.552731000 | 3.505425000 | -0.000768000 |
| 6 | 3.795304000 | 4.285020000 | 1.153303000 |
| 6 | 4.800812000 | 5.270790000 | 1.155902000 |
| 6 | 5.576040000 | 5.495189000 | 0.000380000 |
| 6 | 5.338574000 | 4.725447000 | -1.155967000 |
| 6 | 4.336212000 | 3.736392000 | -1.154488000 |
| 1 | 3.204573000 | 4.113869000 | 2.050060000 |
| 1 | 4.977951000 | 5.857513000 | 2.053888000 |
| 1 | 6.351763000 | 6.256930000 | 0.000992000 |
| 1 | 5.928505000 | 4.893486000 | -2.053684000 |
| 1 | 4.154969000 | 3.148015000 | -2.050690000 |
| 6 | -3.505407000 | 3.552208000 | 0.000473000 |
| 6 | -4.275696000 | 3.802690000 | 1.159024000 |
| 6 | -5.262314000 | 4.807420000 | 1.162140000 |

| | | | |
|---|---|---|---|
| 6 | -5.496531000 | 5.573924000 | 0.002766000 |
| 6 | -4.735872000 | 5.328572000 | -1.157963000 |
| 6 | -3.746086000 | 4.326884000 | -1.157094000 |
| 1 | -4.096815000 | 3.218494000 | 2.058558000 |
| 1 | -5.841936000 | 4.990811000 | 2.063483000 |
| 1 | -4.911571000 | 5.911873000 | -2.058550000 |
| 1 | -3.164831000 | 4.139377000 | -2.056663000 |
| 1 | -6.258820000 | 6.349112000 | 0.003796000 |
| 1 | 6.259309000 | -6.348636000 | 0.003699000 |

**[1a]⁺**

| | | | |
|---|---|---|---|
| 7 | 1.683508000 | 1.439232000 | -0.000039000 |
| 7 | -1.213988000 | 1.482679000 | -0.073677000 |
| 7 | 1.639341000 | -1.461160000 | -0.023365000 |
| 7 | -1.257984000 | -1.416413000 | -0.066483000 |
| 6 | 3.062011000 | 1.203284000 | -0.024498000 |
| 6 | 3.741699000 | 2.473832000 | -0.156165000 |
| 6 | 2.785845000 | 3.458205000 | -0.198458000 |
| 6 | 1.490720000 | 2.823558000 | -0.088157000 |
| 1 | 4.811258000 | 2.595374000 | -0.228950000 |
| 1 | 2.938852000 | 4.520270000 | -0.308443000 |
| 6 | 0.266435000 | 3.510795000 | -0.065341000 |
| 6 | 3.704082000 | -0.042540000 | 0.053467000 |
| 6 | -2.250830000 | 3.537132000 | 0.108604000 |
| 6 | -3.236366000 | 2.581166000 | 0.113103000 |
| 6 | -2.599041000 | 1.288890000 | -0.013028000 |
| 6 | -0.978421000 | 2.861022000 | -0.016629000 |
| 1 | -2.368785000 | 4.605480000 | 0.199286000 |
| 1 | -4.300095000 | 2.733759000 | 0.207876000 |
| 6 | -3.282536000 | 0.064060000 | -0.070821000 |
| 6 | 1.401323000 | -2.840126000 | 0.019125000 |
| 6 | 2.668683000 | -3.517023000 | 0.185853000 |
| 6 | 3.653823000 | -2.561817000 | 0.225888000 |
| 6 | 3.021064000 | -1.268433000 | 0.083259000 |
| 1 | 2.783177000 | -4.585703000 | 0.277154000 |
| 1 | 4.713215000 | -2.717992000 | 0.357309000 |
| 6 | -3.309608000 | -2.449443000 | -0.298221000 |
| 6 | -2.352905000 | -3.434262000 | -0.309532000 |
| 6 | -1.062863000 | -2.800156000 | -0.149869000 |
| 6 | -2.635218000 | -1.179990000 | -0.137275000 |
| 1 | -4.376356000 | -2.567366000 | -0.407771000 |
| 1 | -2.501496000 | -4.496002000 | -0.428255000 |
| 6 | 0.159787000 | -3.488600000 | -0.079628000 |
| 6 | 0.286647000 | 5.010879000 | -0.090514000 |
| 6 | 0.831176000 | 5.740409000 | 0.991196000 |
| 6 | 0.842652000 | 7.147310000 | 0.967971000 |
| 6 | 0.320557000 | 7.841879000 | -0.141460000 |
| 6 | -0.218752000 | 7.120701000 | -1.225392000 |
| 6 | 5.203676000 | -0.063581000 | 0.100022000 |
| 6 | 5.945196000 | -0.616017000 | -0.970469000 |
| 6 | 7.346239000 | -0.631325000 | -0.938995000 |
| 6 | 7.999663000 | -0.094868000 | 0.184952000 |
| 6 | 7.291485000 | 0.456117000 | 1.267976000 |
| 6 | 5.891098000 | 0.472264000 | 1.214253000 |
| 1 | 5.426536000 | -1.018167000 | -1.835181000 |
| 1 | 7.921189000 | -1.043645000 | -1.759750000 |
| 1 | 5.329700000 | 0.887578000 | 2.045412000 |
| 6 | 0.138731000 | -4.988733000 | -0.106150000 |
| 6 | 0.702586000 | -5.692733000 | -1.194937000 |
| 6 | 0.679417000 | -7.099432000 | -1.222837000 |
| 6 | 0.103219000 | -7.819606000 | -0.157416000 |
| 6 | -0.455238000 | -7.124091000 | 0.933591000 |
| 6 | -0.442998000 | -5.717196000 | 0.956756000 |
| 6 | -4.782867000 | 0.082689000 | -0.061395000 |
| 6 | -5.503333000 | 0.633311000 | -1.146195000 |
| 6 | -6.905672000 | 0.641906000 | -1.136487000 |
| 6 | -7.611638000 | 0.108902000 | -0.035900000 |
| 6 | -6.898292000 | -0.437150000 | 1.053413000 |
| 6 | -5.495351000 | -0.453656000 | 1.035723000 |
| 1 | -4.967817000 | 1.038221000 | -2.000189000 |
| 1 | -7.460723000 | 1.056088000 | -1.972497000 |
| 1 | -7.440040000 | -0.841900000 | 1.900893000 |
| 1 | -4.952013000 | -0.869359000 | 1.879624000 |
| 6 | -0.241276000 | 5.714053000 | -1.197815000 |
| 6 | -9.101465000 | 0.141273000 | -0.063151000 |
| 8 | -9.784499000 | 0.618213000 | -0.994951000 |
| 1 | 7.824741000 | 0.856679000 | 2.122032000 |

| | | | |
|---|---|---|---|
| 7 | 9.469680000 | -0.109513000 | 0.228786000 |
| 8 | -9.665892000 | -0.416984000 | 1.059602000 |
| 6 | -11.142408000 | -0.440602000 | 1.143534000 |
| 1 | -11.555733000 | -1.006730000 | 0.304062000 |
| 1 | -11.360642000 | -0.931749000 | 2.091819000 |
| 1 | -11.533755000 | 0.580627000 | 1.131969000 |
| 1 | 1.142724000 | -5.142915000 | -2.023061000 |
| 1 | 1.107751000 | -7.628204000 | -2.070124000 |
| 1 | -0.896856000 | -7.672287000 | 1.761495000 |
| 1 | -0.869187000 | -5.187027000 | 1.804740000 |
| 1 | -0.652975000 | 5.163557000 | -2.040008000 |
| 1 | 1.228413000 | 5.211101000 | 1.853635000 |
| 1 | -0.619187000 | 7.648683000 | -2.086695000 |
| 1 | 1.255444000 | 7.696216000 | 1.810146000 |
| 1 | 0.333446000 | 8.928447000 | -0.160921000 |
| 1 | 0.089731000 | -8.906165000 | -0.176973000 |
| 79 | 0.212761000 | 0.011064000 | -0.040851000 |
| 8 | 10.057143000 | 0.386905000 | 1.253527000 |
| 8 | 10.107381000 | -0.617910000 | -0.759386000 |

**1a**

| | | | |
|---|---|---|---|
| 7 | 1.732986000 | 1.494319000 | 0.029183000 |
| 7 | -1.269620000 | 1.530035000 | -0.071475000 |
| 7 | 1.697942000 | -1.509762000 | -0.035824000 |
| 7 | -1.306166000 | -1.473598000 | -0.016306000 |
| 6 | 3.098003000 | 1.246144000 | -0.000968000 |
| 6 | 3.781350000 | 2.534807000 | -0.098252000 |
| 6 | 2.818395000 | 3.518418000 | -0.113443000 |
| 6 | 1.514506000 | 2.865902000 | -0.026139000 |
| 1 | 4.849851000 | 2.674595000 | -0.169425000 |
| 1 | 2.977314000 | 4.583311000 | -0.195435000 |
| 6 | 0.255219000 | 3.524220000 | -0.020773000 |
| 6 | 3.722563000 | -0.031745000 | 0.024095000 |
| 6 | -2.308647000 | 3.577922000 | 0.073794000 |
| 6 | -3.294165000 | 2.616444000 | 0.059172000 |
| 6 | -2.641026000 | 1.313138000 | -0.038940000 |
| 6 | -1.020437000 | 2.895151000 | -0.015063000 |
| 1 | -2.442534000 | 4.646313000 | 0.155092000 |
| 1 | -4.359793000 | 2.777406000 | 0.128257000 |
| 6 | -3.296935000 | 0.052161000 | -0.068765000 |
| 6 | 1.445397000 | -2.875762000 | 0.011198000 |
| 6 | 2.730471000 | -3.559421000 | 0.132040000 |
| 6 | 3.716739000 | -2.599143000 | 0.144431000 |
| 6 | 3.067211000 | -1.294350000 | 0.031116000 |
| 1 | 2.861647000 | -4.627948000 | 0.215907000 |
| 1 | 4.779305000 | -2.764616000 | 0.243644000 |
| 6 | -3.352010000 | -2.511438000 | -0.199021000 |
| 6 | -2.389609000 | -3.496145000 | -0.189658000 |
| 6 | -1.088213000 | -2.844267000 | -0.067434000 |
| 6 | -2.670707000 | -1.224095000 | -0.083283000 |
| 1 | -4.419063000 | -2.646935000 | -0.295495000 |
| 1 | -2.546606000 | -4.561023000 | -0.275544000 |
| 6 | 0.171375000 | -3.503670000 | -0.028716000 |
| 6 | 0.272576000 | 5.028264000 | -0.020700000 |
| 6 | 0.796662000 | 5.743219000 | 1.081377000 |
| 6 | 0.809086000 | 7.150802000 | 1.083919000 |
| 6 | 0.306083000 | 7.866014000 | -0.021355000 |
| 6 | -0.213871000 | 7.162316000 | -1.126212000 |
| 6 | 5.223196000 | -0.049741000 | 0.044869000 |
| 6 | 5.952313000 | -0.637269000 | -1.018331000 |
| 6 | 7.352758000 | -0.654352000 | -1.011076000 |
| 6 | 8.028829000 | -0.084129000 | 0.084117000 |
| 6 | 7.336613000 | 0.502910000 | 1.160159000 |
| 6 | 5.936470000 | 0.520236000 | 1.128182000 |
| 1 | 5.417902000 | -1.063497000 | -1.861526000 |
| 1 | 7.912791000 | -1.092391000 | -1.828952000 |
| 1 | 5.389288000 | 0.959833000 | 1.956202000 |
| 6 | 0.152619000 | -5.007724000 | -0.030417000 |
| 6 | 0.684628000 | -5.734245000 | -1.121027000 |
| 6 | 0.662540000 | -7.141736000 | -1.125344000 |
| 6 | 0.116576000 | -7.845378000 | -0.033114000 |
| 6 | -0.411400000 | -7.130166000 | 1.060489000 |
| 6 | -0.397734000 | -5.722609000 | 1.058838000 |
| 6 | -4.799646000 | 0.069857000 | -0.085940000 |
| 6 | -5.503127000 | 0.622666000 | -1.182431000 |
| 6 | -6.905121000 | 0.635209000 | -1.200979000 |
| 6 | -7.635393000 | 0.102229000 | -0.115575000 |

| | | | |
|---|---|---|---|
| 6 | -6.942321000 | -0.447121000 | 0.985955000 |
| 6 | -5.539458000 | -0.465652000 | 0.994755000 |
| 1 | -4.949510000 | 1.027131000 | -2.024980000 |
| 1 | -7.442180000 | 1.052026000 | -2.047614000 |
| 1 | -7.500550000 | -0.851664000 | 1.823028000 |
| 1 | -5.013012000 | -0.882047000 | 1.848873000 |
| 6 | -0.234871000 | 5.754866000 | -1.122960000 |
| 6 | -9.121860000 | 0.138496000 | -0.171227000 |
| 8 | -9.789959000 | 0.617746000 | -1.113901000 |
| 1 | 7.884012000 | 0.927459000 | 1.993556000 |
| 7 | 9.494453000 | -0.102042000 | 0.104510000 |
| 8 | -9.711807000 | -0.419864000 | 0.941059000 |
| 6 | -11.188738000 | -0.436500000 | 0.994262000 |
| 1 | -11.588405000 | -1.003120000 | 0.148331000 |
| 1 | -11.429297000 | -0.923893000 | 1.939246000 |
| 1 | -11.576255000 | 0.586136000 | 0.971698000 |
| 1 | 1.100879000 | -5.197791000 | -1.970065000 |
| 1 | 1.067951000 | -7.684103000 | -1.975816000 |
| 1 | -0.830504000 | -7.663685000 | 1.909926000 |
| 1 | -0.800519000 | -5.177416000 | 1.908803000 |
| 1 | -0.631123000 | 5.218500000 | -1.981553000 |
| 1 | 1.179713000 | 5.198040000 | 1.940422000 |
| 1 | -0.600048000 | 7.704650000 | -1.985605000 |
| 1 | 1.207851000 | 7.684298000 | 1.943102000 |
| 1 | 0.318919000 | 8.953059000 | -0.021661000 |
| 1 | 0.102789000 | -8.932412000 | -0.034165000 |
| 79 | 0.213766000 | 0.010246000 | -0.023415000 |
| 8 | 10.103792000 | 0.422237000 | 1.104791000 |
| 8 | 10.118365000 | -0.641454000 | -0.878558000 |

**1a''** (constrained)

| | | | |
|---|---|---|---|
| 7 | 1.692306000 | 1.442567000 | 0.011539000 |
| 7 | -1.224019000 | 1.485671000 | -0.058579000 |
| 7 | 1.649212000 | -1.459449000 | -0.027216000 |
| 7 | -1.267361000 | -1.414976000 | -0.043608000 |
| 6 | 3.067527000 | 1.208831000 | -0.083154000 |
| 6 | 3.724118000 | 2.470858000 | -0.323256000 |
| 6 | 2.761528000 | 3.457120000 | -0.329564000 |
| 6 | 1.485974000 | 2.828891000 | -0.113039000 |
| 1 | 4.782416000 | 2.591699000 | -0.490241000 |
| 1 | 2.905296000 | 4.513546000 | -0.494754000 |
| 6 | 0.257793000 | 3.512683000 | -0.034192000 |
| 6 | 3.731291000 | -0.039524000 | 0.030213000 |
| 6 | -2.249886000 | 3.532136000 | 0.250507000 |
| 6 | -3.236964000 | 2.575014000 | 0.238456000 |
| 6 | -2.605668000 | 1.289403000 | 0.033512000 |
| 6 | -0.983420000 | 2.867579000 | 0.052796000 |
| 1 | -2.366589000 | 4.595044000 | 0.395074000 |
| 1 | -4.296886000 | 2.722305000 | 0.376101000 |
| 6 | -3.292770000 | 0.065727000 | -0.066865000 |
| 6 | 1.397808000 | -2.839583000 | 0.079338000 |
| 6 | 2.645738000 | -3.507133000 | 0.335613000 |
| 6 | 3.636703000 | -2.550051000 | 0.371262000 |
| 6 | 3.026825000 | -1.267503000 | 0.116456000 |
| 1 | 2.751736000 | -4.568426000 | 0.498200000 |
| 1 | 4.684190000 | -2.703630000 | 0.575345000 |
| 6 | -3.305100000 | -2.442188000 | -0.387528000 |
| 6 | -2.346482000 | -3.427970000 | -0.389296000 |
| 6 | -1.065433000 | -2.802824000 | -0.159933000 |
| 6 | -2.640226000 | -1.177382000 | -0.160579000 |
| 1 | -4.365988000 | -2.556577000 | -0.547316000 |
| 1 | -2.491400000 | -4.485780000 | -0.545250000 |
| 6 | 0.153103000 | -3.485555000 | -0.044609000 |
| 6 | 0.281340000 | 5.012871000 | -0.040723000 |
| 6 | 0.916263000 | 5.726618000 | 1.002232000 |
| 6 | 0.932571000 | 7.133623000 | 0.998961000 |
| 6 | 0.325356000 | 7.846915000 | -0.054041000 |
| 6 | -0.303557000 | 7.143182000 | -1.100519000 |
| 6 | 5.210689000 | -0.061261000 | 0.057614000 |
| 6 | 5.953293000 | -0.916168000 | -0.807900000 |
| 6 | 7.347476000 | -0.932172000 | -0.800014000 |
| 6 | 8.052963000 | -0.104125000 | 0.112615000 |
| 6 | 7.338037000 | 0.746374000 | 0.996819000 |
| 6 | 5.944762000 | 0.772461000 | 0.950632000 |
| 1 | 5.422966000 | -1.539313000 | -1.522280000 |
| 1 | 7.905608000 | -1.560379000 | -1.483994000 |
| 1 | 5.406281000 | 1.412548000 | 1.643615000 |

| | | | |
|---|---|---|---|
| 6 | 0.133203000 | -4.985832000 | -0.052589000 |
| 6 | 0.776586000 | -5.707159000 | -1.085088000 |
| 6 | 0.753444000 | -7.114044000 | -1.095980000 |
| 6 | 0.097809000 | -7.819960000 | -0.067327000 |
| 6 | -0.539722000 | -7.108817000 | 0.968878000 |
| 6 | -0.527190000 | -5.701625000 | 0.972889000 |
| 6 | -4.791357000 | 0.085777000 | -0.074157000 |
| 6 | -5.501225000 | 0.736558000 | -1.111416000 |
| 6 | -6.903177000 | 0.749360000 | -1.120551000 |
| 6 | -7.626698000 | 0.118414000 | -0.084318000 |
| 6 | -6.927405000 | -0.528948000 | 0.958404000 |
| 6 | -5.524742000 | -0.547316000 | 0.957455000 |
| 1 | -4.953339000 | 1.216839000 | -1.917221000 |
| 1 | -7.445372000 | 1.240505000 | -1.922855000 |
| 1 | -7.481071000 | -1.008049000 | 1.758268000 |
| 1 | -4.993839000 | -1.040133000 | 1.767102000 |
| 6 | -0.330648000 | 5.736241000 | -1.090684000 |
| 6 | -9.113958000 | 0.157256000 | -0.127913000 |
| 8 | -9.787182000 | 0.712089000 | -1.024096000 |
| 1 | 7.887927000 | 1.358560000 | 1.701672000 |
| 7 | 9.469817000 | -0.126577000 | 0.141075000 |
| 8 | -9.695921000 | -0.491653000 | 0.937894000 |
| 6 | -11.172710000 | -0.515885000 | 0.999223000 |
| 1 | -11.576533000 | -1.015653000 | 0.114078000 |
| 1 | -11.405913000 | -1.076469000 | 1.904593000 |
| 1 | -11.562218000 | 0.504454000 | 1.059216000 |
| 1 | 1.279788000 | -5.168626000 | -1.884102000 |
| 1 | 1.243785000 | -7.654411000 | -1.901521000 |
| 1 | -1.042292000 | -7.645179000 | 1.769578000 |
| 1 | -1.014328000 | -5.159094000 | 1.779169000 |
| 1 | -0.811148000 | 5.199158000 | -1.904557000 |
| 1 | 1.381820000 | 5.182646000 | 1.820148000 |
| 1 | -0.769113000 | 7.685019000 | -1.919683000 |
| 1 | 1.415687000 | 7.668383000 | 1.812569000 |
| 1 | 0.342536000 | 8.933749000 | -0.059253000 |
| 1 | 0.084739000 | -8.906851000 | -0.072819000 |
| 79 | 0.212468000 | 0.013429000 | -0.029464000 |
| 8 | 10.128775000 | 0.657460000 | 1.019062000 |
| 8 | 10.138507000 | -0.931973000 | -0.709803000 |

**[1a···$PF_6$]**

| | | | |
|---|---|---|---|
| 7 | -1.429033000 | -1.124024000 | -0.853684000 |
| 7 | 1.457642000 | -1.286153000 | -0.689884000 |
| 7 | -1.290145000 | 1.752916000 | -0.525890000 |
| 7 | 1.600518000 | 1.593804000 | -0.373952000 |
| 6 | -2.791782000 | -0.833456000 | -0.928504000 |
| 6 | -3.506243000 | -2.058290000 | -1.219189000 |
| 6 | -2.585329000 | -3.073229000 | -1.282336000 |
| 6 | -1.280420000 | -2.500998000 | -1.030997000 |
| 1 | -4.573387000 | -2.128733000 | -1.361571000 |
| 1 | -2.765558000 | -4.117671000 | -1.482823000 |
| 6 | -0.091507000 | -3.241025000 | -0.951364000 |
| 6 | -3.396995000 | 0.417658000 | -0.741201000 |
| 6 | 2.406416000 | -3.389406000 | -0.628355000 |
| 6 | 3.424230000 | -2.478575000 | -0.488611000 |
| 6 | 2.841509000 | -1.155019000 | -0.531863000 |
| 6 | 1.169390000 | -2.652917000 | -0.766346000 |
| 1 | 2.478783000 | -4.465625000 | -0.623300000 |
| 1 | 4.474562000 | -2.681523000 | -0.348876000 |
| 6 | 3.568519000 | 0.040878000 | -0.424689000 |
| 6 | -1.010319000 | 3.106599000 | -0.310038000 |
| 6 | -2.263715000 | 3.812754000 | -0.155513000 |
| 6 | -3.279588000 | 2.899109000 | -0.288078000 |
| 6 | -2.679927000 | 1.604863000 | -0.531765000 |
| 1 | -2.348964000 | 4.868653000 | 0.047912000 |
| 1 | -4.340171000 | 3.080765000 | -0.209520000 |
| 6 | 3.696203000 | 2.561297000 | -0.357822000 |
| 6 | 2.775751000 | 3.579264000 | -0.311557000 |
| 6 | 1.457422000 | 2.984167000 | -0.308881000 |
| 6 | 2.970187000 | 1.310368000 | -0.387538000 |
| 1 | 4.771326000 | 2.647099000 | -0.385092000 |
| 1 | 2.966997000 | 4.640720000 | -0.293622000 |
| 6 | 0.256021000 | 3.709240000 | -0.244165000 |
| 6 | -0.181516000 | -4.737021000 | -1.018340000 |
| 6 | -0.805921000 | -5.446320000 | 0.033944000 |
| 6 | -0.888453000 | -6.850493000 | -0.008693000 |
| 6 | -0.357735000 | -7.559548000 | -1.105197000 |

| | | | |
|---|---|---|---|
| 6 | 0.261838000 | -6.856531000 | -2.157821000 |
| 6 | -4.895775000 | 0.479493000 | -0.728054000 |
| 6 | -5.601943000 | 1.148724000 | -1.754147000 |
| 6 | -7.002323000 | 1.201702000 | -1.741242000 |
| 6 | -7.688220000 | 0.584085000 | -0.679353000 |
| 6 | -7.012655000 | -0.082901000 | 0.359269000 |
| 6 | -5.612340000 | -0.135583000 | 0.326381000 |
| 1 | -5.056646000 | 1.613518000 | -2.569906000 |
| 1 | -7.552891000 | 1.703530000 | -2.528154000 |
| 1 | -5.066656000 | -0.635706000 | 1.120796000 |
| 6 | 0.329393000 | 5.200439000 | -0.091303000 |
| 6 | -0.118693000 | 6.050041000 | -1.128643000 |
| 6 | -0.046151000 | 7.448374000 | -0.987299000 |
| 6 | 0.465207000 | 8.014808000 | 0.197430000 |
| 6 | 0.908104000 | 7.173711000 | 1.237797000 |
| 6 | 0.845690000 | 5.775341000 | 1.092530000 |
| 6 | 5.064361000 | -0.041218000 | -0.339268000 |
| 6 | 5.823042000 | -0.499428000 | -1.441010000 |
| 6 | 7.221379000 | -0.569812000 | -1.361964000 |
| 6 | 7.885372000 | -0.192465000 | -0.174113000 |
| 6 | 7.133511000 | 0.259874000 | 0.932379000 |
| 6 | 5.735157000 | 0.338671000 | 0.845794000 |
| 1 | 5.319987000 | -0.785465000 | -2.360411000 |
| 1 | 7.805476000 | -0.913325000 | -2.210192000 |
| 1 | 7.642016000 | 0.543908000 | 1.846923000 |
| 1 | 5.161366000 | 0.680236000 | 1.702601000 |
| 6 | 0.354756000 | -5.452650000 | -2.112062000 |
| 6 | 9.371551000 | -0.285631000 | -0.129979000 |
| 8 | 10.088318000 | -0.676634000 | -1.076865000 |
| 1 | -7.571160000 | -0.542483000 | 1.166368000 |
| 7 | -9.156529000 | 0.637746000 | -0.655901000 |
| 8 | 9.894360000 | 0.109379000 | 1.079485000 |
| 6 | 11.363468000 | 0.056786000 | 1.238896000 |
| 1 | 11.842957000 | 0.714391000 | 0.508165000 |
| 1 | 11.546806000 | 0.403301000 | 2.255974000 |
| 1 | 11.718034000 | -0.969075000 | 1.104291000 |
| 1 | -0.508788000 | 5.619151000 | -2.047414000 |
| 1 | -0.386340000 | 8.089708000 | -1.796127000 |
| 1 | 1.298106000 | 7.602225000 | 2.157250000 |
| 1 | 1.180977000 | 5.131830000 | 1.901937000 |
| 1 | 0.828810000 | -4.914518000 | -2.929328000 |
| 1 | -1.207131000 | -4.898304000 | 0.882743000 |
| 1 | 0.669917000 | -7.396174000 | -3.008495000 |
| 1 | -1.362638000 | -7.386349000 | 0.809416000 |
| 1 | -0.425510000 | -8.643936000 | -1.138705000 |
| 1 | 0.516818000 | 9.094786000 | 0.308634000 |
| 79 | 0.082210000 | 0.232152000 | -0.597942000 |
| 8 | -9.777169000 | 0.073522000 | 0.313067000 |
| 8 | -9.764491000 | 1.247106000 | -1.606401000 |
| 9 | -1.183600000 | -0.218353000 | 2.246133000 |
| 9 | -2.419394000 | -0.215968000 | 4.356402000 |
| 9 | -0.401018000 | -1.590865000 | 4.114978000 |
| 15 | -1.894714000 | -1.441917000 | 3.256625000 |
| 9 | -3.385273000 | -1.286127000 | 2.377259000 |
| 9 | -2.608373000 | -2.659244000 | 4.252813000 |
| 9 | -1.371438000 | -2.663624000 | 2.139832000 |

**[1a···PF$_6$]$^-$**

| | | | |
|---|---|---|---|
| 7 | -1.408606000 | -0.958248000 | -0.936870000 |
| 7 | 1.574280000 | -1.249423000 | -0.769617000 |
| 7 | -1.153856000 | 1.999993000 | -0.484018000 |
| 7 | 1.824006000 | 1.695934000 | -0.225105000 |
| 6 | -2.742490000 | -0.592425000 | -1.028221000 |
| 6 | -3.506112000 | -1.784512000 | -1.392432000 |
| 6 | -2.622532000 | -2.834509000 | -1.481651000 |
| 6 | -1.289410000 | -2.320312000 | -1.178519000 |
| 1 | -4.571570000 | -1.817779000 | -1.564711000 |
| 1 | -2.851355000 | -3.858522000 | -1.735796000 |
| 6 | -0.094486000 | -3.086710000 | -1.139420000 |
| 6 | -3.275552000 | 0.708867000 | -0.819199000 |
| 6 | 2.434496000 | -3.382754000 | -0.811491000 |
| 6 | 3.486907000 | -2.518684000 | -0.606073000 |
| 6 | 2.947035000 | -1.161329000 | -0.580521000 |
| 6 | 1.218155000 | -2.582303000 | -0.925074000 |
| 1 | 2.476994000 | -4.460472000 | -0.861141000 |
| 1 | 4.524192000 | -2.781205000 | -0.459428000 |
| 6 | 3.694122000 | 0.029077000 | -0.366560000 |

| | | | |
|---|---|---|---|
| 6 | -0.808228000 | 3.311685000 | -0.181952000 |
| 6 | -2.046110000 | 4.072096000 | -0.031036000 |
| 6 | -3.096702000 | 3.209686000 | -0.250060000 |
| 6 | -2.535473000 | 1.892929000 | -0.546618000 |
| 1 | -2.106360000 | 5.117623000 | 0.232186000 |
| 1 | -4.149188000 | 3.443909000 | -0.190236000 |
| 6 | 3.949223000 | 2.569488000 | -0.098375000 |
| 6 | 3.064718000 | 3.620991000 | -0.003806000 |
| 6 | 1.713269000 | 3.072521000 | -0.077109000 |
| 6 | 3.167318000 | 1.342939000 | -0.232380000 |
| 1 | 5.027746000 | 2.625612000 | -0.096680000 |
| 1 | 3.306729000 | 4.669266000 | 0.089401000 |
| 6 | 0.507059000 | 3.824647000 | -0.022483000 |
| 6 | -0.238612000 | -4.574091000 | -1.303900000 |
| 6 | -0.957517000 | -5.325732000 | -0.343708000 |
| 6 | -1.089867000 | -6.720179000 | -0.484135000 |
| 6 | -0.515771000 | -7.382219000 | -1.588320000 |
| 6 | 0.196756000 | -6.639352000 | -2.551069000 |
| 6 | -4.769688000 | 0.837027000 | -0.858678000 |
| 6 | -5.403748000 | 1.659811000 | -1.821994000 |
| 6 | -6.798308000 | 1.779117000 | -1.863164000 |
| 6 | -7.563651000 | 1.071365000 | -0.916272000 |
| 6 | -6.964478000 | 0.250126000 | 0.058565000 |
| 6 | -5.568591000 | 0.133873000 | 0.078283000 |
| 1 | -4.800864000 | 2.190311000 | -2.552582000 |
| 1 | -7.287546000 | 2.397679000 | -2.606451000 |
| 1 | -5.087544000 | -0.484323000 | 0.830422000 |
| 6 | 0.636648000 | 5.301347000 | 0.231583000 |
| 6 | 0.242339000 | 6.240810000 | -0.749518000 |
| 6 | 0.366279000 | 7.622892000 | -0.512436000 |
| 6 | 0.878410000 | 8.088274000 | 0.715354000 |
| 6 | 1.269559000 | 7.160122000 | 1.701225000 |
| 6 | 1.153869000 | 5.778403000 | 1.458690000 |
| 6 | 5.186425000 | -0.116900000 | -0.269895000 |
| 6 | 5.939605000 | -0.574932000 | -1.377349000 |
| 6 | 7.332635000 | -0.708195000 | -1.291470000 |
| 6 | 8.004094000 | -0.395629000 | -0.088309000 |
| 6 | 7.260819000 | 0.056780000 | 1.024837000 |
| 6 | 5.868204000 | 0.198278000 | 0.929394000 |
| 1 | 5.432031000 | -0.811143000 | -2.308256000 |
| 1 | 7.907590000 | -1.051018000 | -2.146449000 |
| 1 | 7.772280000 | 0.290625000 | 1.952101000 |
| 1 | 5.301927000 | 0.537488000 | 1.792041000 |
| 6 | 0.338265000 | -5.246145000 | -2.406191000 |
| 6 | 9.481793000 | -0.556410000 | -0.035283000 |
| 8 | 10.192995000 | -0.952328000 | -0.985528000 |
| 1 | -7.579287000 | -0.275369000 | 0.780153000 |
| 7 | -9.022527000 | 1.193889000 | -0.947751000 |
| 8 | 10.013390000 | -0.220593000 | 1.190650000 |
| 6 | 11.476108000 | -0.348532000 | 1.356876000 |
| 1 | 11.992974000 | 0.305716000 | 0.648806000 |
| 1 | 11.668973000 | -0.040887000 | 2.384799000 |
| 1 | 11.782946000 | -1.385845000 | 1.194532000 |
| 1 | -0.147514000 | 5.888702000 | -1.701368000 |
| 1 | 0.065470000 | 8.330363000 | -1.281226000 |
| 1 | 1.660298000 | 7.508494000 | 2.654081000 |
| 1 | 1.449464000 | 5.068153000 | 2.226764000 |
| 1 | 0.883065000 | -4.677511000 | -3.155989000 |
| 1 | -1.397596000 | -4.817379000 | 0.510691000 |
| 1 | 0.638723000 | -7.139726000 | -3.409298000 |
| 1 | -1.637836000 | -7.285616000 | 0.265657000 |
| 1 | -0.622285000 | -8.458856000 | -1.697081000 |
| 1 | 0.970333000 | 9.155556000 | 0.901030000 |
| 79 | 0.206409000 | 0.368825000 | -0.589991000 |
| 8 | -9.560272000 | 1.955248000 | -1.831592000 |
| 8 | -9.717608000 | 0.536739000 | -0.091726000 |
| 9 | -1.482015000 | -1.162928000 | 2.789164000 |
| 9 | -3.165693000 | -1.156573000 | 4.568230000 |
| 15 | -2.702366000 | -2.268633000 | 3.325707000 |
| 9 | -2.243984000 | -3.382349000 | 2.076669000 |
| 9 | -3.927729000 | -3.372634000 | 3.852162000 |
| 9 | -1.566141000 | -3.008003000 | 4.400927000 |
| 9 | -3.845045000 | -1.529389000 | 2.245735000 |

**[1a''⋯$PF_6$]$^-$**

| | | | |
|---|---|---|---|
| 7 | -1.602519000 | -1.173078000 | -0.710296000 |
| 7 | 1.315052000 | -1.242623000 | -0.715067000 |

| | | | |
|---|---|---|---|
| 7 | -1.534413000 | 1.706021000 | -0.397275000 |
| 7 | 1.381780000 | 1.632807000 | -0.355534000 |
| 6 | -2.969046000 | -0.919118000 | -0.851882000 |
| 6 | -3.622058000 | -2.157429000 | -1.204589000 |
| 6 | -2.669025000 | -3.152969000 | -1.211188000 |
| 6 | -1.401255000 | -2.549985000 | -0.901642000 |
| 1 | -4.670965000 | -2.258914000 | -1.431513000 |
| 1 | -2.811859000 | -4.199055000 | -1.434224000 |
| 6 | -0.183298000 | -3.249333000 | -0.827401000 |
| 6 | -3.626351000 | 0.323637000 | -0.664737000 |
| 6 | 2.326988000 | -3.315216000 | -0.592979000 |
| 6 | 3.322983000 | -2.367459000 | -0.560105000 |
| 6 | 2.697604000 | -1.064519000 | -0.636501000 |
| 6 | 1.063966000 | -2.626810000 | -0.706047000 |
| 1 | 2.435793000 | -4.387199000 | -0.534459000 |
| 1 | 4.384739000 | -2.532240000 | -0.461539000 |
| 6 | 3.393951000 | 0.159234000 | -0.615237000 |
| 6 | -1.279742000 | 3.050625000 | -0.067907000 |
| 6 | -2.536203000 | 3.692774000 | 0.207881000 |
| 6 | -3.534894000 | 2.760020000 | 0.026711000 |
| 6 | -2.918278000 | 1.518543000 | -0.373412000 |
| 1 | -2.645417000 | 4.718338000 | 0.524661000 |
| 1 | -4.592867000 | 2.898233000 | 0.182443000 |
| 6 | 3.423837000 | 2.688534000 | -0.550163000 |
| 6 | 2.473834000 | 3.667375000 | -0.375213000 |
| 6 | 1.189393000 | 3.021899000 | -0.243762000 |
| 6 | 2.751183000 | 1.407352000 | -0.520203000 |
| 1 | 4.483976000 | 2.821431000 | -0.699989000 |
| 1 | 2.625913000 | 4.735563000 | -0.354333000 |
| 6 | -0.025844000 | 3.688948000 | -0.039146000 |
| 6 | -0.234558000 | -4.748330000 | -0.867875000 |
| 6 | -0.826884000 | -5.458610000 | 0.202006000 |
| 6 | -0.878775000 | -6.864851000 | 0.179055000 |
| 6 | -0.349150000 | -7.577280000 | -0.915978000 |
| 6 | 0.239347000 | -6.874414000 | -1.986409000 |
| 6 | -5.102148000 | 0.381448000 | -0.757760000 |
| 6 | -5.745364000 | 1.354220000 | -1.577963000 |
| 6 | -7.133786000 | 1.412398000 | -1.693916000 |
| 6 | -7.935257000 | 0.501442000 | -0.958519000 |
| 6 | -7.324050000 | -0.464201000 | -0.117750000 |
| 6 | -5.932962000 | -0.523637000 | -0.035070000 |
| 1 | -5.137937000 | 2.041480000 | -2.159938000 |
| 1 | -7.611702000 | 2.135316000 | -2.344487000 |
| 1 | -5.474579000 | -1.247415000 | 0.631980000 |
| 6 | 0.011644000 | 5.168685000 | 0.208683000 |
| 6 | -0.576217000 | 6.070481000 | -0.708375000 |
| 6 | -0.533605000 | 7.457698000 | -0.475379000 |
| 6 | 0.087712000 | 7.962671000 | 0.684567000 |
| 6 | 0.671216000 | 7.070486000 | 1.606241000 |
| 6 | 0.638509000 | 5.683958000 | 1.366819000 |
| 6 | 4.890573000 | 0.126861000 | -0.672737000 |
| 6 | 5.556728000 | -0.406164000 | -1.802739000 |
| 6 | 6.956961000 | -0.432695000 | -1.863645000 |
| 6 | 7.725067000 | 0.065873000 | -0.787753000 |
| 6 | 7.070346000 | 0.592122000 | 0.348277000 |
| 6 | 5.668968000 | 0.624248000 | 0.399790000 |
| 1 | 4.974316000 | -0.784214000 | -2.638303000 |
| 1 | 7.464246000 | -0.833174000 | -2.736170000 |
| 1 | 7.657329000 | 0.966615000 | 1.179638000 |
| 1 | 5.172964000 | 1.019285000 | 1.281722000 |
| 6 | 0.301044000 | -5.468159000 | -1.959592000 |
| 6 | 9.208094000 | 0.018497000 | -0.889734000 |
| 8 | 9.843397000 | -0.431987000 | -1.869032000 |
| 1 | -7.947980000 | -1.136472000 | 0.459436000 |
| 7 | -9.356217000 | 0.558010000 | -1.063732000 |
| 8 | 9.837224000 | 0.530976000 | 0.223503000 |
| 6 | 11.314881000 | 0.529865000 | 0.232661000 |
| 1 | 11.695630000 | 1.140067000 | -0.591561000 |
| 1 | 11.589178000 | 0.960358000 | 1.195912000 |
| 1 | 11.689809000 | -0.493464000 | 0.139880000 |
| 1 | -1.052648000 | 5.687805000 | -1.607429000 |
| 1 | -0.982465000 | 8.138539000 | -1.194124000 |
| 1 | 1.147238000 | 7.450031000 | 2.506726000 |
| 1 | 1.083239000 | 4.999941000 | 2.085071000 |
| 1 | 0.750522000 | -4.929713000 | -2.790463000 |
| 1 | -1.222799000 | -4.907731000 | 1.050812000 |
| 1 | 0.646868000 | -7.415924000 | -2.836438000 |
| 1 | -1.327777000 | -7.400122000 | 1.011880000 |

| | | | |
|---|---|---|---|
| 1 | -0.393697000 | -8.663428000 | -0.934527000 |
| 1 | 0.115713000 | 9.033880000 | 0.867608000 |
| 79 | -0.110109000 | 0.228086000 | -0.531249000 |
| 8 | -10.086340000 | -0.308020000 | -0.395438000 |
| 8 | -9.908558000 | 1.475070000 | -1.828487000 |
| 9 | 0.402646000 | -0.783384000 | 2.512901000 |
| 9 | -0.508358000 | -0.303760000 | 4.734133000 |
| 9 | 0.686725000 | -2.404189000 | 4.324921000 |
| 15 | -0.669873000 | -1.605949000 | 3.605933000 |
| 9 | -2.022804000 | -0.805203000 | 2.875550000 |
| 9 | -1.737916000 | -2.425590000 | 4.691842000 |
| 9 | -0.829858000 | -2.906311000 | 2.467771000 |

**[2a]$^+$**

| | | | |
|---|---|---|---|
| 7 | 2.015073000 | 1.424382000 | 0.010459000 |
| 7 | -0.887364000 | 1.484436000 | -0.067041000 |
| 7 | 1.956993000 | -1.472878000 | -0.031519000 |
| 7 | -0.946329000 | -1.413735000 | -0.080577000 |
| 6 | 3.392851000 | 1.178818000 | -0.046114000 |
| 6 | 4.069520000 | 2.438967000 | -0.248810000 |
| 6 | 3.117227000 | 3.429668000 | -0.284852000 |
| 6 | 1.826059000 | 2.806872000 | -0.111166000 |
| 1 | 5.135404000 | 2.546446000 | -0.372208000 |
| 1 | 3.271512000 | 4.486366000 | -0.437117000 |
| 6 | 0.603902000 | 3.502406000 | -0.063687000 |
| 6 | 4.042051000 | -0.067080000 | 0.074791000 |
| 6 | -1.908731000 | 3.540520000 | 0.178057000 |
| 6 | -2.899302000 | 2.589626000 | 0.181159000 |
| 6 | -2.272013000 | 1.296609000 | 0.013390000 |
| 6 | -0.642194000 | 2.861579000 | 0.011839000 |
| 1 | -2.019347000 | 4.607237000 | 0.294775000 |
| 1 | -3.960077000 | 2.745609000 | 0.300981000 |
| 6 | -2.962872000 | 0.076985000 | -0.071525000 |
| 6 | 1.705173000 | -2.848489000 | 0.035158000 |
| 6 | 2.955209000 | -3.529700000 | 0.276922000 |
| 6 | 3.945655000 | -2.578886000 | 0.343988000 |
| 6 | 3.336592000 | -1.286578000 | 0.129151000 |
| 1 | 3.055374000 | -4.595998000 | 0.405744000 |
| 1 | 4.994155000 | -2.734938000 | 0.541443000 |
| 6 | -2.997983000 | -2.430014000 | -0.375237000 |
| 6 | -2.046082000 | -3.419483000 | -0.395421000 |
| 6 | -0.755650000 | -2.797586000 | -0.193401000 |
| 6 | -2.320681000 | -1.168302000 | -0.168233000 |
| 1 | -4.063107000 | -2.538925000 | -0.508037000 |
| 1 | -2.198119000 | -4.476867000 | -0.545442000 |
| 6 | 0.460434000 | -3.490925000 | -0.098596000 |
| 6 | 0.634584000 | 5.001872000 | -0.092048000 |
| 6 | 1.239235000 | 5.728755000 | 0.959569000 |
| 6 | 1.260821000 | 7.135407000 | 0.934503000 |
| 6 | 0.689143000 | 7.833732000 | -0.147926000 |
| 6 | 0.090234000 | 7.116070000 | -1.202467000 |
| 6 | 5.530267000 | -0.092720000 | 0.146591000 |
| 6 | 6.302019000 | -0.844296000 | -0.775606000 |
| 6 | 7.699420000 | -0.865265000 | -0.713826000 |
| 6 | 8.390529000 | -0.143232000 | 0.299985000 |
| 6 | 7.621292000 | 0.608110000 | 1.232709000 |
| 6 | 6.225703000 | 0.636181000 | 1.144788000 |
| 1 | 5.802633000 | -1.394890000 | -1.569154000 |
| 1 | 8.265108000 | -1.434762000 | -1.447640000 |
| 1 | 5.664125000 | 1.207569000 | 1.879935000 |
| 6 | 0.434999000 | -4.990528000 | -0.129025000 |
| 6 | 1.057035000 | -5.695297000 | -1.185595000 |
| 6 | 1.030299000 | -7.101712000 | -1.218609000 |
| 6 | 0.392661000 | -7.822483000 | -0.189084000 |
| 6 | -0.223188000 | -7.127227000 | 0.870741000 |
| 6 | -0.207257000 | -5.720446000 | 0.897835000 |
| 6 | -4.462831000 | 0.102852000 | -0.060171000 |
| 6 | -5.182580000 | 0.706534000 | -1.117300000 |
| 6 | -6.584800000 | 0.723235000 | -1.105354000 |
| 6 | -7.292963000 | 0.144788000 | -0.029251000 |
| 6 | -6.581227000 | -0.455171000 | 1.032533000 |
| 6 | -5.178528000 | -0.479132000 | 1.011733000 |
| 1 | -4.645689000 | 1.146908000 | -1.952695000 |
| 1 | -7.138221000 | 1.178356000 | -1.920969000 |
| 1 | -7.124393000 | -0.894730000 | 1.861600000 |
| 1 | -4.636756000 | -0.936245000 | 1.834959000 |
| 6 | 0.057663000 | 5.709680000 | -1.171958000 |

| | | | |
|---|---|---|---|
| 6 | -8.782167000 | 0.187863000 | -0.051901000 |
| 8 | -9.464606000 | 0.708977000 | -0.960397000 |
| 1 | 8.124597000 | 1.161518000 | 2.021978000 |
| 7 | 9.771884000 | -0.170642000 | 0.376806000 |
| 1 | 10.265081000 | 0.339615000 | 1.095686000 |
| 8 | -9.348481000 | -0.414746000 | 1.047261000 |
| 6 | -10.824758000 | -0.431612000 | 1.133516000 |
| 1 | -11.244011000 | -0.959224000 | 0.272149000 |
| 1 | -11.044565000 | -0.960573000 | 2.060899000 |
| 1 | -11.209192000 | 0.591829000 | 1.165639000 |
| 1 | 1.545438000 | -5.145229000 | -1.986004000 |
| 1 | 1.503361000 | -7.630067000 | -2.042109000 |
| 1 | -0.711816000 | -7.675621000 | 1.671747000 |
| 1 | -0.677273000 | -5.190820000 | 1.722670000 |
| 1 | -0.399692000 | 5.161999000 | -1.992105000 |
| 1 | 1.675638000 | 5.196773000 | 1.801159000 |
| 1 | -0.348320000 | 7.646628000 | -2.043450000 |
| 1 | 1.719727000 | 7.681507000 | 1.754424000 |
| 1 | 0.709894000 | 8.920196000 | -0.169217000 |
| 1 | 0.376363000 | -8.908988000 | -0.212138000 |
| 79 | 0.534380000 | 0.005435000 | -0.042188000 |
| 1 | 10.320414000 | -0.710638000 | -0.277185000 |

**2a**

| | | | |
|---|---|---|---|
| 7 | 2.061948000 | 1.481007000 | 0.052744000 |
| 7 | -0.943937000 | 1.530363000 | -0.055741000 |
| 7 | 2.015401000 | -1.519083000 | -0.056993000 |
| 7 | -0.992526000 | -1.471061000 | -0.038295000 |
| 6 | 3.427688000 | 1.225894000 | -0.003776000 |
| 6 | 4.110726000 | 2.509121000 | -0.135007000 |
| 6 | 3.151086000 | 3.497880000 | -0.141945000 |
| 6 | 1.848266000 | 2.851990000 | -0.018929000 |
| 1 | 5.177990000 | 2.636982000 | -0.236077000 |
| 1 | 3.311746000 | 4.560930000 | -0.243598000 |
| 6 | 0.589513000 | 3.517162000 | -0.006442000 |
| 6 | 4.057792000 | -0.051755000 | 0.033857000 |
| 6 | -1.973429000 | 3.583390000 | 0.088693000 |
| 6 | -2.963155000 | 2.626295000 | 0.069578000 |
| 6 | -2.315952000 | 1.319730000 | -0.030737000 |
| 6 | -0.687373000 | 2.895841000 | 0.001992000 |
| 1 | -2.103284000 | 4.652216000 | 0.172200000 |
| 1 | -4.028403000 | 2.792221000 | 0.133887000 |
| 6 | -2.978023000 | 0.062068000 | -0.072442000 |
| 6 | 1.755173000 | -2.882454000 | 0.004457000 |
| 6 | 3.031479000 | -3.570561000 | 0.168722000 |
| 6 | 4.022307000 | -2.613489000 | 0.197431000 |
| 6 | 3.386014000 | -1.308494000 | 0.046623000 |
| 1 | 3.154021000 | -4.638491000 | 0.272357000 |
| 1 | 5.080734000 | -2.776360000 | 0.334218000 |
| 6 | -3.041893000 | -2.501026000 | -0.220016000 |
| 6 | -2.083374000 | -3.489575000 | -0.215614000 |
| 6 | -0.778678000 | -2.844061000 | -0.092328000 |
| 6 | -2.355811000 | -1.216228000 | -0.099275000 |
| 1 | -4.109843000 | -2.632326000 | -0.313275000 |
| 1 | -2.245124000 | -4.553482000 | -0.305864000 |
| 6 | 0.476588000 | -3.506397000 | -0.048434000 |
| 6 | 0.616587000 | 5.021073000 | -0.010685000 |
| 6 | 1.159873000 | 5.734888000 | 1.083056000 |
| 6 | 1.182314000 | 7.142341000 | 1.083290000 |
| 6 | 0.670249000 | 7.859612000 | -0.016586000 |
| 6 | 0.131703000 | 7.157646000 | -1.113592000 |
| 6 | 5.555222000 | -0.075711000 | 0.065643000 |
| 6 | 6.303435000 | -0.729849000 | -0.943742000 |
| 6 | 7.704514000 | -0.752444000 | -0.922684000 |
| 6 | 8.421636000 | -0.127450000 | 0.134021000 |
| 6 | 7.678022000 | 0.525741000 | 1.154735000 |
| 6 | 6.277709000 | 0.553470000 | 1.109165000 |
| 1 | 5.780832000 | -1.208494000 | -1.768606000 |
| 1 | 8.250610000 | -1.250003000 | -1.721391000 |
| 1 | 5.733973000 | 1.051385000 | 1.908570000 |
| 6 | 0.454944000 | -5.010409000 | -0.047908000 |
| 6 | 1.004956000 | -5.739090000 | -1.128369000 |
| 6 | 0.981156000 | -7.146546000 | -1.132502000 |
| 6 | 0.415467000 | -7.849124000 | -0.049549000 |
| 6 | -0.129893000 | -7.132407000 | 1.034480000 |
| 6 | -0.114033000 | -5.724787000 | 1.032239000 |
| 6 | -4.480603000 | 0.085736000 | -0.088248000 |

| | | | |
|---|---|---|---|
| 6 | -5.184397000 | 0.650535000 | -1.178732000 |
| 6 | -6.586348000 | 0.669152000 | -1.195427000 |
| 6 | -7.317772000 | 0.129214000 | -0.114058000 |
| 6 | -6.625042000 | -0.432696000 | 0.981521000 |
| 6 | -5.222293000 | -0.456118000 | 0.988360000 |
| 1 | -4.630164000 | 1.060032000 | -2.018477000 |
| 1 | -7.122711000 | 1.095577000 | -2.037771000 |
| 1 | -7.183689000 | -0.842546000 | 1.815785000 |
| 1 | -4.696271000 | -0.881770000 | 1.838199000 |
| 6 | 0.101171000 | 5.750285000 | -1.107669000 |
| 6 | -8.803666000 | 0.171539000 | -0.167358000 |
| 8 | -9.472043000 | 0.661861000 | -1.104375000 |
| 1 | 8.202848000 | 1.004682000 | 1.978564000 |
| 7 | 9.809663000 | -0.154379000 | 0.168333000 |
| 1 | 10.320439000 | 0.289694000 | 0.917664000 |
| 8 | -9.394675000 | -0.395056000 | 0.940760000 |
| 6 | -10.871392000 | -0.406424000 | 0.995511000 |
| 1 | -11.274484000 | -0.963449000 | 0.144814000 |
| 1 | -11.112848000 | -0.901810000 | 1.936136000 |
| 1 | -11.255195000 | 0.617815000 | 0.983135000 |
| 1 | 1.437194000 | -5.203456000 | -1.969898000 |
| 1 | 1.400515000 | -7.689873000 | -1.975655000 |
| 1 | -0.563913000 | -7.664828000 | 1.877178000 |
| 1 | -0.529834000 | -5.178504000 | 1.875202000 |
| 1 | -0.308939000 | 5.215256000 | -1.960561000 |
| 1 | 1.550663000 | 5.188177000 | 1.937622000 |
| 1 | -0.261302000 | 7.701252000 | -1.969162000 |
| 1 | 1.595815000 | 7.674307000 | 1.936523000 |
| 1 | 0.690639000 | 8.946597000 | -0.018829000 |
| 1 | 0.400213000 | -8.936196000 | -0.050293000 |
| 79 | 0.534984000 | 0.005262000 | -0.024437000 |
| 1 | 10.339110000 | -0.624635000 | -0.551409000 |

**[3a]⁺**

| | | | |
|---|---|---|---|
| 7 | -1.424907000 | 1.507089000 | -0.092426000 |
| 7 | 1.474077000 | 1.472725000 | 0.029136000 |
| 7 | -1.460503000 | -1.391005000 | -0.072655000 |
| 7 | 1.439224000 | -1.426345000 | 0.016984000 |
| 6 | -2.810605000 | 1.308016000 | -0.059309000 |
| 6 | -3.452297000 | 2.596129000 | 0.081059000 |
| 6 | -2.469574000 | 3.555169000 | 0.110634000 |
| 6 | -1.193848000 | 2.885277000 | -0.007256000 |
| 1 | -4.517568000 | 2.742369000 | 0.166087000 |
| 1 | -2.592362000 | 4.621096000 | 0.221550000 |
| 6 | 0.049960000 | 3.539156000 | -0.030353000 |
| 6 | -3.493088000 | 0.082344000 | -0.150070000 |
| 6 | 2.570261000 | 3.495898000 | -0.158063000 |
| 6 | 3.529193000 | 2.513924000 | -0.120436000 |
| 6 | 2.853941000 | 1.240962000 | 0.007545000 |
| 6 | 1.276889000 | 2.856759000 | -0.052939000 |
| 1 | 2.719901000 | 4.559071000 | -0.262418000 |
| 1 | 4.598837000 | 2.636322000 | -0.189708000 |
| 6 | 3.501810000 | -0.001383000 | 0.096762000 |
| 6 | -1.256984000 | -2.774418000 | -0.143248000 |
| 6 | -2.537244000 | -3.413748000 | -0.349139000 |
| 6 | -3.496844000 | -2.431730000 | -0.383488000 |
| 6 | -2.835580000 | -1.159262000 | -0.197730000 |
| 1 | -2.677304000 | -4.476537000 | -0.469121000 |
| 1 | -4.557421000 | -2.552830000 | -0.538127000 |
| 6 | 3.452917000 | -2.516264000 | 0.312824000 |
| 6 | 2.470585000 | -3.475003000 | 0.279632000 |
| 6 | 1.203866000 | -2.805228000 | 0.081670000 |
| 6 | 2.819206000 | -1.227562000 | 0.138274000 |
| 1 | 4.511511000 | -2.663991000 | 0.459455000 |
| 1 | 2.586430000 | -4.541362000 | 0.393722000 |
| 6 | -0.034075000 | -3.458025000 | -0.031973000 |
| 6 | 0.066866000 | 5.039551000 | -0.028244000 |
| 6 | -0.455823000 | 5.763925000 | -1.124276000 |
| 6 | -0.434963000 | 7.170901000 | -1.124400000 |
| 6 | 0.097657000 | 7.871604000 | -0.023808000 |
| 6 | 0.614726000 | 7.156069000 | 1.074602000 |
| 6 | -4.989793000 | 0.099072000 | -0.193849000 |
| 6 | -5.752132000 | -0.502262000 | 0.831825000 |
| 6 | -7.155959000 | -0.488753000 | 0.810859000 |
| 6 | -7.834127000 | 0.128092000 | -0.267394000 |
| 6 | -7.078565000 | 0.727181000 | -1.304880000 |
| 6 | -5.678944000 | 0.718145000 | -1.262810000 |

| | | | |
|---|---|---|---|
| 1 | -5.245977000 | -0.972014000 | 1.671309000 |
| 1 | -7.723990000 | -0.943286000 | 1.610639000 |
| 1 | -5.120102000 | 1.177249000 | -2.073756000 |
| 6 | -0.052491000 | -4.958429000 | -0.035536000 |
| 6 | -0.669814000 | -5.670248000 | 1.018719000 |
| 6 | -0.681748000 | -7.077341000 | 1.018108000 |
| 6 | -0.087238000 | -7.790419000 | -0.042079000 |
| 6 | 0.524551000 | -7.087327000 | -1.099074000 |
| 6 | 0.547111000 | -5.680356000 | -1.093162000 |
| 6 | 5.001295000 | -0.019372000 | 0.149423000 |
| 6 | 5.687102000 | 0.524748000 | 1.259954000 |
| 6 | 7.088405000 | 0.505218000 | 1.309980000 |
| 6 | 7.829371000 | -0.049847000 | 0.243798000 |
| 6 | 7.151333000 | -0.590816000 | -0.870416000 |
| 6 | 5.748789000 | -0.579148000 | -0.912046000 |
| 1 | 5.124112000 | 0.947851000 | 2.087032000 |
| 1 | 7.615790000 | 0.914993000 | 2.165849000 |
| 1 | 7.720027000 | -1.012679000 | -1.691506000 |
| 1 | 5.233475000 | -0.990364000 | -1.775465000 |
| 6 | 0.604807000 | 5.748966000 | 1.070183000 |
| 6 | 9.316917000 | -0.044250000 | 0.332739000 |
| 8 | 9.969726000 | 0.425286000 | 1.289793000 |
| 1 | -7.585870000 | 1.198556000 | -2.143861000 |
| 6 | -10.206802000 | -0.321613000 | 0.487361000 |
| 8 | -9.922778000 | -0.944246000 | 1.549344000 |
| 7 | -9.248927000 | 0.185899000 | -0.374100000 |
| 1 | -9.601157000 | 0.665506000 | -1.196227000 |
| 8 | 9.917452000 | -0.617668000 | -0.763583000 |
| 6 | 11.395473000 | -0.665494000 | -0.787178000 |
| 1 | 11.765150000 | -1.237317000 | 0.068640000 |
| 1 | 11.644180000 | -1.161225000 | -1.725533000 |
| 1 | 11.802820000 | 0.349171000 | -0.760083000 |
| 6 | -11.648073000 | -0.086630000 | 0.065176000 |
| 1 | -12.189871000 | 0.385884000 | 0.891435000 |
| 1 | -11.744991000 | 0.540273000 | -0.826398000 |
| 1 | -12.126244000 | -1.053419000 | -0.131146000 |
| 1 | -1.124504000 | -5.126445000 | 1.842925000 |
| 1 | -1.151187000 | -7.612059000 | 1.839530000 |
| 1 | 0.980726000 | -7.629661000 | -1.922994000 |
| 1 | 1.014791000 | -5.144012000 | -1.915021000 |
| 1 | 0.998570000 | 5.203189000 | 1.923924000 |
| 1 | -0.862023000 | 5.229387000 | -1.979334000 |
| 1 | 1.022574000 | 7.688830000 | 1.929498000 |
| 1 | -0.831203000 | 7.715162000 | -1.977528000 |
| 1 | 0.109448000 | 8.958396000 | -0.022095000 |
| 1 | -0.100498000 | -8.877192000 | -0.044538000 |
| 79 | 0.007056000 | 0.040538000 | -0.029716000 |

**3a**

| | | | |
|---|---|---|---|
| 7 | -1.479178000 | 1.554161000 | -0.109391000 |
| 7 | 1.521527000 | 1.527879000 | 0.049053000 |
| 7 | -1.508091000 | -1.448746000 | -0.040464000 |
| 7 | 1.496512000 | -1.475619000 | 0.001838000 |
| 6 | -2.850786000 | 1.332273000 | -0.094774000 |
| 6 | -3.508125000 | 2.633045000 | 0.001711000 |
| 6 | -2.525865000 | 3.598060000 | 0.032685000 |
| 6 | -1.234584000 | 2.920033000 | -0.042908000 |
| 1 | -4.575058000 | 2.788392000 | 0.061777000 |
| 1 | -2.664138000 | 4.665598000 | 0.118888000 |
| 6 | 0.039270000 | 3.553122000 | -0.025441000 |
| 6 | -3.505933000 | 0.070223000 | -0.141150000 |
| 6 | 2.604887000 | 3.554581000 | -0.067511000 |
| 6 | 3.569838000 | 2.572861000 | -0.033885000 |
| 6 | 2.888281000 | 1.282729000 | 0.047370000 |
| 6 | 1.300757000 | 2.898903000 | -0.008283000 |
| 1 | 2.762836000 | 4.620154000 | -0.143381000 |
| 1 | 4.639863000 | 2.712283000 | -0.078392000 |
| 6 | 3.517464000 | 0.008336000 | 0.091236000 |
| 6 | -1.283794000 | -2.818850000 | -0.087962000 |
| 6 | -2.579232000 | -3.474701000 | -0.244818000 |
| 6 | -3.544426000 | -2.492808000 | -0.279406000 |
| 6 | -2.871927000 | -1.203253000 | -0.143495000 |
| 1 | -2.730434000 | -4.540090000 | -0.335472000 |
| 1 | -4.608097000 | -2.630360000 | -0.404815000 |
| 6 | 3.516072000 | -2.556665000 | 0.219323000 |
| 6 | 2.533684000 | -3.521026000 | 0.189643000 |
| 6 | 1.248159000 | -2.842367000 | 0.044727000 |

| | | | |
|---|---|---|---|
| 6 | 2.864295000 | -1.254649000 | 0.093494000 |
| 1 | 4.578161000 | -2.714948000 | 0.334590000 |
| 1 | 2.667073000 | -4.589143000 | 0.276163000 |
| 6 | -0.023004000 | -3.474166000 | -0.020932000 |
| 6 | 0.051343000 | 5.057292000 | -0.021227000 |
| 6 | -0.442895000 | 5.785409000 | -1.128574000 |
| 6 | -0.427547000 | 7.192989000 | -1.127980000 |
| 6 | 0.073738000 | 7.895883000 | -0.013996000 |
| 6 | 0.563576000 | 7.179514000 | 1.096449000 |
| 6 | -5.006878000 | 0.084196000 | -0.191497000 |
| 6 | -5.778590000 | -0.472611000 | 0.852244000 |
| 6 | -7.183533000 | -0.461795000 | 0.822896000 |
| 6 | -7.854870000 | 0.110257000 | -0.282917000 |
| 6 | -7.093308000 | 0.666599000 | -1.338670000 |
| 6 | -5.693114000 | 0.657550000 | -1.287789000 |
| 1 | -5.276197000 | -0.907549000 | 1.712501000 |
| 1 | -7.755981000 | -0.884459000 | 1.637167000 |
| 1 | -5.128263000 | 1.083473000 | -2.112585000 |
| 6 | -0.037324000 | -4.978336000 | -0.022543000 |
| 6 | -0.610153000 | -5.694745000 | 1.054125000 |
| 6 | -0.620275000 | -7.102398000 | 1.056090000 |
| 6 | -0.065643000 | -7.816689000 | -0.024857000 |
| 6 | 0.503098000 | -7.111874000 | -1.104665000 |
| 6 | 0.521030000 | -5.704269000 | -1.100405000 |
| 6 | 5.019558000 | -0.005425000 | 0.139447000 |
| 6 | 5.712549000 | 0.534024000 | 1.249416000 |
| 6 | 7.113786000 | 0.516227000 | 1.297449000 |
| 6 | 7.855062000 | -0.035085000 | 0.228691000 |
| 6 | 7.173098000 | -0.571267000 | -0.886292000 |
| 6 | 5.770701000 | -0.558605000 | -0.924611000 |
| 1 | 5.150359000 | 0.951949000 | 2.079645000 |
| 1 | 7.641864000 | 0.922955000 | 2.154616000 |
| 1 | 7.739763000 | -0.989873000 | -1.710730000 |
| 1 | 5.253277000 | -0.964916000 | -1.789047000 |
| 6 | 0.556495000 | 5.771872000 | 1.089990000 |
| 6 | 9.340125000 | -0.032244000 | 0.315772000 |
| 8 | 9.999346000 | 0.432670000 | 1.271960000 |
| 1 | -7.596100000 | 1.104438000 | -2.198789000 |
| 6 | -10.236815000 | -0.314063000 | 0.462836000 |
| 8 | -9.966922000 | -0.900761000 | 1.549993000 |
| 7 | -9.271671000 | 0.164480000 | -0.402960000 |
| 1 | -9.614613000 | 0.616494000 | -1.244302000 |
| 8 | 9.940943000 | -0.605155000 | -0.783565000 |
| 6 | 11.417772000 | -0.655752000 | -0.805051000 |
| 1 | 11.786420000 | -1.229234000 | 0.050305000 |
| 1 | 11.667268000 | -1.150835000 | -1.743718000 |
| 1 | 11.828194000 | 0.357755000 | -0.776222000 |
| 6 | -11.674397000 | -0.094303000 | 0.017420000 |
| 1 | -12.218854000 | 0.428222000 | 0.811465000 |
| 1 | -11.761076000 | 0.481865000 | -0.908913000 |
| 1 | -12.157473000 | -1.067655000 | -0.127138000 |
| 1 | -1.033881000 | -5.150238000 | 1.894292000 |
| 1 | -1.057323000 | -7.636647000 | 1.896008000 |
| 1 | 0.929261000 | -7.653432000 | -1.945519000 |
| 1 | 0.955128000 | -5.166905000 | -1.939909000 |
| 1 | 0.929249000 | 5.225862000 | 1.953014000 |
| 1 | -0.824754000 | 5.249653000 | -1.994070000 |
| 1 | 0.947936000 | 7.712138000 | 1.962746000 |
| 1 | -0.803447000 | 7.736096000 | -1.991499000 |
| 1 | 0.082405000 | 8.983002000 | -0.011244000 |
| 1 | -0.076498000 | -8.903791000 | -0.025738000 |
| 79 | 0.007757000 | 0.039271000 | -0.024855000 |

**[4a]$^+$**

| | | | |
|---|---|---|---|
| 7 | -1.200372000 | 1.512789000 | -0.099938000 |
| 7 | 1.698137000 | 1.440906000 | -0.002995000 |
| 7 | -1.273383000 | -1.385044000 | -0.086832000 |
| 7 | 1.625571000 | -1.458291000 | -0.019646000 |
| 6 | -2.588117000 | 1.331501000 | -0.050257000 |
| 6 | -3.211571000 | 2.627889000 | 0.095930000 |
| 6 | -2.216377000 | 3.574378000 | 0.111134000 |
| 6 | -0.950851000 | 2.887910000 | -0.020581000 |
| 1 | -4.273777000 | 2.787818000 | 0.193688000 |
| 1 | -2.324025000 | 4.642023000 | 0.221334000 |
| 6 | 0.301012000 | 3.525672000 | -0.058501000 |
| 6 | -3.287362000 | 0.114809000 | -0.134130000 |
| 6 | 2.819835000 | 3.449093000 | -0.200608000 |

| | | | |
|---|---|---|---|
| 6 | 3.766010000 | 2.454859000 | -0.162930000 |
| 6 | 3.074553000 | 1.191112000 | -0.029256000 |
| 6 | 1.518653000 | 2.827262000 | -0.087840000 |
| 1 | 2.982866000 | 4.509891000 | -0.308986000 |
| 1 | 4.836879000 | 2.563374000 | -0.236575000 |
| 6 | 3.706067000 | -0.059745000 | 0.058176000 |
| 6 | -1.088519000 | -2.770556000 | -0.167557000 |
| 6 | -2.379066000 | -3.392147000 | -0.363221000 |
| 6 | -3.326279000 | -2.397719000 | -0.380388000 |
| 6 | -2.646567000 | -1.134988000 | -0.194818000 |
| 1 | -2.534182000 | -4.452286000 | -0.488033000 |
| 1 | -4.390018000 | -2.504193000 | -0.523617000 |
| 6 | 3.626384000 | -2.574953000 | 0.264169000 |
| 6 | 2.632009000 | -3.521051000 | 0.228604000 |
| 6 | 1.373137000 | -2.834317000 | 0.038877000 |
| 6 | 3.008399000 | -1.277454000 | 0.097954000 |
| 1 | 4.683392000 | -2.737220000 | 0.406810000 |
| 1 | 2.734810000 | -4.589394000 | 0.336495000 |
| 6 | 0.126375000 | -3.470611000 | -0.071023000 |
| 6 | 0.336479000 | 5.025681000 | -0.064534000 |
| 6 | -0.186953000 | 5.749965000 | -1.160311000 |
| 6 | -0.149490000 | 7.156573000 | -1.168734000 |
| 6 | 0.400607000 | 7.857110000 | -0.076674000 |
| 6 | 0.918444000 | 7.141736000 | 1.021479000 |
| 6 | -4.784210000 | 0.149584000 | -0.157379000 |
| 6 | -5.538725000 | -0.446026000 | 0.877440000 |
| 6 | -6.942455000 | -0.417800000 | 0.875806000 |
| 6 | -7.628720000 | 0.208200000 | -0.191976000 |
| 6 | -6.881217000 | 0.801825000 | -1.238399000 |
| 6 | -5.481297000 | 0.778300000 | -1.215491000 |
| 1 | -5.025977000 | -0.923191000 | 1.708713000 |
| 1 | -7.504320000 | -0.868345000 | 1.682169000 |
| 1 | -4.928911000 | 1.233072000 | -2.033278000 |
| 6 | 0.089310000 | -4.970548000 | -0.084715000 |
| 6 | -0.529479000 | -5.682365000 | 0.968675000 |
| 6 | -0.558079000 | -7.089174000 | 0.958374000 |
| 6 | 0.021052000 | -7.801794000 | -0.110602000 |
| 6 | 0.634225000 | -7.098615000 | -1.166728000 |
| 6 | 0.673490000 | -5.692092000 | -1.151223000 |
| 6 | 5.205307000 | -0.096222000 | 0.110264000 |
| 6 | 5.896504000 | 0.440144000 | 1.221673000 |
| 6 | 7.296962000 | 0.403401000 | 1.273554000 |
| 6 | 8.030542000 | -0.162289000 | 0.207405000 |
| 6 | 7.348064000 | -0.695178000 | -0.907962000 |
| 6 | 5.946016000 | -0.665455000 | -0.950910000 |
| 1 | 5.337667000 | 0.870402000 | 2.047741000 |
| 1 | 7.828343000 | 0.806895000 | 2.129919000 |
| 1 | 7.912391000 | -1.124550000 | -1.728219000 |
| 1 | 5.426115000 | -1.069947000 | -1.814605000 |
| 6 | 0.891910000 | 5.734840000 | 1.025290000 |
| 6 | 9.513330000 | -0.177653000 | 0.295920000 |
| 8 | 10.182839000 | 0.281095000 | 1.243989000 |
| 1 | -7.395162000 | 1.279895000 | -2.069511000 |
| 6 | -9.995439000 | -0.222088000 | 0.591998000 |
| 8 | -9.703267000 | -0.851396000 | 1.647824000 |
| 7 | -9.044125000 | 0.280377000 | -0.279652000 |
| 1 | -9.402122000 | 0.766331000 | -1.095533000 |
| 8 | 10.115994000 | -0.765240000 | -0.801104000 |
| 1 | 11.096965000 | -0.762894000 | -0.719076000 |
| 6 | -11.439940000 | 0.027009000 | 0.189422000 |
| 1 | -11.966752000 | 0.501453000 | 1.024224000 |
| 1 | -11.543092000 | 0.657586000 | -0.698848000 |
| 1 | -11.929036000 | -0.934955000 | -0.003679000 |
| 1 | -0.972132000 | -5.139082000 | 1.799744000 |
| 1 | -1.028375000 | -7.624047000 | 1.779202000 |
| 1 | 1.078682000 | -7.640500000 | -1.997315000 |
| 1 | 1.142247000 | -5.155557000 | -1.972335000 |
| 1 | 1.286145000 | 5.189353000 | 1.878985000 |
| 1 | -0.606832000 | 5.215375000 | -2.008719000 |
| 1 | 1.339576000 | 7.674495000 | 1.869910000 |
| 1 | -0.546544000 | 7.700643000 | -2.021605000 |
| 1 | 0.425086000 | 8.943675000 | -0.081268000 |
| 1 | -0.004956000 | -8.888286000 | -0.120435000 |
| 79 | 0.212607000 | 0.027510000 | -0.052303000 |

**4a**

| | | | |
|---|---|---|---|
| 7 | -1.256263000 | 1.556853000 | -0.114756000 |

| | | | |
|---|---|---|---|
| 7 | 1.744988000 | 1.496071000 | 0.020763000 |
| 7 | -1.319084000 | -1.445571000 | -0.048791000 |
| 7 | 1.685149000 | -1.507106000 | -0.030787000 |
| 6 | -2.630178000 | 1.350754000 | -0.089034000 |
| 6 | -3.271764000 | 2.658896000 | 0.013481000 |
| 6 | -2.278264000 | 3.612535000 | 0.036960000 |
| 6 | -0.995549000 | 2.919736000 | -0.049312000 |
| 1 | -4.336323000 | 2.826358000 | 0.082274000 |
| 1 | -2.403506000 | 4.681542000 | 0.124782000 |
| 6 | 0.285590000 | 3.538221000 | -0.041151000 |
| 6 | -3.300121000 | 0.096339000 | -0.130781000 |
| 6 | 2.850582000 | 3.510403000 | -0.101913000 |
| 6 | 3.804445000 | 2.517740000 | -0.075793000 |
| 6 | 3.108671000 | 1.235366000 | 0.009182000 |
| 6 | 1.539508000 | 2.869614000 | -0.033918000 |
| 1 | 3.020056000 | 4.574169000 | -0.178154000 |
| 1 | 4.875606000 | 2.645350000 | -0.128025000 |
| 6 | 3.723128000 | -0.046394000 | 0.046724000 |
| 6 | -1.111010000 | -2.818043000 | -0.100273000 |
| 6 | -2.415189000 | -3.458767000 | -0.247126000 |
| 6 | -3.369258000 | -2.465807000 | -0.271991000 |
| 6 | -2.680804000 | -1.184311000 | -0.140018000 |
| 1 | -2.579321000 | -4.522199000 | -0.338166000 |
| 1 | -4.435488000 | -2.590777000 | -0.388518000 |
| 6 | 3.693677000 | -2.611667000 | 0.170516000 |
| 6 | 2.700090000 | -3.564601000 | 0.146440000 |
| 6 | 1.421448000 | -2.871026000 | 0.012048000 |
| 6 | 3.055932000 | -1.302041000 | 0.051228000 |
| 1 | 4.754600000 | -2.782672000 | 0.278097000 |
| 1 | 2.821875000 | -4.634305000 | 0.230373000 |
| 6 | 0.142658000 | -3.488027000 | -0.044537000 |
| 6 | 0.314991000 | 5.042146000 | -0.035966000 |
| 6 | -0.177955000 | 5.776767000 | -1.139571000 |
| 6 | -0.145997000 | 7.184061000 | -1.138135000 |
| 6 | 0.370835000 | 7.880215000 | -0.027020000 |
| 6 | 0.859455000 | 7.157324000 | 1.079734000 |
| 6 | -4.801181000 | 0.127560000 | -0.167269000 |
| 6 | -5.569297000 | -0.421023000 | 0.883470000 |
| 6 | -6.974217000 | -0.393496000 | 0.867732000 |
| 6 | -7.649318000 | 0.187352000 | -0.231187000 |
| 6 | -6.891463000 | 0.735267000 | -1.293991000 |
| 6 | -5.491049000 | 0.709639000 | -1.256649000 |
| 1 | -5.063780000 | -0.862556000 | 1.738524000 |
| 1 | -7.543714000 | -0.810040000 | 1.687205000 |
| 1 | -4.929153000 | 1.129355000 | -2.086629000 |
| 6 | 0.110943000 | -4.991923000 | -0.048382000 |
| 6 | -0.462251000 | -5.703368000 | 1.031387000 |
| 6 | -0.488882000 | -7.110783000 | 1.031148000 |
| 6 | 0.049289000 | -7.829719000 | -0.055038000 |
| 6 | 0.618245000 | -7.129771000 | -1.137898000 |
| 6 | 0.652764000 | -5.722494000 | -1.131528000 |
| 6 | 5.225114000 | -0.077291000 | 0.085947000 |
| 6 | 5.929708000 | 0.454467000 | 1.192836000 |
| 6 | 7.330332000 | 0.420600000 | 1.234192000 |
| 6 | 8.058415000 | -0.139584000 | 0.160402000 |
| 6 | 7.365696000 | -0.668038000 | -0.951718000 |
| 6 | 5.963630000 | -0.639239000 | -0.982473000 |
| 1 | 5.376144000 | 0.878482000 | 2.025624000 |
| 1 | 7.867255000 | 0.820911000 | 2.088867000 |
| 1 | 7.923414000 | -1.092999000 | -1.779049000 |
| 1 | 5.436718000 | -1.039255000 | -1.843956000 |
| 6 | 0.835754000 | 5.749877000 | 1.072429000 |
| 6 | 9.538953000 | -0.155084000 | 0.237481000 |
| 8 | 10.220061000 | 0.292860000 | 1.184099000 |
| 1 | -7.397435000 | 1.179450000 | -2.148984000 |
| 6 | -10.028896000 | -0.209689000 | 0.536725000 |
| 8 | -9.755469000 | -0.801994000 | 1.619928000 |
| 7 | -9.066410000 | 0.258912000 | -0.337487000 |
| 1 | -9.411631000 | 0.716566000 | -1.174841000 |
| 8 | 10.137351000 | -0.730946000 | -0.870845000 |
| 1 | 11.118377000 | -0.726638000 | -0.790627000 |
| 6 | -11.468043000 | 0.028381000 | 0.106025000 |
| 1 | -11.998415000 | 0.555025000 | 0.906844000 |
| 1 | -11.557021000 | 0.608146000 | -0.817814000 |
| 1 | -11.964027000 | -0.938770000 | -0.036438000 |
| 1 | -0.873301000 | -5.155378000 | 1.875569000 |
| 1 | -0.925934000 | -7.641293000 | 1.873435000 |
| 1 | 1.031774000 | -7.674866000 | -1.982765000 |

| | | | |
|---|---|---|---|
| 1 | 1.086827000 | -5.188829000 | -1.973397000 |
| 1 | 1.207648000 | 5.198884000 | 1.932650000 |
| 1 | -0.571759000 | 5.246147000 | -2.002871000 |
| 1 | 1.255699000 | 7.684776000 | 1.943842000 |
| 1 | -0.521130000 | 7.732204000 | -1.998806000 |
| 1 | 0.392307000 | 8.967154000 | -0.023647000 |
| 1 | 0.025680000 | -8.916617000 | -0.057606000 |
| 79 | 0.213782000 | 0.024972000 | -0.043371000 |

**[4b]$^+$**

| | | | |
|---|---|---|---|
| 7 | 1.466667000 | 1.376593000 | -0.209349000 |
| 7 | 1.529402000 | -1.520486000 | -0.132885000 |
| 7 | -1.429344000 | 1.320164000 | -0.002343000 |
| 7 | -1.370787000 | -1.577899000 | 0.043103000 |
| 6 | 1.224749000 | 2.754260000 | -0.141691000 |
| 6 | 2.496174000 | 3.435013000 | -0.045971000 |
| 6 | 3.486645000 | 2.483335000 | -0.077587000 |
| 6 | 2.854125000 | 1.189127000 | -0.196252000 |
| 1 | 2.612369000 | 4.502605000 | 0.055442000 |
| 1 | 4.551641000 | 2.638938000 | -0.007246000 |
| 6 | 3.546565000 | -0.032139000 | -0.295165000 |
| 6 | -0.025677000 | 3.397099000 | -0.167473000 |
| 6 | 3.560011000 | -2.548535000 | -0.514875000 |
| 6 | 2.610978000 | -3.537775000 | -0.431920000 |
| 6 | 1.334590000 | -2.905675000 | -0.181642000 |
| 6 | 2.897181000 | -1.278897000 | -0.313086000 |
| 1 | 4.613911000 | -2.662614000 | -0.713736000 |
| 1 | 2.756746000 | -4.600276000 | -0.549313000 |
| 6 | 0.121858000 | -3.592902000 | -0.011469000 |
| 6 | -2.807370000 | 1.073974000 | 0.011634000 |
| 6 | -3.496128000 | 2.332206000 | -0.166175000 |
| 6 | -2.547893000 | 3.321466000 | -0.264759000 |
| 6 | -1.247423000 | 2.701351000 | -0.148225000 |
| 1 | -4.567650000 | 2.438921000 | -0.228227000 |
| 1 | -2.711348000 | 4.376221000 | -0.420460000 |
| 6 | -2.364775000 | -3.626303000 | 0.424077000 |
| 6 | -3.352930000 | -2.674492000 | 0.485328000 |
| 6 | -2.745519000 | -1.386526000 | 0.231847000 |
| 6 | -1.118004000 | -2.951167000 | 0.139148000 |
| 1 | -2.464656000 | -4.689186000 | 0.579892000 |
| 1 | -4.399072000 | -2.824501000 | 0.700489000 |
| 6 | -3.446398000 | -0.169298000 | 0.175643000 |
| 6 | 5.039866000 | -0.002094000 | -0.388086000 |
| 6 | 5.685931000 | 0.640064000 | -1.474312000 |
| 6 | 7.081694000 | 0.660565000 | -1.572714000 |
| 6 | 7.871254000 | 0.047493000 | -0.573592000 |
| 6 | 7.245807000 | -0.590009000 | 0.520383000 |
| 6 | -0.056420000 | 4.893135000 | -0.215539000 |
| 6 | -0.638547000 | 5.643584000 | 0.829868000 |
| 6 | -0.667130000 | 7.047079000 | 0.802018000 |
| 6 | -0.114105000 | 7.736935000 | -0.303153000 |
| 6 | 0.465155000 | 6.993304000 | -1.360348000 |
| 6 | 0.499204000 | 5.594442000 | -1.311439000 |
| 1 | -1.058726000 | 5.128872000 | 1.690111000 |
| 1 | -1.105647000 | 7.606160000 | 1.616902000 |
| 1 | 0.941733000 | 5.044561000 | -2.137571000 |
| 6 | -4.937774000 | -0.193991000 | 0.295923000 |
| 6 | -5.587098000 | 0.469579000 | 1.367251000 |
| 6 | -6.980501000 | 0.443409000 | 1.492663000 |
| 6 | -7.765013000 | -0.238885000 | 0.535334000 |
| 6 | -7.136562000 | -0.899866000 | -0.542935000 |
| 6 | -5.734683000 | -0.877008000 | -0.647861000 |
| 6 | 0.153422000 | -5.092707000 | 0.016018000 |
| 6 | 0.818265000 | -5.776230000 | 1.060911000 |
| 6 | 0.844819000 | -7.177471000 | 1.090890000 |
| 6 | 0.215281000 | -7.919635000 | 0.067274000 |
| 6 | -0.446132000 | -7.245004000 | -0.982154000 |
| 6 | -0.480138000 | -5.842526000 | -1.001615000 |
| 1 | 1.298747000 | -5.210983000 | 1.854261000 |
| 1 | 1.346520000 | -7.703034000 | 1.897514000 |
| 1 | -0.924662000 | -7.815769000 | -1.770130000 |
| 1 | -0.984218000 | -5.328397000 | -1.814950000 |
| 6 | 5.842277000 | -0.614573000 | 0.598542000 |
| 6 | 0.271012000 | -9.402665000 | 0.129034000 |
| 8 | 0.831809000 | -10.065662000 | 1.025476000 |
| 1 | 0.887683000 | 7.509350000 | -2.219737000 |
| 6 | -0.600772000 | 10.100047000 | 0.458932000 |

| | | | |
|---|---|---|---|
| 8 | -1.167507000 | 9.804643000 | 1.548698000 |
| 7 | -0.102347000 | 9.152123000 | -0.418746000 |
| 1 | 0.331186000 | 9.513546000 | -1.262257000 |
| 8 | -0.374278000 | -10.014324000 | -0.930047000 |
| 1 | -0.321398000 | -10.995315000 | -0.867131000 |
| 6 | -0.430692000 | 11.544521000 | 0.016845000 |
| 1 | 0.031499000 | 12.114919000 | 0.829311000 |
| 1 | 0.178352000 | 11.655230000 | -0.885477000 |
| 1 | -1.418892000 | 11.980930000 | -0.170864000 |
| 1 | -4.996030000 | 0.990923000 | 2.115606000 |
| 1 | -7.476366000 | 0.941309000 | 2.320309000 |
| 1 | -7.715064000 | -1.424295000 | -1.295355000 |
| 1 | -5.262343000 | -1.380777000 | -1.487281000 |
| 1 | 5.371965000 | -1.100117000 | 1.449723000 |
| 1 | 5.091576000 | 1.106919000 | -2.255337000 |
| 1 | 7.827792000 | -1.061121000 | 1.304688000 |
| 1 | 7.575554000 | 1.140982000 | -2.411823000 |
| 79 | 0.048934000 | -0.100636000 | -0.075867000 |
| 8 | -9.139373000 | -0.200902000 | 0.741139000 |
| 6 | -10.031598000 | -0.884739000 | -0.215513000 |
| 1 | -9.784169000 | -1.955979000 | -0.236107000 |
| 1 | -9.875277000 | -0.461952000 | -1.218424000 |
| 6 | -11.465529000 | -0.658624000 | 0.259854000 |
| 1 | -11.660513000 | 0.421524000 | 0.311371000 |
| 1 | -11.574182000 | -1.057701000 | 1.277921000 |
| 6 | -12.494553000 | -1.330554000 | -0.677304000 |
| 1 | -12.291053000 | -2.410809000 | -0.730992000 |
| 1 | -12.373255000 | -0.936231000 | -1.697724000 |
| 6 | -13.946937000 | -1.104163000 | -0.209024000 |
| 1 | -14.662086000 | -1.587277000 | -0.887301000 |
| 1 | -14.104271000 | -1.516421000 | 0.796983000 |
| 1 | -14.185607000 | -0.032475000 | -0.173831000 |
| 8 | 9.247162000 | 0.123371000 | -0.757543000 |
| 6 | 10.142966000 | -0.490066000 | 0.243066000 |
| 1 | 9.953696000 | -0.025826000 | 1.219681000 |
| 1 | 9.926686000 | -1.566408000 | 0.308754000 |
| 6 | 11.575549000 | -0.248708000 | -0.232784000 |
| 1 | 11.672652000 | -0.647258000 | -1.251919000 |
| 1 | 11.758811000 | 0.833467000 | -0.287540000 |
| 6 | 12.631946000 | -0.912024000 | 0.683860000 |
| 1 | 12.438645000 | -1.994066000 | 0.742233000 |
| 1 | 13.615414000 | -0.798180000 | 0.207026000 |
| 6 | 12.695523000 | -0.319596000 | 2.109965000 |
| 1 | 12.861094000 | 0.765965000 | 2.074065000 |
| 1 | 11.769757000 | -0.500213000 | 2.671705000 |
| 1 | 13.519786000 | -0.767235000 | 2.680097000 |

**4b**

| | | | |
|---|---|---|---|
| 7 | 1.491965000 | 1.421142000 | -0.213940000 |
| 7 | 1.558279000 | -1.577935000 | -0.081107000 |
| 7 | -1.503714000 | 1.365061000 | 0.042649000 |
| 7 | -1.446175000 | -1.637627000 | 0.053509000 |
| 6 | 1.232699000 | 2.785739000 | -0.166522000 |
| 6 | 2.517372000 | 3.478758000 | -0.128058000 |
| 6 | 3.509861000 | 2.524172000 | -0.162464000 |
| 6 | 2.865868000 | 1.215167000 | -0.224405000 |
| 1 | 2.646064000 | 4.548635000 | -0.058229000 |
| 1 | 4.575933000 | 2.691986000 | -0.126727000 |
| 6 | 3.536455000 | -0.040251000 | -0.265884000 |
| 6 | -0.047995000 | 3.404960000 | -0.135989000 |
| 6 | 3.598742000 | -2.604472000 | -0.356646000 |
| 6 | 2.647251000 | -3.596090000 | -0.268804000 |
| 6 | 1.349238000 | -2.950722000 | -0.086370000 |
| 6 | 2.916187000 | -1.319364000 | -0.228806000 |
| 1 | 4.659310000 | -2.732702000 | -0.513660000 |
| 1 | 2.810720000 | -4.661139000 | -0.341895000 |
| 6 | 0.098211000 | -3.615008000 | 0.038899000 |
| 6 | -2.867830000 | 1.103094000 | 0.068794000 |
| 6 | -3.565918000 | 2.379660000 | -0.054157000 |
| 6 | -2.614255000 | 3.371813000 | -0.139368000 |
| 6 | -1.301716000 | 2.735418000 | -0.072066000 |
| 1 | -4.638144000 | 2.501425000 | -0.092125000 |
| 1 | -2.787602000 | 4.431019000 | -0.257958000 |
| 6 | -2.449028000 | -3.686577000 | 0.354160000 |
| 6 | -3.440764000 | -2.732060000 | 0.395833000 |
| 6 | -2.812946000 | -1.428158000 | 0.197220000 |
| 6 | -1.179774000 | -2.998267000 | 0.130885000 |

| | | | |
|---|---|---|---|
| 1 | -2.567352000 | -4.752078000 | 0.484913000 |
| 1 | -4.494241000 | -2.895550000 | 0.566905000 |
| 6 | -3.485576000 | -0.175369000 | 0.175398000 |
| 6 | 5.035573000 | -0.009088000 | -0.348550000 |
| 6 | 5.692003000 | 0.566352000 | -1.465185000 |
| 6 | 7.090017000 | 0.595642000 | -1.549220000 |
| 6 | 7.872548000 | 0.055364000 | -0.505044000 |
| 6 | 7.239993000 | -0.518636000 | 0.618351000 |
| 6 | -0.078768000 | 4.905914000 | -0.173194000 |
| 6 | -0.608957000 | 5.652822000 | 0.902157000 |
| 6 | -0.639504000 | 7.057588000 | 0.885919000 |
| 6 | -0.138180000 | 7.754443000 | -0.238305000 |
| 6 | 0.390392000 | 7.018005000 | -1.325665000 |
| 6 | 0.423822000 | 5.617777000 | -1.287794000 |
| 1 | -0.989448000 | 5.130977000 | 1.776481000 |
| 1 | -1.040403000 | 7.610937000 | 1.723999000 |
| 1 | 0.827177000 | 5.072432000 | -2.136666000 |
| 6 | -4.983991000 | -0.201808000 | 0.270918000 |
| 6 | -5.653908000 | 0.401713000 | 1.364616000 |
| 6 | -7.050939000 | 0.376169000 | 1.463190000 |
| 6 | -7.819517000 | -0.248812000 | 0.456577000 |
| 6 | -7.173681000 | -0.852235000 | -0.643629000 |
| 6 | -5.768666000 | -0.826790000 | -0.721820000 |
| 6 | 0.130186000 | -5.116709000 | 0.081804000 |
| 6 | 0.771715000 | -5.794839000 | 1.146413000 |
| 6 | 0.798586000 | -7.195448000 | 1.191680000 |
| 6 | 0.190537000 | -7.950203000 | 0.163250000 |
| 6 | -0.448067000 | -7.284036000 | -0.906549000 |
| 6 | -0.480059000 | -5.882096000 | -0.940285000 |
| 1 | 1.234176000 | -5.220957000 | 1.944288000 |
| 1 | 1.283062000 | -7.712091000 | 2.014723000 |
| 1 | -0.909960000 | -7.861939000 | -1.699540000 |
| 1 | -0.965367000 | -5.375382000 | -1.769479000 |
| 6 | 5.834438000 | -0.549000000 | 0.682082000 |
| 6 | 0.243103000 | -9.429707000 | 0.241691000 |
| 8 | 0.784988000 | -10.088460000 | 1.154481000 |
| 1 | 0.773532000 | 7.540309000 | -2.200097000 |
| 6 | -0.595036000 | 10.116425000 | 0.550286000 |
| 8 | -1.121939000 | 9.819928000 | 1.660876000 |
| 7 | -0.129713000 | 9.173229000 | -0.346500000 |
| 1 | 0.272884000 | 9.536595000 | -1.204289000 |
| 8 | -0.383147000 | -10.055669000 | -0.823392000 |
| 1 | -0.330112000 | -11.035340000 | -0.743820000 |
| 6 | -0.440516000 | 11.563465000 | 0.107885000 |
| 1 | 0.074169000 | 12.124960000 | 0.894903000 |
| 1 | 0.114805000 | 11.675945000 | -0.828342000 |
| 1 | -1.434787000 | 12.008175000 | -0.017414000 |
| 1 | -5.074482000 | 0.879813000 | 2.150118000 |
| 1 | -7.560149000 | 0.831031000 | 2.307703000 |
| 1 | -7.739630000 | -1.334171000 | -1.433505000 |
| 1 | -5.281538000 | -1.286317000 | -1.578008000 |
| 1 | 5.357205000 | -0.984342000 | 1.556255000 |
| 1 | 5.102072000 | 0.978763000 | -2.279588000 |
| 1 | 7.816590000 | -0.935086000 | 1.437232000 |
| 1 | 7.589208000 | 1.028676000 | -2.411057000 |
| 79 | 0.024866000 | -0.107625000 | -0.050754000 |
| 8 | -9.201937000 | -0.216162000 | 0.639071000 |
| 6 | -10.072244000 | -0.843848000 | -0.371368000 |
| 1 | -9.828452000 | -1.913869000 | -0.444556000 |
| 1 | -9.893157000 | -0.370483000 | -1.347821000 |
| 6 | -11.516902000 | -0.638349000 | 0.081251000 |
| 1 | -11.711419000 | 0.438511000 | 0.182079000 |
| 1 | -11.649354000 | -1.086921000 | 1.075704000 |
| 8 | 9.254544000 | 0.137507000 | -0.675506000 |
| 6 | 10.137424000 | -0.415023000 | 0.367619000 |
| 1 | 9.929141000 | -1.488438000 | 0.485973000 |
| 1 | 9.934345000 | 0.094031000 | 1.321115000 |
| 6 | 11.578149000 | -0.180944000 | -0.083469000 |
| 1 | 11.739694000 | 0.896874000 | -0.224206000 |
| 1 | 11.732100000 | -0.662826000 | -1.059073000 |
| 6 | -12.525701000 | -1.260429000 | -0.910617000 |
| 1 | -12.380186000 | -0.817399000 | -1.907657000 |
| 1 | -12.322910000 | -2.337403000 | -1.012426000 |
| 6 | -13.988179000 | -1.053152000 | -0.465289000 |
| 1 | -14.226237000 | 0.016264000 | -0.384875000 |
| 1 | -14.168551000 | -1.511931000 | 0.516467000 |
| 1 | -14.688572000 | -1.502092000 | -1.181528000 |
| 6 | 12.598477000 | -0.732768000 | 0.937816000 |

| | | | |
|---|---|---|---|
| 1 | 12.431079000 | -0.258027000 | 1.916661000 |
| 1 | 12.429451000 | -1.811222000 | 1.078387000 |
| 6 | 14.057030000 | -0.495253000 | 0.494769000 |
| 1 | 14.765722000 | -0.895567000 | 1.231373000 |
| 1 | 14.259160000 | -0.983230000 | -0.468496000 |
| 1 | 14.261931000 | 0.577593000 | 0.377180000 |

**[4c]$^+$**

| | | | |
|---|---|---|---|
| 7 | 1.207371000 | 1.520096000 | -0.087437000 |
| 7 | 1.770840000 | -1.327113000 | 0.013887000 |
| 7 | -1.634337000 | 0.958432000 | -0.063568000 |
| 7 | -1.072466000 | -1.889018000 | 0.006398000 |
| 6 | 0.729321000 | 2.835452000 | -0.050981000 |
| 6 | 1.860635000 | 3.727600000 | 0.075266000 |
| 6 | 3.000729000 | 2.962557000 | 0.091233000 |
| 6 | 2.603432000 | 1.576461000 | -0.017321000 |
| 1 | 1.787290000 | 4.800213000 | 0.159685000 |
| 1 | 4.019489000 | 3.303886000 | 0.188719000 |
| 6 | 3.494375000 | 0.491508000 | -0.041023000 |
| 6 | -0.611367000 | 3.252466000 | -0.126714000 |
| 6 | 3.978271000 | -1.983400000 | -0.156559000 |
| 6 | 3.214576000 | -3.123457000 | -0.125230000 |
| 6 | 1.828453000 | -2.724777000 | -0.007641000 |
| 6 | 3.084286000 | -0.849703000 | -0.059886000 |
| 1 | 5.050605000 | -1.913999000 | -0.252457000 |
| 1 | 3.555679000 | -4.144677000 | -0.191873000 |
| 6 | 0.745797000 | -3.615077000 | 0.070700000 |
| 6 | -2.946246000 | 0.476351000 | -0.125970000 |
| 6 | -3.838525000 | 1.599874000 | -0.306510000 |
| 6 | -3.074936000 | 2.740553000 | -0.337000000 |
| 6 | -1.691375000 | 2.353055000 | -0.171321000 |
| 1 | -4.909113000 | 1.523483000 | -0.414778000 |
| 1 | -3.413560000 | 3.754903000 | -0.476587000 |
| 6 | -1.726528000 | -4.088073000 | 0.265533000 |
| 6 | -2.866652000 | -3.324330000 | 0.236412000 |
| 6 | -2.469683000 | -1.943616000 | 0.063191000 |
| 6 | -0.594768000 | -3.199867000 | 0.110432000 |
| 1 | -1.654272000 | -5.156415000 | 0.396260000 |
| 1 | -3.885993000 | -3.662185000 | 0.340052000 |
| 6 | -3.358646000 | -0.862833000 | -0.032952000 |
| 6 | 4.966874000 | 0.784275000 | -0.046851000 |
| 6 | 5.568798000 | 1.392331000 | -1.171495000 |
| 6 | 6.945365000 | 1.660964000 | -1.186270000 |
| 6 | 7.730363000 | 1.327456000 | -0.064988000 |
| 6 | 7.144907000 | 0.727211000 | 1.065212000 |
| 6 | -0.904340000 | 4.720268000 | -0.154702000 |
| 6 | -1.656646000 | 5.327085000 | 0.875420000 |
| 6 | -1.938294000 | 6.702205000 | 0.870268000 |
| 6 | -1.473003000 | 7.508006000 | -0.196194000 |
| 6 | -0.721069000 | 6.908464000 | -1.236270000 |
| 6 | -0.437044000 | 5.537616000 | -1.210574000 |
| 1 | -2.013186000 | 4.723654000 | 1.706214000 |
| 1 | -2.506314000 | 7.151624000 | 1.672940000 |
| 1 | 0.132129000 | 5.098341000 | -2.025314000 |
| 6 | -4.831570000 | -1.151822000 | -0.041420000 |
| 6 | -5.648712000 | -0.737747000 | 1.033930000 |
| 6 | -7.025923000 | -1.006548000 | 1.029386000 |
| 6 | -7.595995000 | -1.690655000 | -0.061205000 |
| 6 | -6.795886000 | -2.106145000 | -1.142910000 |
| 6 | -5.418197000 | -1.841140000 | -1.126147000 |
| 6 | 1.036235000 | -5.086750000 | 0.114117000 |
| 6 | 1.704845000 | -5.652206000 | 1.224708000 |
| 6 | 1.971465000 | -7.027804000 | 1.268802000 |
| 6 | 1.581844000 | -7.859453000 | 0.195919000 |
| 6 | 0.918479000 | -7.301569000 | -0.918619000 |
| 6 | 0.644578000 | -5.925951000 | -0.954103000 |
| 1 | 2.000404000 | -5.019016000 | 2.056307000 |
| 1 | 2.476771000 | -7.464960000 | 2.124389000 |
| 1 | 0.624047000 | -7.940242000 | -1.743844000 |
| 1 | 0.140421000 | -5.500608000 | -1.817181000 |
| 6 | 5.768342000 | 0.451977000 | 1.067376000 |
| 6 | 1.886925000 | -9.311367000 | 0.276927000 |
| 8 | 2.471264000 | -9.871634000 | 1.226582000 |
| 1 | -0.360842000 | 7.514483000 | -2.064875000 |
| 6 | -2.422689000 | 9.721669000 | 0.577100000 |
| 8 | -2.972807000 | 9.300750000 | 1.633451000 |
| 7 | -1.717616000 | 8.902928000 | -0.289280000 |

| | | | |
|---|---|---|---|
| 1 | -1.322062000 | 9.357721000 | -1.105559000 |
| 8 | 1.453490000 | -10.018595000 | -0.828966000 |
| 1 | 1.666931000 | -10.976635000 | -0.752988000 |
| 6 | -2.499111000 | 11.183653000 | 0.168306000 |
| 1 | -2.118658000 | 11.804269000 | 0.986889000 |
| 1 | -1.934677000 | 11.412273000 | -0.740750000 |
| 1 | -3.548645000 | 11.456775000 | 0.009477000 |
| 1 | -5.208448000 | -0.218079000 | 1.879587000 |
| 1 | -7.640925000 | -0.693924000 | 1.867132000 |
| 1 | -7.235430000 | -2.633519000 | -1.983565000 |
| 1 | -4.802628000 | -2.157930000 | -1.962637000 |
| 1 | 5.317186000 | -0.009327000 | 1.940694000 |
| 1 | 4.966154000 | 1.641584000 | -2.039609000 |
| 1 | 7.749593000 | 0.470012000 | 1.928712000 |
| 1 | 7.398101000 | 2.114762000 | -2.062415000 |
| 79 | 0.067907000 | -0.184482000 | -0.032520000 |
| 6 | -9.072064000 | -1.937175000 | -0.094938000 |
| 9 | -9.794955000 | -0.893033000 | -0.726149000 |
| 9 | -9.421819000 | -3.102382000 | -0.798285000 |
| 9 | -9.642221000 | -2.059286000 | 1.182872000 |
| 6 | 9.190643000 | 1.656585000 | -0.069281000 |
| 9 | 9.457007000 | 3.006810000 | 0.271910000 |
| 9 | 9.936051000 | 0.890087000 | 0.838967000 |
| 9 | 9.791670000 | 1.474594000 | -1.328540000 |

**4c**

| | | | |
|---|---|---|---|
| 7 | 1.307364000 | 1.524662000 | -0.108605000 |
| 7 | 1.766081000 | -1.443590000 | 0.019268000 |
| 7 | -1.659033000 | 1.068881000 | -0.035021000 |
| 7 | -1.200997000 | -1.902239000 | -0.015977000 |
| 6 | 0.867897000 | 2.842461000 | -0.084614000 |
| 6 | 2.045877000 | 3.700354000 | 0.014189000 |
| 6 | 3.156542000 | 2.886837000 | 0.036323000 |
| 6 | 2.694132000 | 1.503496000 | -0.047431000 |
| 1 | 2.027497000 | 4.777766000 | 0.081697000 |
| 1 | 4.186932000 | 3.198108000 | 0.123967000 |
| 6 | 3.520748000 | 0.346365000 | -0.040778000 |
| 6 | -0.483119000 | 3.286482000 | -0.126339000 |
| 6 | 3.941142000 | -2.185676000 | -0.105575000 |
| 6 | 3.127881000 | -3.295985000 | -0.081702000 |
| 6 | 1.744873000 | -2.831691000 | 0.005817000 |
| 6 | 3.082758000 | -1.005004000 | -0.034581000 |
| 1 | 5.018148000 | -2.172459000 | -0.183714000 |
| 1 | 3.438172000 | -4.328842000 | -0.136848000 |
| 6 | 0.589046000 | -3.658270000 | 0.047856000 |
| 6 | -2.974624000 | 0.627889000 | -0.078650000 |
| 6 | -3.832302000 | 1.801454000 | -0.226046000 |
| 6 | -3.018685000 | 2.911648000 | -0.259670000 |
| 6 | -1.637208000 | 2.454864000 | -0.131116000 |
| 1 | -4.908477000 | 1.783086000 | -0.314100000 |
| 1 | -3.325930000 | 3.939548000 | -0.380858000 |
| 6 | -1.941164000 | -4.071934000 | 0.185540000 |
| 6 | -3.051440000 | -3.258496000 | 0.167613000 |
| 6 | -2.588825000 | -1.878731000 | 0.032161000 |
| 6 | -0.762296000 | -3.217154000 | 0.061805000 |
| 1 | -1.925399000 | -5.146468000 | 0.291565000 |
| 1 | -4.082393000 | -3.566726000 | 0.259004000 |
| 6 | -3.413933000 | -0.723452000 | -0.019986000 |
| 6 | 5.006198000 | 0.577009000 | -0.037806000 |
| 6 | 5.641437000 | 1.191764000 | -1.141257000 |
| 6 | 7.028331000 | 1.404510000 | -1.147069000 |
| 6 | 7.797298000 | 1.004080000 | -0.037167000 |
| 6 | 7.182017000 | 0.394862000 | 1.074160000 |
| 6 | -0.711116000 | 4.770103000 | -0.168619000 |
| 6 | -1.387923000 | 5.435494000 | 0.877562000 |
| 6 | -1.602259000 | 6.824059000 | 0.856496000 |
| 6 | -1.142134000 | 7.585553000 | -0.243200000 |
| 6 | -0.468296000 | 6.929679000 | -1.301476000 |
| 6 | -0.252408000 | 5.546077000 | -1.258953000 |
| 1 | -1.739227000 | 4.864860000 | 1.733514000 |
| 1 | -2.113356000 | 7.316096000 | 1.672556000 |
| 1 | 0.260638000 | 5.062424000 | -2.085789000 |
| 6 | -4.899767000 | -0.951295000 | -0.018651000 |
| 6 | -5.697398000 | -0.498642000 | 1.057542000 |
| 6 | -7.084221000 | -0.711252000 | 1.065206000 |
| 6 | -7.690576000 | -1.380687000 | -0.015681000 |
| 6 | -6.913636000 | -1.834455000 | -1.099471000 |

| | | | |
|---|---|---|---|
| 6 | -5.526621000 | -1.623654000 | -1.093114000 |
| 6 | 0.818154000 | -5.143244000 | 0.082982000 |
| 6 | 1.464402000 | -5.747413000 | 1.188046000 |
| 6 | 1.674251000 | -7.132822000 | 1.225109000 |
| 6 | 1.248913000 | -7.943703000 | 0.148918000 |
| 6 | 0.607184000 | -7.350366000 | -0.960905000 |
| 6 | 0.391581000 | -5.964480000 | -0.987410000 |
| 1 | 1.786453000 | -5.131281000 | 2.022648000 |
| 1 | 2.161862000 | -7.594542000 | 2.078335000 |
| 1 | 0.284986000 | -7.970865000 | -1.789917000 |
| 1 | -0.094971000 | -5.512833000 | -1.847189000 |
| 6 | 5.795672000 | 0.179903000 | 1.066142000 |
| 6 | 1.492525000 | -9.404872000 | 0.221063000 |
| 8 | 2.051812000 | -9.999837000 | 1.166257000 |
| 1 | -0.114455000 | 7.501893000 | -2.156662000 |
| 6 | -1.931424000 | 9.866494000 | 0.521615000 |
| 8 | -2.460532000 | 9.501675000 | 1.610437000 |
| 7 | -1.315651000 | 8.992907000 | -0.355127000 |
| 1 | -0.930080000 | 9.408040000 | -1.197056000 |
| 8 | 1.031495000 | -10.090153000 | -0.890263000 |
| 1 | 1.206886000 | -11.055805000 | -0.814556000 |
| 6 | -1.931982000 | 11.324462000 | 0.088697000 |
| 1 | -1.417325000 | 11.923642000 | 0.848361000 |
| 1 | -1.448816000 | 11.491760000 | -0.878821000 |
| 1 | -2.966399000 | 11.681639000 | 0.033626000 |
| 1 | -5.229959000 | 0.007260000 | 1.896872000 |
| 1 | -7.679732000 | -0.368663000 | 1.905699000 |
| 1 | -7.379406000 | -2.349295000 | -1.934030000 |
| 1 | -4.930168000 | -1.969671000 | -1.931842000 |
| 1 | 5.322389000 | -0.285654000 | 1.925310000 |
| 1 | 5.051896000 | 1.489810000 | -2.002984000 |
| 1 | 7.772393000 | 0.085819000 | 1.931256000 |
| 1 | 7.501892000 | 1.865699000 | -2.008092000 |
| 79 | 0.053409000 | -0.188106000 | -0.034963000 |
| 6 | -9.173156000 | -1.568534000 | -0.036275000 |
| 9 | -9.866997000 | -0.493523000 | -0.652441000 |
| 9 | -9.578747000 | -2.714339000 | -0.743732000 |
| 9 | -9.738156000 | -1.677262000 | 1.246659000 |
| 6 | 9.268049000 | 1.269037000 | -0.015324000 |
| 9 | 9.606058000 | 2.508626000 | 0.589636000 |
| 9 | 9.996156000 | 0.304897000 | 0.705409000 |
| 9 | 9.843651000 | 1.320425000 | -1.296527000 |
